# Mismatched and wobble base pairs govern primary microRNA processing by human Microprocessor

**DOI:** 10.1038/s41467-020-15674-2

**Published:** 2020-04-21

**Authors:** Shaohua Li, Trung Duc Nguyen, Thuy Linh Nguyen, Tuan Anh Nguyen

**Affiliations:** Division of Life Science, The Hong Kong University of Science and Technology, Hong Kong, China

**Keywords:** Biochemistry, Molecular biology

## Abstract

MicroRNAs (miRNAs) are small RNAs that regulate gene expression. miRNAs are produced from primary miRNAs (pri-miRNAs), which are cleaved by Microprocessor. Microprocessor, therefore, plays a crucial role in determining the efficiency and precision of miRNA production, and thus the function of the final miRNA product. Here, we conducted high-throughput enzymatic assays to investigate the catalytic mechanism of Microprocessor cleaving randomized pri-miRNAs. We identified multiple mismatches and wobble base pairs in the upper stem of pri-miRNAs, which influence the efficiency and accuracy of their processing. The existence of these RNA elements helps to explain the alternative cleavage of Microprocessor for some human pri-miRNAs. We also demonstrated that miRNA biogenesis can be altered via modification of the RNA elements by RNA-editing events or single nucleotide polymorphisms (SNPs). These findings improve our understanding of pri-miRNA processing mechanisms and provide a foundation for interpreting differential miRNA expression due to RNA modifications and SNPs.

## Abstract (web summary)

MicroRNA genes are transcribed to long primary transcripts called primary microRNAs, which are cleaved by Microprocessor. Here the authors employ high-throughput sequencing and Microprocessor assay to show that mismatches and wobble base pairs in primary microRNAs affect the accuracy and efficiency of Microprocessor processing.

## Introduction

MicroRNAs (miRNAs) play an important role in gene regulation as they trigger the degradation of and/or inhibit the translation of mRNA^1–3^. Primary miRNA transcripts (pri-miRNAs) are synthesized in the nucleus by RNA polymerase II^4,5^. Most pri-miRNAs are processed in tandem by two RNase III enzymes, called Microprocessor and DICER^4,5^. Pri-miRNAs are first cleaved by Microprocessor to generate miRNA precursors (pre-miRNAs), and these are then exported to the cytoplasm where they are further cleaved by DICER to produce 21–22 nt RNA duplexes^4,5^. These RNA duplexes are subsequently taken up by Ago proteins. One of the two RNA strands is selected to become the mature miRNA sequence and form an Ago-miRNA complex, whereas the other so-called passenger strand is discarded^3,6^. The Ago-miRNA complex is a core component of the miRNA-mediated RNA silencing machinery, which targets mRNAs to knock down gene expression^3,5–7^. The processing of pri-miRNAs by Microprocessor is thus critical to the expression and ultimately the function of miRNAs.

**Fig. 1:**
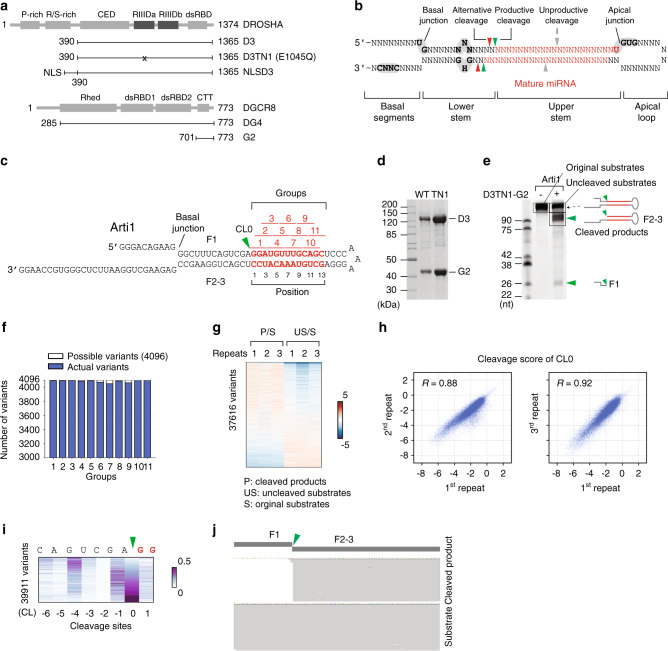
High-throughput pri-miRNA processing assays. **a** The protein constructs used in this study. The first and last residue numbers for each construct are shown. The mutation site is marked with an ‘x’. P-rich: Proline-rich domain; R/S-rich: Arginine/serine-rich domain; CED: Central domain; RIIIDa and RIIIDb: RNase III domains; dsRBD: double-stranded RNA-binding domain; Rhed: RNA-binding heme domain; and CTT: C-terminal tail region. **b** The structure of a typical pri-miRNA, which is the substrate for Microprocessor, has a stem-loop configuration. The green, red, and gray arrowheads indicate the productive, alternative, and unproductive cleavage sites of DROSHA. The red letters indicate the miRNA sequence. The letters highlighted in gray show the identified RNA motifs. **c** Structure of the arti1 substrate. The green arrowhead indicates the DROSHA cleavage site on the 5p-strand, and the red letters represent the randomized region, which is divided into 11 groups. Each group contains 3 pairs of randomized nucleotides. **d** SDS-PAGE to show the purity of the D3-G2 and D3TN1-G2 complexes. **e** Processing of arti1 by D3TN1-G2. Arti1 (12 pmol) was incubated with D3TN1-G2 (20 pmol) for 120 min at 37 °C in 10 μl standard reaction buffer, as described in the Methods section. **f** Stacked bar graph to show the total number of arti1 variants in each group. The total numbers of actual variants from 3 repeats are shown in blue, whereas the theoretical numbers of the possible variants are shown in white. The theoretical variant number of each group is 4^6^ (4096). **g** The cleavage score of D3TN1-G2 estimated from 3 repeats, colored according to the color bar (right). P: cleaved products; S: original substrates; and US: uncleaved substrates. **h** The reproducibility of the 3 repeats. The CL0 cleavage score of one repeat was plotted against that of another. The CL0 is the canonical cleavage site. **i** The relative positional cleavage frequency of DROSHA at different positions ranging from 7 to 14 nt from the basal junction of each variant, colored according to the color bar (right). Number “0” indicates the cleavage site 13 nt from the basal junction. The cleaved products that resulted from each variant substrate were collected, and their relative percentage was estimated between 0 and 1. **j** The representative substrate and product sequence of a variant visualized by IGV^47^. The reads resulting from the NGS were aligned to the referenced sequence of arti1. The source data are provided in the Source Data file.

Human Microprocessor is a trimeric protein complex, which is composed of an RNase III enzyme, DROSHA, and DGCR8^8–16^ (Fig. 1a). The typical Microprocessor substrate, pri-miRNA, is modeled as a stem-loop-containing RNA molecule^17^ (Fig. 1b). The stem is an imperfect double-stranded region of RNA comprising ~35 base pairs (bp)^17–19^. The loop (also known as the apical loop) is variable in length and connects to the stem at its apical junction. Another part of the pri-miRNA structure is called the basal junction, this is formed between the stem and the 5p-basal and 3p-basal RNA segments^17–19^. The stem is sub-divided by DROSHA cleavage sites into the upper and lower stems, and both DROSHA and DGCR8 are coordinated to interact with multiple RNA elements to process pri-miRNA accurately and efficiently^4,5,20^.

DROSHA is known to cleave at the basal junction (referred to as ‘productive’ cleavage); it measures the distance and determines the site of cleavage at ~13 nt from the basal junction^13,14,17^. It has been suggested that DROSHA might also be able to cleave at the apical junction via a process called ‘unproductive’ cleavage^13,14,17,20^ (Fig. 1b). For this reason, multiple RNA-protein interactions ensure that DROSHA only cleaves at productive sites at the basal junction. This is achieved because DROSHA interacts with a UG motif located at the basal junction. This strengthens the interaction between DROSHA and the junction, and thus enhances its cleavage efficiency and accuracy^13,21^. In addition, DROSHA has a double-stranded RNA binding domain (dsRBD), which it uses to interact with an mGHG motif located in the lower stem of the pri-miRNA to precisely find the correct cleavage sites^18,22^. DGCR8 in a dimer form also plays an essential role in ensuring that DROSHA cleaves accurately ~22 nt from the apical junction. This dimer interacts with the apical loop and the apical UGU motif, and in this way occupies a distinct region of the upper stem of pri-miRNA^12,13,21,23^. This prevents DROSHA from being mislocalized in the upper stem, and thus ensures that it cleaves the pri-miRNA precisely at the basal junction. The interaction between DGCR8 and the UGU-containing apical loop is strengthened by a small molecule, called hemin^16,24^.

Another factor that controls the action of Microprocessor is the length of the pri-miRNA stem region^18,25^. As mentioned above, DROSHA precisely measures the site of cleavage to be ~13 nt from the basal junction, and DGCR8 interacts with the loop and apical UGU motif located ~22 nt from the cleavage sites. This means that the optimal length of the pri-miRNA stem for cleavage by Microprocessor is ~35 bp. However, when the stem is more or less than 35 bp in length, then this counteracts the coordinated positioning between DROSHA and DGCR8 and results in inaccurate or alternative cleavages^25^ (Fig. 1b). The splicing factor, SRSF3, stimulates Microprocessor activity by recruiting DROSHA to the basal junction via interaction with CNNC motifs localized 16–18 nt along the 3p-basal RNA segment^21,26,27^. SRSF3 can also change the cleavage site of DROSHA, whereby the CNNC motif is located beyond the 16–18 nt region^27^. These numerous different mechanisms should guarantee that Microprocessor cleaves at the correct locations. However, many pri-miRNAs exhibit various alternative cleavages, which suggests that unknown RNA elements or protein factors might influence the choice of cleavage sites by Microprocessor.

Various RNA elements have been identified at different locations on pri-miRNAs, which are known to be recognized by and interact with many protein domains of DROSHA and DGCR8^13,14,16–18,21–23^. However, the contribution of the pri-miRNA upper stem to the enzyme-substrate interaction mechanism is still not known. Here, we set up a high-throughput in vitro pri-miRNA processing screen to investigate the interaction between DROSHA and the upper stem of pri-miRNAs. Approximately ~200,000 randomized pri-miRNAs were assayed with purified DROSHA protein, and the substrates and cleaved products were sequenced by next-generation sequencing (NGS) and then analyzed using a bioinformatics approach. We identified multiple mismatches and wobble base pairs in the upper stem, which regulate the efficiency and accuracy of pri-miRNA processing by DROSHA. The presence of these RNA elements helps to explain why several pri-miRNAs undergo alternative cleavages. We also showed that the RNA elements can be modified due to RNA-editing events and single nucleotide polymorphisms (SNPs), which results in altered miRNA expression.

## Results

### High-throughput pri-miRNA processing assays

Since DROSHA can cleave either the basal or apical junction of a typical two-junction pri-miRNA^13,14,20,27^, we decided to use the short pri-miRNA substrate, arti1, which contains just one junction, to investigate the substrate recognition ability of DROSHA (Fig. 1c). We also used the pri-mir-16-1 backbone with different upper stem lengths to test the cleavage activity of DROSHA. We showed that the enzyme was more effective at cleaving the pri-miRNA with the 10 bp upper stem than it was at cleaving the pri-miRNA with the 8 bp upper stem, but further increases in the stem length (i.e., to 12, 14 or 24 bp) did not enhance the enzyme activity of DROSHA (Supplementary Fig. 1a, b). Therefore, DROSHA likely requires an upper stem length of ~10 bp to most efficiently cleave pri-miRNAs. In addition, the structure model of DROSHA with dsRNA indicates that the elongated conformation of DROSHA might cover 10–12 bp of the upper stem^14^. For this reason, we designed the arti1 substrate to have an upper stem of 17 bp (Fig. 1c). In addition, randomized sequences in 3-nt windows were introduced into the arti1 backbone, as indicated in Fig. 1c. These were located between positions 1 to 13 downstream of the canonical cleavage site of DROSHA, which is 13 nt from the basal junction. In our in vitro processing assays, we used a total of 11 substrate groups (Fig. 1c), and to obtain as many substrate variants as possible, we synthesized a high quantity of RNAs and used 6 pmol (~3.6 trillion molecules) of the RNA substrates for each group.

DROSHA has two RNase III domains (RIIIa and RIIIb), which cleave the 3p-strand and 5p-strand of pri-miRNAs, respectively (Fig. 1a, b). We utilized the mutant form of DROSHA, called DROSHA-TN1, which possesses a mutation (E1045Q) at the catalytic site of RIIIa^11,13^ (Fig. 1a). This maintains normal cleavage activity (making a single cut alone) on the 5p-strand of the pri-miRNA stem but does not cleave the 3p-strand. Consequently, using the DROSHA-TN1 in the cleavage assays, both the original RNA substrates and the cleaved products contain randomized sequences for further analysis. In addition, with DROSHA-TN1 we could use a specific reverse primer for both the substrates and the cleaved products in the reverse transcription step. In this way, we could circumvent a 3p-end ligation step during RNA cloning, which helped to reduce any possible biases caused by this step. DROSHA cannot be purified as a single subunit as it aggregates considerably when it is overexpressed in human cells^13^. Therefore, we purified a minimal Microprocessor complex containing DROSHA-TN1 (D3TN1, amino acids 390–1365) and DGCR8 (G2, amino acids 701–773) fragments (Fig. 1d). It is known that the D3TN1 fragment contains the necessary DROSHA domains to maintain its normal cleavage activity, and that the G2 fragment alone does not have any RNA-binding affinity^13,14^. For this reason, the RNA-recognition capacity of the D3TN1-G2 complex is likely to be from DROSHA^13^. As expected, the D3TN1-G2 complex only cleaved at the 5p-strand of pri-mir-16-1, generating F1 and F2-3 fragments (Supplementary Fig. 1c, lane 3); whereas the wild-type (WT) D3-G2 complex made double cleavages on pri-mir-16-1, and generated F1, F2, and F3 fragments (Supplementary Fig. 1c, lane 2). Consistently, we also found that D3TN1-G2 only created a single cut on the arti1 substrate, generating two fragments, F1 and F2-3 (Fig. 1e, Supplementary Fig. 1d, e). Subsequently, we performed processing assays for each of the 11 arti1 substrate groups using 6 pmol of RNA and 10 pmol of D3TN1-G2. The reaction mixtures for each group were separately analyzed via 10% urea-PAGE. The original substrates, uncleaved substrates, and cleaved products were gel-eluted, purified, and cloned to generate three DNA libraries (Supplementary Fig. 1f), and each experiment was conducted in triplicate. The resulting nine libraries were then subjected to Illumina HiSeq X Ten sequencing, using the HiSeq X Ten Reagent Kit v2.5 (Illumina) with the sequencer in pair-end running mode. We collected ~169 million reads for these libraries.

We also performed high-throughput pri-miRNA processing for the D3TN1-G2 and D3-G2 complexes, as well as for the randomized pri-miRNAs, using the pri-mir-342, pri-mir-106a, or pri-mir-514a-1 backbone, as described in the Methods section. We collected ~190 million reads for these libraries.

### DROSHA exhibits multiple cleavage sites

We obtained between 4045 and 4096 variants for each arti1 group in three repeats, which each theoretically contains 4096 (4^6^) variants (Fig. 1f). The median read number for each variant was ~250, and ~95% of the variants contained more than 15 reads (Supplementary Fig. 1g). This indicates that the arti1 substrates were adequately prepared for the high-throughput pri-miRNA processing assays. Subsequently, we estimated the cleavage score for each variant by calculating the ratio of the cleaved products (P) to their original substrate (S). We also estimated the reciprocal cleavage score for each variant by calculating the ratio of the uncleaved substrate (US) to the original substrate. As the results show (Fig. 1g), the reverse patterns obtained for P/S and US/S for the majority of the arti1 variants indicate that the DROSHA-cleaving conditions were reliable for further analysis. In addition, there was a high correlation between the three repeats, suggesting the reproducibility of our high-throughput enzymology experiments (Fig. 1h).

We then mapped the cleavage products to the substrate sequences to identify the cleavage sites of DROSHA. We labeled the cleavage sites according to their distance from the canonical cleavage site; thus, they were labeled CL (for cleavage site) followed by a number (Fig. 1h, i). For each variant from each group (G1 to G11), we calculated the relative positional cleavage frequency at different positions (CL-6 to CL1). In brief, the relative positional cleavage frequency for each variant at a particular position was estimated as the percentage of the cleaved product read counts at this position over the sum of the cleaved product read counts at all the positions resulting from this variant. We found that the major cleavage site for most variants was canonical CL0, which is 13 nt from the basal junction (Fig. 1i, j). This is consistent with previous studies^13,14,17,18,21,22^. However, we also found that numerous variants were cleaved at non-canonical sites, ranging from CL-6 to CL1 despite the constant lower stem sequence and structure (Fig. 1i). This implies that unknown RNA elements in the upper stem of pri-miRNAs might cause alternative cleavages.

We performed a similar analysis for data acquired from the high-throughput pri-miRNA processing of pri-mir-514a-1, pri-mir-106a, and pri-mir-342, and we demonstrated that DROSHA mainly cleaved these pri-miRNAs at the canonical sites. However, DROSHA also cleaved at alternative sites on these pri-miRNAs and it exhibited more alternative cleavages on pri-mir-106a and pri-mir-342 than it did on pri-mir-514a-1 (Supplementary Fig. 1h, i). As pri-mir-514a-1 contains UG, UGU, and mGHG motifs, these might together help confine the cleavage activity of DROSHA at the canonical sites. In contrast, pri-mir-106a and pri-mir-342 do not contain these motifs, and so we hypothesized that other unknown RNA elements in the upper stem of these two pri-miRNAs might contribute to the alternative cleavages that occur.

### MidMW causes DROSHA to cleave at alternative sites

**Fig. 2:**
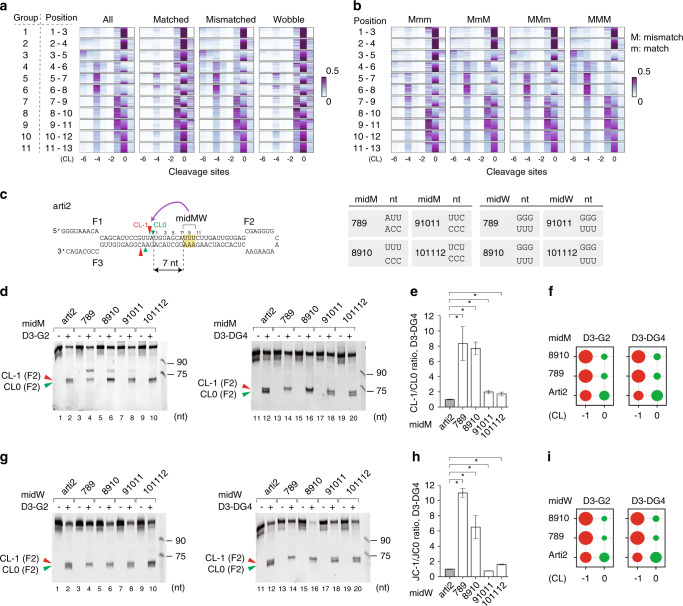
MidMW causes the alternative cleavage of DROSHA. **a** The relative positional cleavage frequency of DROSHA at different positions ranging from 7 to 14 nt from the basal junction of each variant from an arti1 individual group, colored according to the color bar (right). Number “0” indicates the cleavage site 13 nt from the basal junction. The positional cleavage frequency was estimated for all the variants of each group. It was also estimated for three subgroups which contain the matched, mismatched, and wobble variants. **b** The relative positional cleavage frequency of DROSHA was estimated for each variant containing a different number of mismatches ranging from 1 to 3, colored according to the color bar (right). **c** Diagram showing the various arti2 variants with mismatches or wobble base pairs at different positions. The red and green arrowheads indicate the CL-1 and CL0 cleavage sites of DROSHA. The table shows the various arti2 variants with mismatches and wobble base pairs at different positions. **d**, **g** Processing of arti2 by D3-G2 or D3-DG4. The arti2 variants contain mismatches (**d**) and wobble base pairs (**g**) at different positions. Each RNA (6 pmol) was incubated with D3-G2 (10 pmol) or D3-DG4 (6 pmol) for 120 min at 37 °C in 10 μl standard reaction buffer as described in the Methods section. **e**, **h** The estimated CL-1 to CL0 cleavage ratio of D3-DG4 from experiments conducted in triplicate. The band densities of the F2 fragments resulting from the CL-1 and CL0 cleavage sites were measured using Image Lab v.6.0.1. Data are presented as mean values +/− SEM. The asterisks (*) indicate statistical significant differences from the two-sided *t*-test (midM_789 vs. arti2: *p* = 0.031, midM_8910 vs. arti2: *p* = 0.001, midM_91011 vs. arti2: *p* = 0.013, midM_101112 vs. arti2: *p* = 0.050, midW_789 vs. arti2: *p* = 7.8e-5, midW_8910 vs. arti2: *p* = 0.022, midW_91011 vs. arti2: *p* = 0.010, midW_101112 vs. arti2: *p* = 0.002). **f**, **i** The F2 fragments resulting from the CL-1 and CL0 cleavage sites were cloned and sequenced by NGS. The size of the circle indicates the relative amounts of each F2 fragment from NGS data. The source data are provided in the Source Data file.

We examined the cleavage site patterns of the 11 arti1 groups and observed their distinct cleavage patterns (Fig. 2a, left panel). Groups G1, G2, G10, and G11 had a similar cleavage pattern such that the main cleavage site was CL0. In contrast, groups G3–G9 had cleavage sites at multiple locations. The major alternative cleavage sites for these groups were as follows: G3 = CL-6, G4–6 = CL-4, and G7–9 = CL-1 (Fig. 2a, left panel). This suggests that the randomized sequences located between positions 3 to 11 in the upper stem might cause the alternative cleavages of DROSHA. To determine which RNA features in each group contributed to the alternative cleavages, we further separated the variants in each group into matched, mismatched, and wobble (G–U or U–G) subgroups. The variants of the mismatched and wobble subgroups contained 1 to 3 mismatches and 1 to 3 wobble base pairs, respectively. We found that mismatches in groups G3–9 largely stimulated the alternative cleavages (Fig. 2a). For example, cleavage at CL-4 was strikingly enhanced by mismatches in groups G5 and G6. In addition, cleavage at CL-1 was markedly augmented by mismatches in groups G7–9 (Fig. 2a, right panel). This suggests that these mismatches might induce DROSHA to cleave at alternative sites. We further separated the mismatched variants into four subgroups, which contained a single mismatch followed by two matches (Mmm); two mismatches followed by one match (MMm); two mismatches separated by one match (MmM); and triple mismatches (MMM), within each 3-nt window (Fig. 2b). We demonstrated that within positions 7–10 the triple and double mismatches caused more alternative cleavages of CL-1 than the single mismatch, but within positions 1–3 the mismatches had more precise cleavage at CL0 (Fig. 2b). We similarly found that wobble base pairs at positions 7–10 showed more alternative cleavages at CL-1, and those at positions 5–7 and 6–8 exhibited more alternative cleavages at CL-4 (Fig. 2a). We also found that G-U and U-G pairs in positions 7–10 had a similar effect on enhancing CL-1 cleavage (Supplementary Fig. 2a, b). As the mismatches and wobble base pairs were located in the middle of miRNA sequences embedded in the pri-miRNAs, we have called them ‘middle mismatched and wobble base pairs’ or ‘midMW’.

We synthesized another artificial pri-miRNA backbone, arti2, which contained two junctions (Fig. 2c). The arti2 sequence is similar to that used in previous studies^13,14^, except that Watson-Crick base pairs were used in place of wobble base pairs in the upper stem. We showed that arti2 was frequently cleaved at both CL-1 and CL0 by D3-G2 and D3-DG4 (Fig. 2d, lanes 2 and 12). As D3-DG4 contained a long fragment of DGCR8 (DG4, amino acids 285–773), and this long fragment contains the essential RNA-binding domains of DGCR8, D3-DG4 was considered to be a functionally active Microprocessor complex^13,14,16,23^. Consistent with the arti1 cleavage pattern, we found with arti2, that triple mismatches or wobble base pairs at positions 7–9 and 8–10 strongly stimulated CL-1 cleavages by D3-G2 and D3-DG4 (Fig. 2d–i), whereas those at positions 9–11 and 10–12 had a less profound effect on the cleavage of CL-1 (Fig. 2d–i). Interestingly, a single mismatch at position 8 also substantially introduced cleavage at CL-1 (Supplementary Fig. 2c–e). In addition, mismatches at positions 7–9 and 8–10 also induced D3-G2 to cleave at other positions from CL-3 to CL-6 (Fig. 2d, lanes 4 and 6). However, these cleavages were completely blocked by DGCR8 in the pri-miRNA processing assays with D3-DG4 (Fig. 2d, lanes 14 and 16). For this reason, we did not investigate these near-junction cleavages any further. Together, these data showed that mismatches and wobble base pairs in the pri-miRNA upper stem were able to facilitate the alternative cleavages of DROSHA.

### MidMW influences the cleavage sites on human pri-miRNAs

**Fig. 3:**
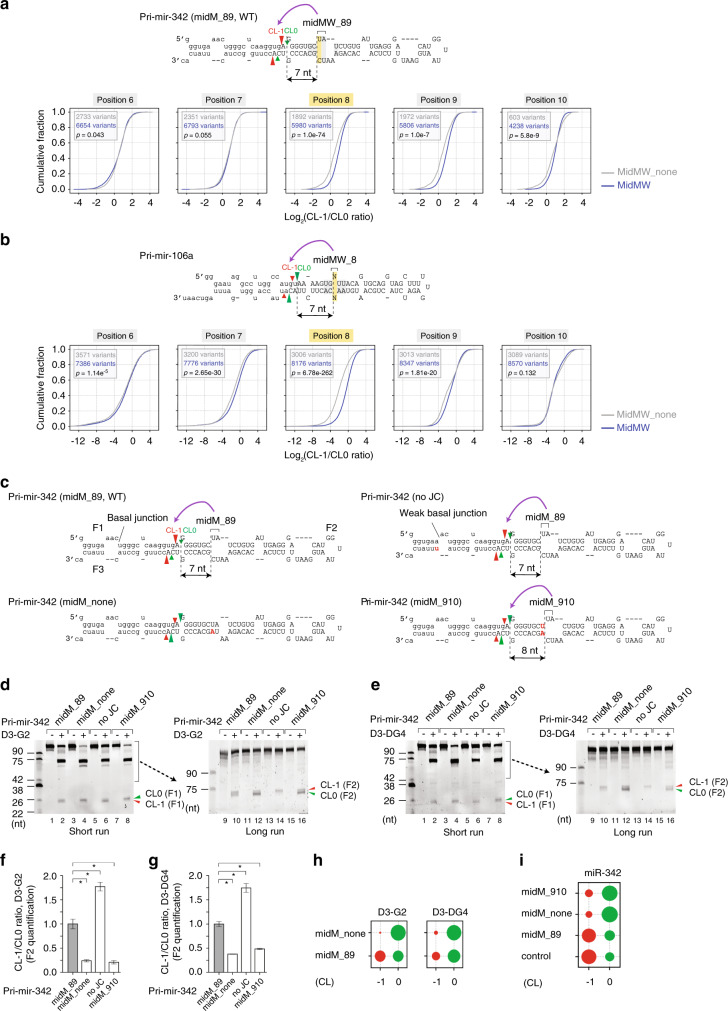
MidMW influences the choice of the cleavage site of Microprocessor on human pri-miRNAs. **a**, **b** Cumulative plots showing the cleavage site choices of DROSHA on the midMW_none and midMW variants, which contain mismatches and wobble base pairs at different positions in pri-mir-342 (**a**) and pri-mir-106a (**b**). The midMW_none and midMW variants are shown in gray and blue, respectively. The *p*-values were calculated by two-sided Wilcoxon rank-sum tests. **c** Diagrams of pri-mir-342 and its variants. The red and green arrowheads indicate the CL-1 and CL0 cleavages of DROSHA, respectively. The red letters indicate mutated nucleotides. The capital letters represent pre-mir-342. **d**, **e** Processing of pri-mir-342 and its variants by D3-G2 (**d**) or D3-DG4 (**e**). For the short run gel results, each RNA (6 pmol) was incubated with D3-G2 (10 pmol) or D3-DG4 (6 pmol) for 120 min at 37 °C in 10 μl standard reaction buffer as described in the Methods section. The reaction mixtures were loaded onto 12% urea-PAGE, which was run for 40 min. For the long run gel results, each RNA (6 pmol) was incubated with D3-G2 (5 pmol) or D3-DG4 (3 pmol) for 60 min at 37 °C in 10 μl standard reaction buffer. The reaction mixtures were loaded onto 12% urea-PAGE, which was run for 75 min. **f**, **g** Bar graphs to show the relative alternative cleavage activity, which was estimated from experiments conducted in triplicate as a ratio of CL-1 to CL0 cleavage. The band densities of the F2 fragments resulting from the CL0 and CL1 cleavages were measured using Image Lab v.6.0.1. Data are presented as mean values +/− SEM. The asterisks (*) indicate statistical significant differences from the two-sided *t*-test (**f** midM_none vs. midM_89: *p* = 0.002, no_JC vs. midM_89: *p* = 0.004, midM_910 vs. midM_89: *p* = 0.002; **g** midM_none vs. midM_89: *p* = 2.9e−4, no_JC vs. midM_89: *p* = 0.002, midM_910 vs. midM_89: *p* = 0.001). **h** The F2 fragments resulting from the CL-1 and CL0 cleavage sites were cloned and sequenced by NGS. The size of the circle indicates the relative amount of each F2 fragment from the NGS data. **i** The expression of miR-342 in human cells, which were transfected with the plasmid expressing either pri-mir-342 or its variants, was estimated by miRNA sequencing. The size of the circle indicates the relative amount of each miRNA isomer from the miRNA sequencing data. The source data are provided in the Source Data file.

In high-throughput pri-miRNA processing assays, we also found that DROSHA cleaved more at alternative sites on the pri-mir-106a and pri-mir-342 variants that had mismatches and wobble base pairs at positions 8–9 (Fig. 3a, b). Indeed, the highest level of alternative cleavage occurred when there was a mismatch at position 8 (Fig. 3a, b); this was also the case for arti2 (Supplementary Fig. 2c–e). We hypothesized that these mismatches might play a role in the alternative cleavages of DROSHA for some human pri-miRNAs, including pri-mir-342. It is known that pri-mir-342 has two alternative cleavage sites, which are at CL-1 and CL0, and that it possesses a midM in the upper stem at positions 8–9 from CL0 (Fig. 3c). Consistent with previous reports^28,29^, in our pri-miRNA processing assays, when using either D3-G2 or D3-DG4, we identified two alternative cleavage sites for pri-mir-342, at CL-1 and CL0 (for D3-G2, Fig. 3d lanes 2 and 10; for D3-DG4, Fig. 3e lanes 2 and 10). In addition, removal of the midM (midM_none) coincided with the disappearance of the CL-1 cleavage site (for D3-G2, Fig. 3d lanes 4 and 12, Fig. 3f and Supplementary Fig. 3a; for D3-DG4, Fig. 3e lanes 4 and 12, Fig. 3g and Supplementary Fig. 3b). The F2 fragments of pri-mir-342 (midM_89, WT) and pri-mir-342 (midM_none) (Fig. 3d, e) were sequenced (Fig. 3h). In addition, shifting the midM to positions 9–10 in the upper stem significantly reduced the CL-1 cleavage (for D3-G2, Fig. 3d lanes 8 and 16, Fig. 3f and Supplementary Fig. 3a; for D3-DG4, Fig. 3e lanes 8 and 16, Fig. 3g and Supplementary Fig. 3b). However, substituting C with U in the 3p-basal RNA segment (no JC), which is known to weaken the basal junction, resulted in the enhanced cleavage by D3-G2 or D3-DG4 at CL-1 (for D3-G2, Fig. 3d lanes 6 and 14, Fig. 3f and Supplementary Fig. 3a; for D3-DG4, Fig. 3e lanes 6 and 14, Fig. 3g and Supplementary Fig. 3b). These data indicate that the midM and basal junction cooperate in guiding DROSHA to cleave pri-mir-342 at the two different locations. Thus, cleavage at CL-1 relies on the midM, whereas that at CL0 depends on the basal junction. We ectopically expressed pri-mir-342 (midM_89), pri-mir-342 (midM_none), and pri-mir-342 (midM_910) in HCT116 cells and carried out miRNA sequencing to identify the Microprocessor cleavage sites. Consistently, we found that pri-mir-342 (midM_89) produced more CL-1 miRNAs while pri-mir-342 (midM_none) and pri-mir-342 (midM_910) generated more CL0 miRNAs in human cells (Fig. 3i).

We selected pri-mir-200b, which has a canonical cleavage site at CL-1 and a midW (Supplementary Fig. 3c). We changed the wobble base pairs into Watson-Crick base pairs (midW_none) and found that Microprocessor generated the shorter pre-miRNA (Supplementary Fig. 3d). The F2 fragments of pri-mir-200b (midW_89, WT) and pri-mir-200b (midW_none) shown in Supplementary Fig. 3d, were sequenced (Supplementary Fig. 3e). The sequencing results indicate that removal of the midW resulted in the cleavage site changing from CL-1 to CL0, which is 8–9 nt from the midW (Supplementary Fig. 3d, compare lanes 6 and 8). This suggests that the midW is responsible for determining the CL-1 cleavage site of Microprocessor on pri-mir-200b. We also ectopically expressed pri-mir-200b (midW_89, WT) and pri-mir-200b (midW_none) in HCT116 cells and carried out miRNA sequencing for the transfected cells. Consistently, we found that pri-mir-200b (midW_89) produced more CL-1 miRNAs, whereas pri-mir-200b (midW_none) generated more CL0 miRNAs (Supplementary Fig. 3f).

### MidMW blocks the unproductive cleavage of DROSHA

**Fig. 4:**
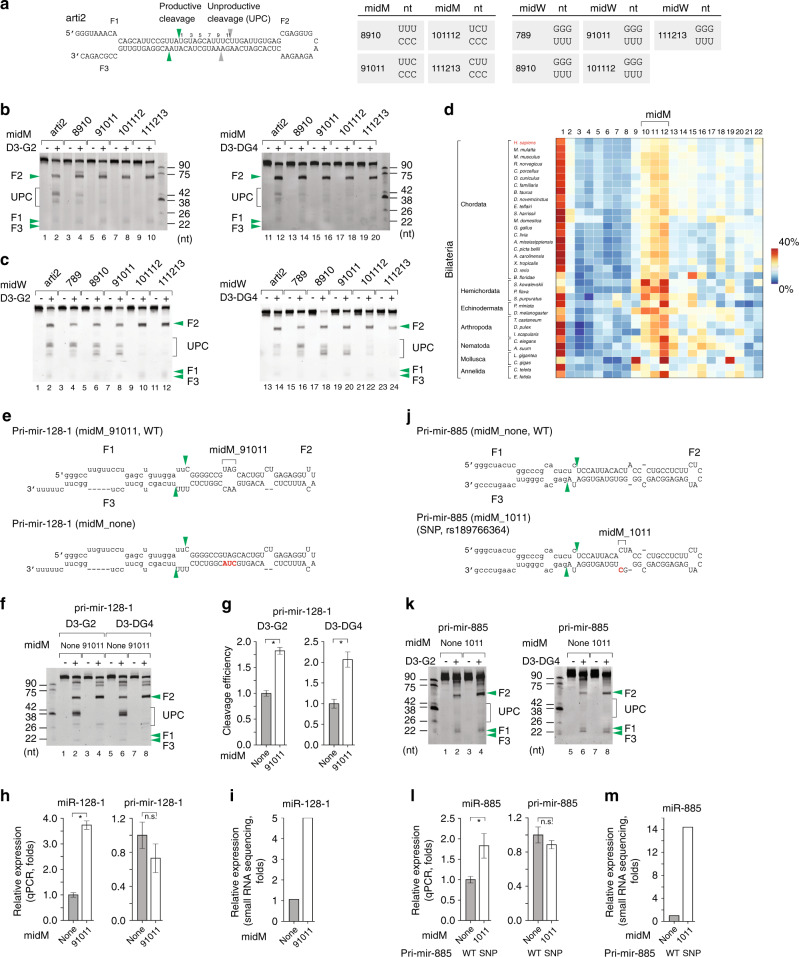
MidMW blocks the unproductive cleavage of DROSHA. **a** Diagram of arti2. The green and gray arrowheads indicate the productive and unproductive cleavage sites of DROSHA, respectively. The table shows the various arti2 variants with mismatches and wobble base pairs at different positions. **b**, **c** Processing of arti2 pri-miRNAs by D3-G2 or D3-DG4. The arti2 variants contain mismatches (**b**) and wobble base pairs (**c**) at different positions. Each RNA (6 pmol) was incubated with D3-G2 (10 pmol) or D3-DG4 (6 pmol) for 120 min at 37 °C in 10 μl standard reaction buffer as described in the Methods section. **d** The frequency of the unmatched nucleotides at different positions on 5p-strand in the upper stem of pri-miRNAs from different organisms, colored according to the color bar (right). **e**, **j** Diagrams of pri-mir-128-1 (**e**) and pri-mir-885 (**j**) and their variants. The green arrowheads indicate the canonical cleavage sites of DROSHA. The letters in red indicate mutated nucleotides. The capital letters indicate the pre-miRNA. **f**, **k** Processing of pri-mir-128-1 (**f**) and pri-mir-885 (**k**) by D3-G2 or D3-DG4. Each RNA (6 pmol) was incubated with D3-G2 (10 pmol) or D3-DG4 (6 pmol) for 120 min at 37 °C in 10 μl standard reaction buffer as described in the Methods section. **g** The estimated productive cleavage efficiency of D3-G2 or D3-DG4 on pri-mir-128-1 in **f** from experiments conducted in triplicate. The band densities were measured using Image Lab v.6.0.1. Data are presented as mean values +/− SEM. The asterisks (*) indicate statistical significant differences from the two-sided *t*-test (left graph midM_none vs. midM_91011: *p* = 0.005, right graph midM_none vs. midM_91011: *p* = 0.045). **h**, **l** Bar graphs showing the expression of miR-128-1 (**h**) and miR-885 (**l**) quantified by qPCR from the ectopically expressed pri-mir-128-1 and pri-mir-885, respectively. The plasmids expressing WT and mutant pri-miRNA were transfected into HCT116 cells along with the plasmid expressing pri-mir-16-1. The expression levels of miRNA and pri-miRNA were normalized against those of miR-16-1 and pri-mir-16-1, respectively. Data are presented as mean values +/− SEM, *n* = 3. The asterisks (*) and n.s. indicate statistical significant differences and no statistical significant differences, respectively, from the two-sided *t*-test (**h** left graph midM_none vs. midM_91011: *p* = 1.2e−4, right graph midM_none vs. midM_91011: *p* = 0.306; **l** left graph midM_none vs. midM_1011: *p* = 0.050, right graph midM_none vs. midM_1011: *p* = 0.342). **i**, **m** Bar graphs showing the ectopic expression of miR-128-1 (**i**) and miR-885 (**m**) quantified by miRNA sequencing from the ectopically expressed pri-mir-128-1 and pri-mir-885, respectively. UPC: unproductive cleavage. The source data are provided in the Source Data file.

We examined the effect of mismatches in the upper stem in further detail and found that those at positions 9–11, 10–12, and 11–13 nearly completely blocked the unproductive cleavages of DROSHA (Fig. 4a, b). Because positions 10–12 in the upper stem are also regarded as being positions −3 to −1 relative to the unproductive cleavage sites when counting from the apical junction (Supplementary Fig. 4a), we hypothesized that mismatches at positions −3 to −1 (which were 3 to 1 nt upstream of the cleavage sites) might be inhibitory to the productive cleavage activity of DROSHA. We showed that this was indeed the case (Supplementary Fig. 4a, b, lanes 4, 6, and 8), and demonstrated that triple and double mismatches had a stronger inhibitory effect than a single mismatch within the −3 to −1 region (Supplementary Fig. 4c, d). Consistent with this result in the −3 to −1 region, triple and double mismatches at positions 10–12 had a stronger inhibitory effect on the unproductive cleavage of D3-G2 and D3-DG4 than a single mismatch (Supplementary Fig. 4e). In addition, we found that a midW at positions 10–12 and 11–13 blocked unproductive cleavages similar to a midM (Fig. 4c), and that triple wobble base pairs had a stronger effect than double or single wobble base pair (Supplementary Fig. 4f).

We then counted the number of unmatched nucleotides in the upper stem of 588 human pri-miRNAs listed in MirGeneDB v2.0^30^. The position of each unmatched nucleotide was counted from the first nucleotide of the mature 5p miRNA, which also indicates the cleavage sites of DROSHA. We found that 48.30% (i.e., 284/588) of these human pri-miRNAs contain at least one unmatched nucleotide within positions 10–12, whereas 10.88% contain 2 or 3 unmatched nucleotides in the same region. The unmatched nucleotides were also enriched at positions 10–12, when compared with other regions in the upper stem of human pri-miRNAs (Fig. 4d). Interestingly, the presence of unmatched nucleotides at positions 10–12 also appears to be a common feature of pri-miRNAs in various other organisms (Fig. 4d).

We selected pri-mir-128-1, which has a midM at positions 9–11 to test the effect of the midM (Fig. 4e). Removal of the midM (midM_none) reduced the productive cleavage activity (by 1.5 folds) but increased the unproductive cleavage activity of both D3-G2 (Fig. 4f, compare lanes 2 and 4, and Fig. 4g) and D3-DG4 (Fig. 4f, compare lanes 6 and 8, and Fig. 4g). We also ectopically expressed pri-mir-128-1 and pri-mir-128-1 (midM_none) in HCT116 cells and carried out qPCR and miRNA sequencing for the transfected cells. Consistently, we found that pri-mir-128-1 with the midM produced more miRNAs than pri-mir-128-1 without the midM (midM_none) (Fig. 4h, i).

We hypothesized that the midM might be a target of the miRNA expression regulatory mechanism in human cells. Thus, we selected pri-mir-885, which contains a SNP in a midM at positions 10–11 (Fig. 4j). Interestingly, we found that this SNP stimulated D3-G2 and D3-DG4 productive cleavage activity in vitro (for D3-G2, Fig. 4k, compare lanes 2 and 4, for D3-DG4, Fig. 4k, compare lanes 6 and 8), as well as miRNA expression in human cells (Fig. 4l, m).

### SeedM inhibits the productive cleavage of DROSHA

**Fig. 5:**
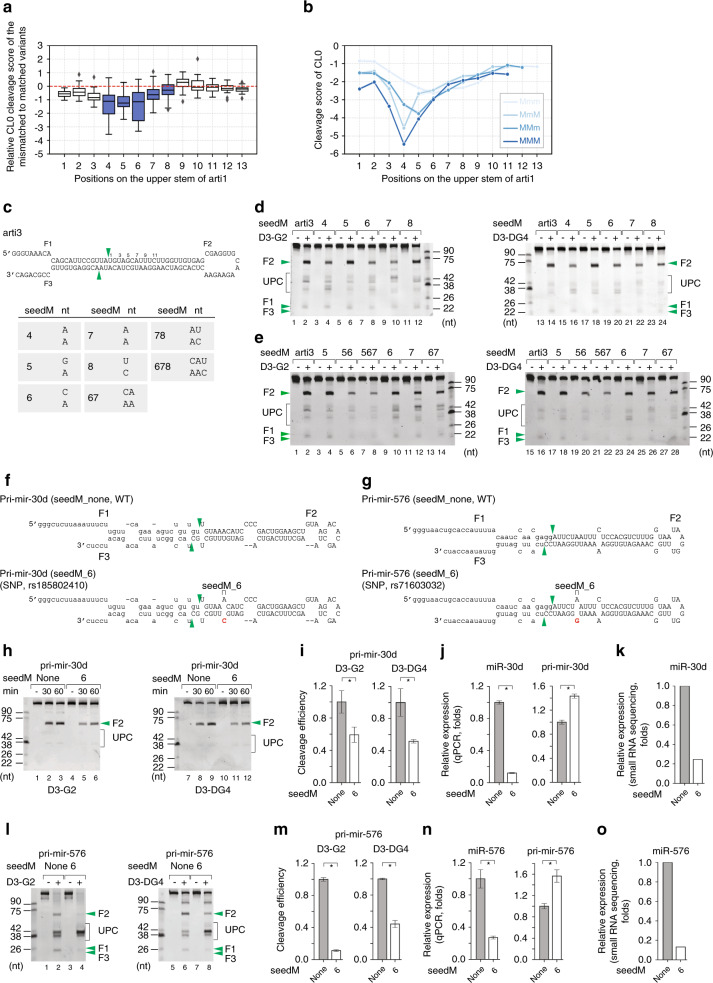
SeedM inhibits the productive cleavage of DROSHA. **a** The productive cleavage efficiency of DROSHA in the high-throughput processing assays. The CL0 cleavage score was estimated for each variant. The relative cleavage score of each variant containing a mismatch at a particular position was normalized against that of the variants containing a Watson-Crick base pair at the same position. **b** The cleavage score of variants containing 1, 2, or 3 mismatches at different positions. Mmm: single mismatch; MmM: double mismatches separated by a matched base pair; MMm: consecutive double mismatches; MMM: consecutive triple mismatches. **c** Diagram showing the various arti3 variants with mismatches at different positions. The green arrowheads indicate the productive cleavage sites of DROSHA at 13 nt from the basal junction. **d**, **e** Processing of arti3 by D3-G2 or D3-DG4. The arti3 variants contain the mismatches at different positions. Each RNA (6 pmol) was incubated with D3-G2 (10 pmol) or D3-DG4 (6 pmol) for 120 min at 37 °C in 10 μl standard reaction buffer as described in the Methods section. **f**, **g** Diagrams of pri-mir-30d in **f** or pri-mir-576 in **g** and their variants. The green arrowheads indicate the canonical cleavage sites of DROSHA. The letters in red indicate mutated nucleotides. The capital letters indicate pre-mir-30d and pre-mir-576. **h**, **l** Processing of pri-mir-30d in **h** or pri-mir-576 in **l** and their variants by D3-G2 or D3-DG4. Each substrate was incubated with D3-G2 (5 pmol) or of D3-DG4 (3 pmol) for 60 min at 37°C in 10 μl standard reaction buffer as described in the Methods section. **i**, **m** The estimated productive cleavage efficiency of D3-G2 or D3-DG4 on pri-mir-30d in **i** or pri-mir-576 in **m** and their variants from experiments conducted in triplicate. Data are presented as mean values +/− SEM. The asterisks (*) indicate statistical significant differences from the two-sided *t*-test (**i** left graph seedM_none vs. seedM_6: *p* = 0.071, right graph seedM_none vs. seedM_6: *p* = 0.049; **m** left graph seedM_none vs. seedM_6: *p* = 3.8e−6, seedM_none vs. seedM_6: *p* = 2.3e−4). **j**, **n** Bar graphs showing the expression of miR-30d and miR-576 quantified by qPCR from the ectopically expressed pri-mir-30d (**j**) and pri-mir-576 (**n**), respectively. The plasmids expressing WT and mutant pri-mir-30d and pri-mir-576 were transfected into HCT116 and HEK293T cells, respectively, along with the plasmid expressing pri-mir-16-1. The expression levels of miRNA and pri-miRNA were normalized against those of miR-16-1 and pri-mir-16-1, respectively. Data are presented as mean values +/− SEM. The asterisks (*) statistical significant differences from the two-sided *t*-test (**j**) left graph seedM_none vs. seedM_6: *p* = 2.2e−6, right graph seedM_none vs. seedM_6: *p* = 8.7e−4; **n** seedM_none vs. seedM_6: *p* = 0.003, right graph seedM_none vs. seedM_6: *p* = 0.011). **k**, **o** Bar graphs showing the ectopic expression of miR-30d (**k**) and miR-576 (**o**) from the ectopically expressed pri-mir-30d and pri-mir-576, respectively. UPC: unproductive cleavage. The source data are provided in the Source Data file.

We examined the effects of having mismatches and wobble base pairs at different positions on the efficiency of the productive cleavage activity of DROSHA (i.e., at CL0). Thus, we estimated the cleavage score of DROSHA at CL0 for each variant of arti1. The CL0 cleavage score was calculated as a ratio of the CL0 product to the original substrate. First, we compared the averaged CL0 cleavage score of the variants containing a mismatch within positions 1–13, with that of their corresponding matched variants. The results showed that a mismatch within positions 4–8 significantly reduced the activity of DROSHA (Fig. 5a). Further analysis showed that double and triple mismatches within the 4–8 region further decreased the enzymatic activity of DROSHA (Fig. 5b).

To validate this observation, we generated arti3 variants, which contained single, double, or triple mismatches within the 4–8 region (Fig. 5c and Supplementary Fig. 5a). The arti3 sequence was as described in previous studies^13,14^, and the variants were tested using the D3-G2 and D3-DG4 complexes. We found that a mismatch at position 4, 6 or 7 largely reduced the cleavage ability of D3-G2 and D3-DG4 (Fig. 5d, lanes 4, 8, 10, 16, 20, and 22), whereas a mismatch at position 5 or 8 had a less profound effect (Fig. 5d, lanes 6, 12, 18, and 24). A combination of two mismatches (i.e., 56 and 67 in Fig. 5e; 46 and 56 in Supplementary Fig. 5b; and 67 and 78 in Supplementary Fig. 5c) further decreased the enzymatic activity of DROSHA, when compared with any single mismatch variant. Furthermore, triple mismatches (i.e., 567 in Fig. 5e; 456 in Supplementary Fig. 5b; and 678 in Supplementary Fig. 5c) nearly almost completely abolished DROSHA the cleavage activity of DROSHA. We have termed the mismatches in this region as seedM because they are located in the seed region of 5p miRNAs, i.e., at positions 2–8 from the DROSHA cleavage site.

We demonstrated the inhibitory effects of seedM on pri-mir-16-1 and pri-mir-1277 (Supplementary Fig. 5d–g). Furthermore, we selected two human pri-miRNAs (i.e., pri-mir-30d and pri-mir-576) that have an SNP, which generates a seedM in position 6 (Fig. 5f, g)^31^. The pri-miRNA processing results for these pri-miRNAs showed that the seedM reduced the cleavage activity of D3-G2 and D3-DG4 for both of the SNP-containing pri-miRNAs (for pri-mir-30d, Fig. 5f, h and i; for pri-mir-576, Fig. 5g, l and m). We also showed by both qPCR and miRNA sequencing that the cellular miRNA expression from these SNP-containing pri-miRNAs was reduced (for pri-mir-30d, Fig. 5j, k; for pri-mir-576, Fig. 5n, o).

### SeedW inhibits the productive cleavage of DROSHA

**Fig. 6:**
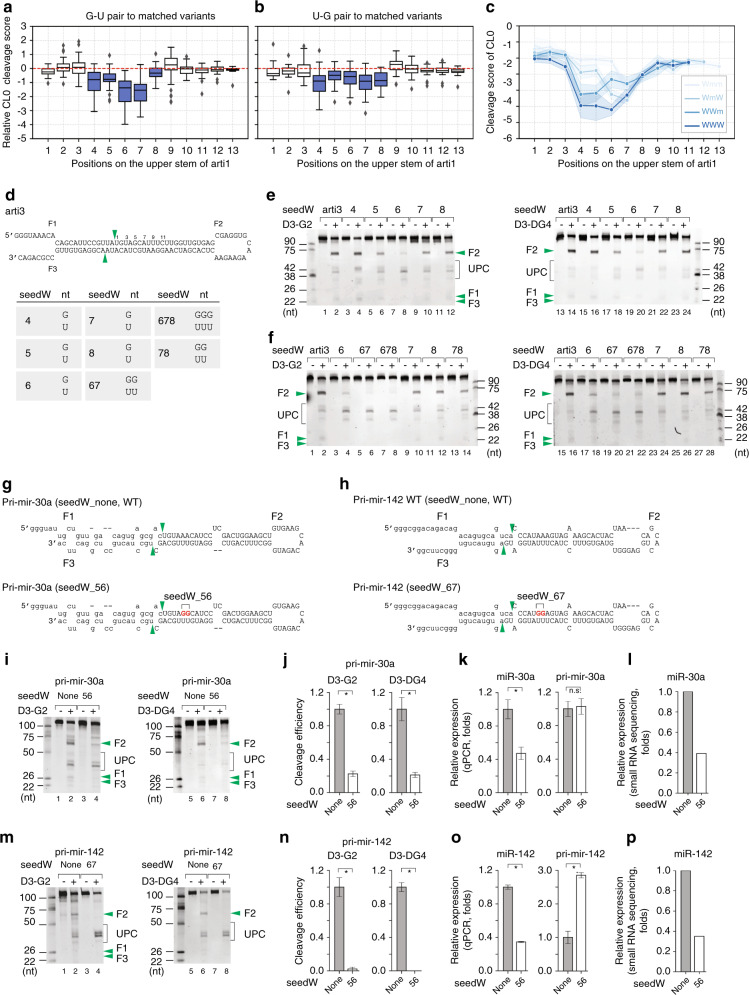
SeedW inhibits the productive cleavage of DROSHA. **a**, **b** The productive cleavage efficiency of DROSHA in the high-throughput processing assays. The CL0 cleavage score was estimated for each variant. The relative cleavage score of each variant containing a G–U pair in (**a**) or U–G pair in (**b**) at a position was normalized against that of the variants containing a Watson-Crick base pair at the same position. **c** The cleavage score of variants containing 1, 2, or 3 G–U pairs at different positions. Wmm: single wobble base pair; WmW: double wobble base pairs separated by a Watson-Crick base pair; WWm: consecutive double wobble base pairs; WWW: consecutive triple wobble base pairs. **d** Diagram showing the arti3 variants. The green arrowheads indicate the productive cleavage sites of DROSHA. The table shows the wobble base pairs at different positions. **e**, **f** Processing of arti3 by D3-G2 or D3-DG4. Each RNA (6 pmol) was incubated with D3-G2 (10 pmol) or D3-DG4 (6 pmol) for 120 min at 37 °C in 10 μl standard reaction buffer as described in the Methods section. **g**, **h** Diagrams of pri-mir-30a in **g** or pri-mir-142 in **h** and their variants. The green arrowheads indicate the productive cleavages of DROSHA at 13 nt from the basal junction. The letters in red indicate mutated nucleotides. The capital letters indicate pre-mir-30a and pre-mir-142. **i**, **m** Processing of pri-mir-30a in **i** or pri-mir-142 in **m** and their variants by D3-G2 or D3-DG4. Each RNA was added with D3-G2 (10 pmol) or D3-DG4 (6 pmol). Pri-mir-30a and pri-mir-142 substrates were incubated at 37 °C in 10 μl standard reaction buffer for 120 and 60 min, respectively. **j**, **n** The estimated productive cleavage efficiency of D3-G2 or D3-DG4 on pri-mir-30a in (**j**) or pri-mir-142 in (**n**) and their variants from experiments conducted in triplicate. Data are presented as mean values +/− SEM. The asterisks (*) statistical significant differences from the two-sided *t*-test (**j** left graph seedW_none vs. seedW_56: *p* = 0.003, right graph seedW_none vs. seedW_56: *p* = 0.005; **n** left graph seedW_none vs. seedW_67: p = 0.002, seedW_none vs. seedW_67: *p* = 0.008). **k**, **o** Bar graphs showing the ectopic expression of miR-30a from pri-mir-30a in **k** or miR-142 from pri-mir-142 in **o**. The plasmids expressing WT and mutant pri-miRNA were transfected into HEK293T cells along with the plasmid expressing pri-mir-16-1. The expression levels of miRNA and pri-miRNA were normalized against those of miR-16-1 and pri-mir-16-1, respectively. Data are presented as mean values +/− SEM, *n* = 3. The asterisks (*) and n.s. indicate statistical significant differences and no statistical significant differences, respectively, from the two-sided *t*-test (**k** left graph seedW_none vs. seedW_56: *p* = 0.018, right graph seedW_none vs. seedW_56: *p* = 0.838; **o** left graph seedW_none vs. seedW_67: *p* = 1.6e−6, right graph seedW_none vs. seedW_67: *p* = 7.6e−4). **l**, **p** Bar graphs showing the ectopic expression of miR-30a (**l**) and miR-142 (**p**) quantified by miRNA sequencing from the ectopically expressed pri-mir-30a and pri-mir-142, respectively. UPC: unproductive cleavage. The source data are provided in the Source Data file.

We compared the averaged CL0 cleavage score of each of the variants containing a wobble base pair within positions 1–13, with that of its corresponding matched Watson-Crick base pair variant. Interestingly, the G-U wobble base pair variants showed weaker activity than the Watson-Crick base pair variants at positions 4–8 (Fig. 6a, b). In addition, we found that the G-U pair appeared to reduce the activity of DROSHA more significantly than the U-G pair (Fig. 6a, b). We also observed that double or triple G-U pairs at positions 4–8 further reduced the cleavage activity of DROSHA (Fig. 6c). The effects of the mismatches and wobble base pairs at positions 4–8 were consistently seen with the three high throughput pri-miRNA processing assays conducted on pri-mir-106a, pri-mir-514a-1, and pri-mir-342 (Supplementary Fig. 6a–c).

To validate these observations, we generated arti3 variants that contain single, double, or triple G-U pairs within the 4–8 region (Fig. 6d and Supplementary Fig. 6d). We called the wobble base pairs within this region a seedW. As shown in Fig. 6e, a single G–U in positions 5–7 largely reduced the cleavage activity of DROSHA, whereas a G-U at position 4 or 8 had a less compelling effect. However, double or triple G-U pairs within positions 4–8 almost entirely abolished the cleavage activity of DROSHA (i.e., 67, 78, and 678 in Fig. 6f; 46, 56, and 456 in Supplementary Fig. 6e; 56, 67, and 567 in Supplementary Fig. 6f). Furthermore, G-U pairs had a stronger effect in reducing the activity of DROSHA than did U-G pairs at similar positions on arti3 (Supplementary Fig. 6g, compare lanes 4 and 6, 8 and 10, 12 and 14, 18 and 20, 22 and 24, and 26 and 28). Because mismatches and wobble base pairs at positions 4–8 decreased DROSHA activity, we examined the effect of combining mismatches and wobble base pairs within this region in further detail. As shown in Supplementary Fig. 6h, a mismatch, and a wobble base pair together decreased the activity of DROSHA more than either a mismatch or a wobble base pair alone.

We selected pri-mir-30a, which has AA-UU at positions 5–6, and we mutated AA-UU into GG-UU (Fig. 6g). We then conducted the pri-miRNA processing assay with both WT and mutant pri-miRNAs using D3-G2 and D3-DG4. As shown in Fig. 6i, j, the seedW inhibited the activity of DROSHA and thus prevented its cleavage of the seedW-containing pri-mir-30a at the productive site. As a consequence, DROSHA produced less pre-mir-30a (F2). We also observed a similar effect of seedW on pri-let-7d, in which we mutated the GU-CG in positions 5-6 into AA-UU and GG-UU (Supplementary Fig. 6i). The results in Supplementary Fig. 6j, k show that the presence of seedW inhibited the productive cleavage activity of both D3-G2 and D3-DG4. We also expressed pri-mir-30a or the pri-mir-30a variant (seedW_56) in human cells and examined the expression of miRNA by qPCR and miRNA sequencing. Consistent with our in vitro data, we observed that seedW-containing pri-miRNAs produced a lower amount of miRNA than pri-mir-30a (Fig. 6k, l). These data indicate that the seedW also reduced the miRNA expression in human cells.

It is known that the hematopoietic-specific pri-miRNA, pri-mir-142, can be edited by ADAR enzymes, and this leads to a reduction of miR-142 biogenesis^32^. While this ADAR-induced modification is understood to inhibit the enzymatic activity of Microprocessor, the mechanism is elusive. It is reported that ADAR enzymes convert several A-U pairs in pri-mir-142 to I-U wobble base pairs by converting A into I, and they are especially active at positions 6–7 of pri-mir-142^32^. Therefore, we generated a wobble I-U mimicking pri-mir-142 by mutating A-U to G-U at positions 6–7 (Fig. 6h). We found that this seedW inhibited both D3-G2 and D3-DG4 activity (Fig. 6m, n). We also confirmed the effect of the seedW in human cells using both qPCR and miRNA sequencing (Fig. 6o, p). This indicates that ADAR-edited pri-miRNAs block pri-miRNA processing by inhibiting DROSHA activity using the seedW mechanism.

### Coordination of midMW and seedMW with UG, UGU, and mGHG motifs

**Fig. 7:**
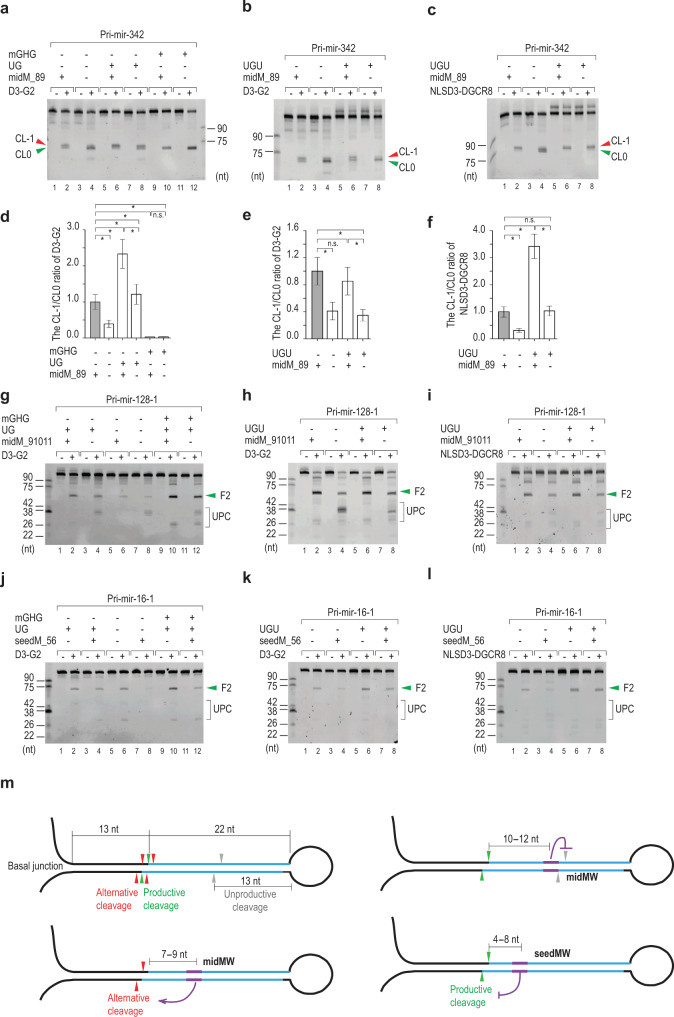
Coordination of midMW and seedMW with UG, UGU, and mGHG in determining the accuracy and efficiency of Microprocessor cleavages. **a**–**c** Processing of pri-mir-342 variants by D3-G2 or NLSD3-DGCR8. Each RNA (6 pmol) was incubated with D3-G2 (5 pmol) or NLSD3-DGCR8 (3 pmol) for 60 min at 37 °C in 10 μl standard reaction buffer. **d**–**f** Bar graphs to show the relative alternative cleavage activity, which was estimated from experiments conducted in triplicate as a ratio of CL-1 to CL0 cleavage. The band densities of the F2 fragments resulting from the CL-1, and CL0 cleavages were measured using Image Lab v.6.0.1. Data are presented as mean values +/− SEM. The asterisks (*) and n.s. indicate statistical significant differences and no statistical significant differences, respectively, from the two-sided *t*-test (**d** midM_none vs. midM_89: *p* = 0.002, midM_89_UG vs. midM_89: *p* = 1.1e-5, midM_none_UG vs. midM_89: *p* = 0.006, midM_89_GHG vs. midM_89: *p* = 0.0013, midM_none_GHG vs. midM_89: *p* = 0.046, midM_89_UG vs. midM_none_UG: *p* = 0.001, midM_89_GHG vs. midM_none_GHG: *p* = 0.923; **e** midM_none vs. midM_89: *p* = 0.009, midM_89_UGUG vs. midM_89: *p* = 0.064, midM_none_UGUG vs. midM_89: *p* = 0.011, midM_89_UGUG vs. midM_none_UGUG: *p* = 0.005; **f** midM_none vs. midM_89: *p* = 1.7e-4, midM89_UGUG vs. midM_89: *p* = 1.5e-5, midM_none_UGUG vs. midM_89: *p* = 0.472, midM_89_UGUG vs. midM_none_UGUG: *p* = 2.3e-4). **g**–**l** Processing of pri-mir-128-1 (**g**–**i**) and pri-mir-16-1 (**j**–**l**) variants by D3-G2 or NLSD3-DGCR8. Each RNA was added with D3-G2 (10 pmol) or NLSD3-DGCR8 (6 pmol). Pri-mir-128-1 and pri-mir-16-1 substrates were incubated at 37 °C in 10 μl standard reaction buffer for 120 and 30 min, respectively. **m** Mismatched and wobble base pairs in the upper stem of pri-miRNA govern the accuracy and efficiency of Microprocessor. The midMW that contains mismatches and/or wobble base pairs in the middle of the miRNA sequence stimulates the alternative cleavages and/or blocks the unproductive cleavages of Microprocessor when located at 7–9 nt and 10–12 nt in the upper stem, respectively. The seedMW that possesses mismatches and/or wobble base pairs in the seed region of the 5p miRNA sequence inhibits the productive cleavages of Microprocessor. The green, red, and gray arrowheads indicate the productive, alternative, and unproductive cleavages, respectively. The blue lines show the miRNA sequence embedded in pri-miRNA. UPC: unproductive cleavage. The source data are provided in the Source Data file.

We investigated the coordination of midMW_89 with the UG, mGHG, and UGU motifs in determining the cleavage sites of Microprocessor using pri-mir-342 substrates. The combination midMW and UG increased the CL-1 cleavage more than midMW or UG alone (Fig. 7a, compare lanes 6 with 2 and 8; Fig. 7d; and Supplementary Fig. 7a), suggesting that the midMW and UG motifs cooperate in determining the CL-1 cleavage. The addition of the mGHG motif at the −5 position from CL0 enhanced the CL0 cleavage of Microprocessor (Fig. 7a, lane 12; Fig. 7d; and Supplementary Fig. 7a), but the addition of midMW_89 to the mGHG motif weakened the CL0 cleavage (Fig. 7a, compare lanes 10 and 12; Fig. 7d; and Supplementary Fig. 7a). This indicates that the mGHG motif alone might play a dominant role in the determination of CL0 cleavage, and when mGHG was combined with midMW then CL0 cleavage was reduced. Since DGCR8 interacts with the UGU motif, we added the UGU motif in the loop of pri-mir-342 and investigated the coordination of the midMW and the UGU motifs. The combination of midMW and UGU did not show any effect on the cleavage patterns of DROSHA (D3-G2; Fig. 7b, Fig. 7e, and Supplementary Fig. 7a). However, this combination further increased the CL-1 cleavage of Microprocessor (NLSD3-DGCR8) compared with midMW or UGU alone (Fig. 7c, compare lanes 6 and 2 or 8; Fig. 7f; and Supplementary Fig. 7a). This suggests that the CL-1 cleavage was maximized via two coordinated actions: midMW acting on DROSHA and UGU functioning on DGCR8.

Next, we investigated the collaboration of midMW10–12 and each of the UG, mGHG, or UGU motifs in reducing the unproductive cleavages of Microprocessor using pri-mir-128-1 and pri-mir-576 substrates (Supplementary Fig. 7b, d). We demonstrated that midMW10–12 reduced the unproductive cleavage of Microprocessor on these 2 pri-miRNA backbones regardless of the presence or absence of the UG, mGHG, and UGU motifs (Fig. 7g–i and Supplementary Fig. 7d–f). Therefore, midMW10–12 complies with each of these motifs to enhance the productive cleavage of Microprocessor.

Finally, we examined the inhibitory effects of seedMW on the productive cleavage of Microprocessor in combination with each of UG, mGHG, or UGU motifs using pri-mir-16-1 and pri-mir-30d substrates (Supplementary Fig. 7c, g). We found that seedMW reduced the productive cleavage of Microprocessor on these 2 pri-miRNA backbones regardless of the presence or absence of the three motifs (Fig. 7j–l and Supplementary Fig. 7g–i). Therefore, seedMW and each of the motifs oppositely influenced the productive cleavage of Microprocessor.

## Discussion

In this study, we have revealed the important roles of mismatches and wobble base pairs located in the upper stem of pri-miRNAs, on pri-miRNA processing by Microprocessor. As these mismatches and wobble base pairs were found in the middle and seed region of 5p miRNA sequences, we called them midM or midW (midMW) and seedM or seedW (seedMW), respectively. MidMW located at 7–9 nt and 10–12 nt in the upper stem affected alternative cleavages and blocked the unproductive cleavage of DROSHA, respectively (Fig. 7m). In addition, a seedMW located at 4–8 nt in the upper stem inhibited the productive cleavage (Fig. 7m). It has previously been shown that bulges introduced in a similar region in several human pri-miRNAs also reduced miRNA expression in human cells^19^. Together, these findings suggest that perturbing the RNA duplex structure of the seedMW region negatively affects pri-miRNA processing.

Here, high-throughput processing assays were conducted using a minimal Microprocessor complex (D3TN1-G2 or D3-G2), where the G2 fragment of DGCR8 does not contribute to the RNA binding affinity^13^, so that we could detect the effects of the revealed RNA elements to D3TN1 or DROSHA. We also conducted experiments with many different pri-miRNAs, and together our data indicate that midMW and seedMW govern Microprocessor activity through DROSHA. In addition, it is known that the basal junction of pri-miRNAs and the basal UG motif, which are ~14–13 upstream of the cleavage sites, are both critical for DROSHA to interact and cleave pri-miRNAs at the basal sites^13,14,17^. Here, we showed that a midMW located ~10–12 nt downstream of the cleavage sites or 23–26 nt from the basal junction affects DROSHA activity. This suggests that DROSHA might interact with the long dsRNA region of ~23–25 bp ranging from the basal junction to the midMW. Our data also support the proposed structure of DROSHA, which suggests that it has an elongated shape^14^. We also showed that DROSHA without DGCR8 mainly cleaves pri-miRNA at 13 nt from the basal junction. However, to some extent, it might also cleave pri-miRNAs at multiple positions near the junction on the different stem structures. Although these cleavages were completely blocked by the addition of DGCR8 in our tested pri-miRNAs, it is possible that DGCR8 might coordinate with DROSHA in some other stem-loop RNA structures, facilitating these near junction cleavages.

The cleavage sites of Microprocessor at the basal junction are determined by several RNA elements, including the stem length and the mGHG motif in the lower stem^18,22,25^. For example, the splicing factor, SRSF3, recruits DROSHA to the basal junction in a manner dependent on the major conserved RNA motif, CNNC^27^. Here, we identified another mechanism that appears to control the site of DROSHA cleavage at the basal junction. We showed that midMW7–9 affects the site of DROSHA cleavage. While seedMW and midMW10–12 seem to affect the cleavage efficiency of Microprocessor on a variety of pri-miRNA backbones, the influence of midMW7–9 on the cleavage site choices of Microprocessor is likely RNA-context dependent. Indeed, we demonstrated that midMW7–9 cooperates with the known UG, UGU, and mGHG motif (Fig. 7a–f) in determining the cleavage sites. It would be of interest now, to investigate how midMW7–9 coordinates with these known RNA motifs and even unknown RNA elements to modify the cleavage sites of Microprocessor. In addition, the midMW10–12 enhances the cleavage efficiency of Microprocessor by blocking unproductive cleavages. Interestingly, this midMW10–12 seems to be enriched in the pri-miRNAs of various organisms, not just in humans, suggesting that its function might be conserved throughout evolution.

In previous studies, most of the RNA elements identified, including the basal junction, basal UG motif, lower stem mGHG, apical UGU motif, and apical loop size were demonstrated function as pri-miRNA processing enhancers and stimulate the cleavage activity of DROSHA^13,14,16–18,21,22,33,34^. Here, we reveal that seedMW functions to inhibit pri-miRNA processing. Unlike the RNA elements mentioned above, seedMW is not enriched in human pri-miRNAs, but we suggest that it might serve as an essential site for the regulation of miRNA expression. Here, we also demonstrated that several examples of RNA-editing events and SNPs occurring on the DNA region coding for the seedMW alter pri-miRNA processing. Interestingly, the G-U base pair in the seedMW has a stronger inhibitory effect than the mismatch or U-G base pair, suggesting that DROSHA might have a mechanism to distinguish between G-U and U-G base pairs in seedMW. In a previous study, we demonstrated that the internal loop in the lower stem of pri-miRNAs selectively inhibits Microprocessor from cleaving the 3p-strand of pri-miRNAs, thereby enhancing its cleavage efficiency of the 5p-strand^35^. It would, therefore, be of interest to investigate if seedMW affects the ability of Microprocessor to cleave both strands equally or just one of the strands.

Various SNPs and ADAR-editing events take place on the upper stem of pri-miRNAs^31,32,36–39^. In addition, miRNA-encoding genes might be mutated during the development of cancer^40^. An increase in the incidence of SNPs, ADAR-editing, and/or gene mutations might lead to the appearance or disappearance of mismatches and wobble base pairs in the upper stem of pri-miRNAs. Many of these events are associated with human diseases^31,32,36–40^. The findings of this study, therefore, provide a foundation for understanding how various SNPs, ADAR-editing, and gene mutation events, which affect midMW or seedMW, might change pri-miRNA processing and in turn, the expression and function of miRNAs.

## Methods

### Purification of recombinant proteins

The D3 fragment (amino acids 390–1365) of DROSHA was fused to the C-terminal protein G and 10×His tag in pXab-D3 used in the previous study^13^. The pXab-D3TN1 was generated from pXab-D3 by replacing the glutamate residue at position 1045 with a glutamine residue. The G2 (amino acids 701–773) and DG4 fragments (amino acids 285–750) of DGCR8 were fused to the C-terminal GFP and 10×His tag in the pXG plasmid^13^ to generate pXG-G2 and pXG-DG4, respectively (Supplementary Table 1). The pXab-D3 and pXG plasmids were gifts from Dr. Narry Kim (Seoul National University, Korea).

In order to express the D3-G2 or D3TN1-G2 complex, pXab-D3 or pXab-D3TN1 was co-transfected with pXG-G2 into 120 100-mm dishes of HEK293E cells (also kindly provided by Dr. Narry Kim). The transfected cells were harvested 3 days after transfection. The cell pellets were resuspended in T500 buffer (20 mM Tris-HCl [pH 7.5], 500 mM NaCl) plus 4 mM β-mercaptoethanol (Sigma-Aldrich), 2 μg/ml RNase A (Thermo Scientific), and protease inhibitor cocktail (Thermo Scientific). The clear lysate obtained by sonication and centrifugation was loaded onto an Ni-NTA resin (Bio-Rad). After loading, the resin was washed sequentially with T1000 (20 mM Tris-HCl [pH 7.5], 1000 mM NaCl) plus 4 mM β-mercaptoethanol, T0 (20 mM Tris-HCl [pH 7.5], 0 mM NaCl) plus 4 mM β-mercaptoethanol, and T100 (20 mM Tris-HCl [pH 7.5], 100 mM NaCl) plus 4 mM β-mercaptoethanol and 20 mM imidazole (Sigma-Aldrich), after which the recombinant proteins were eluted with T100 containing 200 mM imidazole. The peak fractions containing the recombinant proteins were loaded onto a Unosphere Q ion exchange resin (Bio-Rad), after which the resin was washed sequentially with T100 and T150, and then eluted with T500 plus 10% glycerol and 2 mM dithiothreitol (DTT) (Sigma-Aldrich). The peak fractions from this column were loaded onto a gel filtration column (Superdex 200 increase 10/300 GL column, GE Healthcare), which was run using standard NGC chromatography systems (Bio-Rad). The peak fractions from the gel filtration were aliquoted and stored at −80 °C until required.

To express the D3-DG4 complex, pXab-D3 was co-transfected with pXG-DG4 into 100 100-mm dishes of HEK293E cells. The transfected cells were harvested after 4 days of transfection, and the purification procedure was similar to that described for the D3-G2 and D3TN1-G2 complexes.

To express the NLSD3-DGCR8 complex, we transfected pXab-NLSD3 and pXG-DGCR8 in 70 100-mm dishes of HEK293E cells and purified the complex using the same methodology described above for the D3-G2 and D3TN1-G2 complexes. The NLS sequence, which codes for 7 amino acids (i.e., PKKKRKV) was added to the D3-coding sequence in the pXab-D3 vector.

### High-throughput arti1 processing assays

Eleven single-stranded DNA (ssDNA) oligos, which contain random nucleotide sequences in 3-nt windows (as shown in Fig. 1c) were synthesized by Integrated DNA Technologies (IDT). The ssDNA sequences are shown in Supplementary Table 2. We used the F-T7-arti1 and RTP-RA3-arti1 primers (Supplementary Table 2) in the PCR reaction to amplify the double-stranded DNA (dsDNA) from each ssDNA; thus, we obtained 11 groups of T7 promoter-containing dsDNAs. Two hundred nanogram of each dsDNA group was used in a 20 µl in vitro transcription (IVT) reaction using the MEGAscript T7 transcription kit (Invitrogen). The IVT-synthesized RNAs (arti1) were gel-purified and quantified with NanoDrop 2000 spectrophotometer (Thermo Scientific). We collected 11 groups of arti1 with different randomized sequences. Approximately 30 µg of RNA of each group was obtained and separately stored at −80 °C until required.

In the high-throughput pri-miRNA processing assays, 6 pmol of arti1 from each group was incubated with 10 pmol of the D3TN1-G2 complex in 10 µl standard reaction buffer containing 50 mM Tris-HCl (pH 7.5), 150 mM NaCl, 10% glycerol (Sigma-Aldrich), 0.2 μg/μl BSA, 1 mM DTT and 2 mM MgCl_2_. An equal amount of the same RNA was mixed with T500 plus 2 mM DTT and 10% glycerol as a control sample. The processing reaction was carried out at 37 °C for 120 min, after which the control and reaction samples of each group were separated by 10% urea-PAGE. The original substrate from the control samples, the uncleaved substrate and the cleaved product (i.e., the F2-3 fragment) from each RNA group were separately cut from the gel and purified (Fig. 1e). Subsequently, we pooled all the control, uncleaved substrates, or cleaved products from the 11 different processing reactions. We then reverse transcribed the 3 pooled RNAs using SuperScript™ IV Reverse Transcriptase (Invitrogen) with the cirRTP primer at 50 °C for 45 min. This primer contains a 5p-phosphate group, a 6-randomized-nucleotide sequence, a 5p RNA adapter for Illumina sequencing, an 18-atom hexa-ethyleneglycol spacer (iSp18), and an R-RA3 region that is complementary to the 3p-end region of arti1 RNA. The reverse transcription mixtures were treated with 0.1 M NaOH for 15 min at 98 °C to degrade the RNAs. The retained cDNAs were gel-purified by 10% urea-PAGE and intramolecularily ligated using the CircLigase ssDNA ligase (Epicenter). The circularized cDNAs were separated from the linear cDNAs by 18% urea-PAGE and gel purified. The purified circularized cDNAs were amplified by PCR using RP1 and RPIx (Illumina RNA TruSeq preparation kit). Different indexed-RPIs were used for the control substrates, uncleaved substrates, and cleaved products to construct 3 different DNA libraries. This high-throughput processing assay and DNA library construction process was repeated 3 times. As a result, we obtained 9 DNA libraries that were subjected to the Illumina HiSeq X Ten sequencer using HiSeq X Ten Reagent Kit v2.5 (Illumina) in pair-end running mode.

### High-throughput human pri-miRNA processing assays

Two oligos, each containing a 3-nt randomized sequence, were annealed using the 20 bp complementary region and then annealed DNAs were extended using Klenow (Thermo Scientific) to synthesize each group of pri-mir-106a, pri-mir-342 or pri-mir-514a-1. The oligo information is presented in Supplementary Table 3. Subsequently, the Klenow-extended DNA duplexes were amplified by two PCR primers in which the forward primers contained the T7 promoter sequence (Supplementary Table 3). Finally, the RNAs were synthesized from the PCR-amplified DNA using IVT, as described for arti1.

In the high-throughput pri-mir-106a processing assays, 5 pmol of each pri-mir-106a group were incubated with 10 pmol of the D3-G2 complex in 10 µl standard reaction buffer and gel-purified similarly as described for arti1. The original substrates were reverse-transcribed using the R-RA3-6N-106a primer (Supplementary Table 3) and SuperScript™ IV Reverse Transcriptase. The synthesized cDNAs were then PCR-amplified using F-RA5-106a (Supplementary Table 3) and RPI4 (index: TGGTCA) primers. The PCR products obtained were further amplified using RP1 and RPI4 primers to get the final PCR products for NGS. The cleaved products (i.e., F2 fragment) of the processing assays were first ligated to the 3p-adapter (4N-RA3) using T4 RNA ligase 2, truncated KQ (NEB). The RA3-ligated cleaved RNAs were separated from the unligated RNAs and 4N-RA3 by 10% Urea-PAGE and then gel-purified. The purified RA3-ligated RNAs were further ligated to the 5p-adapter (RA5-4N) using T4 RNA ligase I (NEB). These two-adapter ligated RNAs were reverse-transcribed using the R-RA3 primer and SuperScript™ IV Reverse Transcriptase, and the synthesized cDNAs were amplified using RP1 and RPIx primers.

The high-throughput pri-miRNA processing assays were carried out similarly for pri-mir-514a-1 and pri-mir-342 variants using the D3TN1-G2 complex. The pooled original substrates or cleaved products (i.e., the F2-3 fragment) were first ligated to the 3p-adapter (4N-RA3) using T4 RNA ligase 2, truncated KQ. The 4N-RA3-ligated substrates were then processed similarly, as described for arti1. The 4N-RA3-ligated cleaved products were cloned similarly as described for the pri-mir-106a library.

The final PCR products were subjected to the Illumina Nextseq 500 sequencer using NSQ 500/550 High Output KT v2.5 (150 CYS) (Illumina) in single-end running mode.

### Analysis of high-throughput arti1 processing assays

The 3p-adapter and 5p-adapter in the raw reads were trimmed using cutadapt^41^ (cutadapt -a TGGAATTCTCGGGTGCCAAGG -A GATCGTCGGACTGTAGAACTCTGAAC). Paired-end reads were then joined together using fastq-join (default parameters)^42^, after which fastq_quality_filter (-q 20 -p 90) was used to filter the low-quality reads.

We then used fastx_collapser (http://hannonlab.cshl.edu/fastx_toolkit/index.html, version 0.0.13) to remove the duplicated reads containing the same 6-nt random barcode at the 5p-end. After discarding the barcode sequences, the processed reads were mapped to the reference sequences using BWA^43^. The reference sequence library contains 42,496 possible variants of the 11 groups. The perfectly mapped reads were selected for further analysis. Read cutoffs of 30 and 15 were used for the original substrate and cleaved product samples, respectively. In each sample, the raw read counts were normalized as the counts per million.

The cleavage score was calculated using the following formula: *P/S* = log_2_(*N*_*P*_ + 0.1)—log_2_(*N*_*S*_ + 0.1), where *N*_*S*_ is the normalized counts of the original substrate variant, and *N*_*P*_ is the normalized counts of the cleaved product derived from the same variant. The reciprocal cleavage score was estimated using *US/S* = log_2_(*N*_*US*_ + 0.1)—log_2_(*N*_*S*_ + 0.1), where *N*_*US*_ and *N*_*S*_, are the normalized counts of the uncleaved and original substrate, respectively, from the same variant. In addition, 0.1 was added as a pseudocount.

The positional cleavage scores were estimated for the cleaved products resulting from the different cleavage sites of DROSHA on the same variant, using the following formula: CLx cleavage score = log_2_(*N*_*X*_ + 0.1)—log_2_(*N*_*S*_ + 0.1). CLx is the cleavage site on the 5p-strand of arti1 RNA at x nucleotides from the basal junction, and *N*_*X*_ is the normalized counts of the cleaved products that have the first nucleotide mapped to position x + 1 of the corresponding substrate. Again, 0.1 was added as a pseudocount.

The relative positional cleavage frequency at different CLx for each variant was estimated by calculating: (The read counts of the cleaved products at CLx)/(the total read counts of the cleaved products at all the positions resulting from the same variant), such that x ranged from −6 to 1. The positional cleavage scores and the relative positional cleavage frequency of each variant were averaged using 3 scores from the 3 repeated experiments.

The secondary structure of each variant was predicted by RNAfold^44^ (ViennaRNA Package version 2.4.9) using the default settings. Since randomizing nucleotides can change the structure of other non-randomized regions, for further structural comparison, we selected the variants with a similar structure to the original backbone, but allowing for differences in the randomized regions with their 2 nucleotides upstream and downstream.

### Analysis of high-throughput human pri-miRNA processing assays

We analyzed the high-throughput pri-miRNA processing assays for pri-mir-342, pri-mir-514a-1, and pri-mir-106a similarly as described for arti1. After trimming the 3p-adapter and discarding the low-quality and duplicated reads, we cut out the random barcode sequences for the different samples as follows. We removed the 6-nt random barcode sequences at the 3p-end of the pri-mir-106a original substrates, the 6-nt random barcode sequences at the 5p-end and the 4-nt random barcode sequences at the 3p-end of the pri-mir-342 and pri-mir-514a-1 original substrates; and the 4-nt random barcode sequences at either end of the cleaved products for the three pri-miRNAs. The processed reads were then mapped to the reference sequences using BWA^43^. The reference sequences contained 53920, 39472, and 57856 variants generated from the pri-mir-342, pri-mir-514a-1, and pri-mir-106a backbones, respectively. The mapped reads were selected and analyzed as described for arti1.

### Preparation of the pri-miRNA substrates

Each dsDNA of an artificial pri-miRNA (arti_two junctions, arti2 or arti3) or pri-mir-16-1 with different stem lengths was made using 1-cycle PCR with a pair of synthetic ssDNA oligos as shown in Supplementary Tables 2 and 4. Each dsDNA of the human pri-miRNAs or the arti1_one junction WT was also synthesized by PCR using the specific primer pairs, and the DNA templates were either the genomic DNA from TUT4 KO Hela cells (a kind gift from Dr. Narry Kim) or the pri-miRNA-containing plasmids as shown in Supplementary Tables 2 and 5. The dsDNA sequences were confirmed by Sanger sequencing. 200 ng of each dsDNA was used in each 20 µl IVT reaction using the MEGAscript T7 transcription kit. The resulting RNAs were gel-purified and quantified.

### In vitro pri-miRNA processing assays

The pri-miRNA processing assay was carried out at 37 °C in 10 μl standard reaction buffer consisting of 50 mM Tris-HCl (pH 7.5), 150 mM NaCl, 10% glycerol, 0.2 μg/μl BSA, 1 mM DTT and 2 mM MgCl_2_. The amount of RNA substrate used, as well as the enzyme concentrations, and incubation time employed, are indicated in the various figures. The reaction was stopped by adding 10 μl 2× TBE-urea sample buffer and immediately chilling on ice, after which the stopped reaction mixture was treated with 20 μg proteinase K (Thermo Scientific) for 15 min at 37 °C followed by 15 min at 50 °C. Finally, the reaction mixture was heated at 95 °C for 5 min and then quickly chilled on ice before being loaded onto a pre-run 12% urea-PAGE gel in 1× TBE buffer. The gel was run at 300 V for 40 min or 75 min for further separation. After electrophoresis was completed, the gel was stained with SYBR^TM^ Green II RNA gel stain (Invitrogen) for 5 min, and images were captured with a Bio-Rad Gel Doc XR + system. RNA band intensities were estimated using Image Lab v6.0.1.

### Cloning of F2 fragments

To clone the F2 fragments resulting from the cleavage of pri-mir-342 and pri-mir-200b, and their variants, these fragments were cut from the gel and purified. The purified RNAs were ligated to the 5p-adapter using T4 RNA ligase 1. The RA5-ligated RNAs were reverse-transcribed using the R-RA3-342-F2 or R-RA3-200b-F2 primers (Supplementary Table 7) and SuperScript™ IV Reverse Transcriptase. Similarly, the gel-purified F2 fragments, which resulted from the cleavage of arti3 and its variants, were ligated to the 3p-adapter and 5p-adapter. The adapter-ligated F2 fragments were subsequently reverse-transcribed similarly as described for the cleaved product of pri-mir-106a. Finally, cDNAs were PCR-amplified using RP1 and RPIx primers, and the PCR products were subjected to NGS using the Illumina Nextseq 500 sequencer.

### Frequency of unmatched nucleotides in the upper stem of pri-miRNAs

The pri-miRNA sequences from different species were downloaded from MirGeneDB v2.0^30^. Each pri-miRNA sequence contains its pre-miRNA and a 30-nt extension at both pre-miRNA ends. We used RNAfold (the default parameter)^44^ to predict the secondary structure for each pri-miRNA. The position of unmatched nucleotides along the upper stem was determined for the 5p-strand only.

### Transfection and qPCR

The DNA regions encoding the pri-miRNA sequences were cloned into the pcDNA3 plasmid. The DNA cloning information is presented in Supplementary Table 6. The sequences of the cloned pcDNA3 plasmid were confirmed by Sanger sequencing. Four microgram wild-type (WT) or mutant pri-miRNA plasmid was co-transfected with 1 μg of pri-mir-16-1 pcDNA3 plasmid into HEK293T cells seeded in a 60-mm dish using 5 µg of L-PEI (Sigma) and 1% DMSO (Life Technologies). 5 μl P3000 and 7.5 μl Lipofectamine™ 3000 (Thermo scientific) were used instead of L-PEI when conducting the transfection with HCT116 cells. The total RNAs were extracted from the transfected cells two days after transfection using the miRNeasy mini kit (Qiagen). The HEK293T and HCT116 cells were obtained from Dr. Narry Kim.

For miRNA reverse transcription (RT), 50 ng total RNA, and stem-loop RT primers, which were designed for each miRNA, were used according to the reported method^45^. For pri-miRNA, 1 µg total RNA was added to the RT mixture containing 1 μM of each target-specific reverse primer. qPCR experiments of miRNAs and pri-miRNAs were performed using the iTaq Universal SYBR Green Supermix (Bio-Rad). The RT and qPCR primers are presented in Supplementary Table 8.

For small RNA sequencing, 9 µg of plasmid mixture was transfected into HCT116 cells seeded in a 100 mm-dish using Lipofectamine™ 3000. The components of the plasmid mixture are listed in Supplementary Table 9. Total RNAs were extracted using TRIzol reagent (Invitrogen). Small RNA sequencing libraries of the transfected HCT116 cells were generated using the TruSeq Small RNA Library Prep Kit.

### Analysis of small RNA sequencing

We trimmed the 3p-adapter using cutadapt (-a TGGAATTCTCGGGTGCCAAGG -m 18 -M 26) and removed low-quality reads using fastq_quality_filter (-q 20 -p 90). The surviving reads were then mapped to the human genome (hg38) using Bowtie2^46^. Unambiguously aligned reads with only mismatches at their 3p-end being allowed were collected. Next, we selected the unambiguous reads that mapped to the miRNA loci using the miRNA annotation obtained from miRBase v22 (miRbase.org). The unmapped reads from the previous step were aligned with the transfected mutant pri-miRNA sequences using Bowtie2^46^, and the resulting unambiguous reads as defined above from this alignment were collected. We calculated the relative abundance of miRNA using miR-16-5p as a normalization factor.

### Reporting summary

Further information on research design is available in the Nature Research Reporting Summary linked to this article.

## Supplementary information

Supplementary Information

Reporting Summary

## Data Availability

Sequencing data are available under accession GSE131636, GSE142140, and GSE142136 at Gene Expression Omnibus (GEO). The source data underlying Figs. 1d-e, 2d-i, 3d-i, 4b-c, f-i, k-m, 5d-e, h-o, 6e-f, i-p, 7a-l and Supplementary Figs. 1b-c, e, 2b, d-e, 3a-b, d-f, 4b-f, 5b-c, 6e-j, k, 7e-f, h-i are provided as a Source Data file. All data is available from the corresponding author upon reasonable request.

## Source data

Source Data
