# Author Correction: Mismatched and wobble base pairs govern primary microRNA processing by human Microprocessor

**DOI:** 10.1038/s41467-021-26533-z

**Published:** 2021-10-26

**Authors:** Shaohua Li, Trung Duc Nguyen, Thuy Linh Nguyen, Tuan Anh Nguyen

**Affiliations:** Division of Life Science, The Hong Kong University of Science and Technology, Hong Kong, China

**Keywords:** Biochemistry, Molecular biology

Correction to: *Nature Communications* 10.1038/s41467-020-15674-2, published online 21 April 2020.

The original version of this Article contained an error in Fig. 7, in which the wrong data were used for panel f. The correct version of Fig. 7f is:
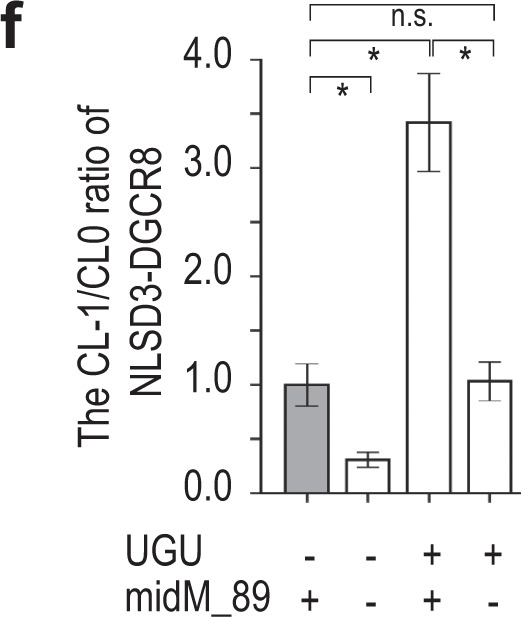


which replaces the previous incorrect version:
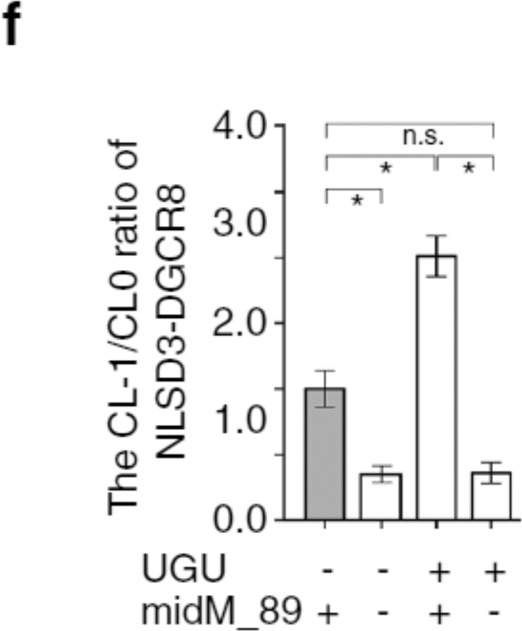


This has been corrected in both the PDF and HTML versions of the Article.

Additionally, the original version of the Source Data File associated with this Article contained errors for data underlying Fig. 7e and f. The HTML has been updated to include a corrected version of the Source Data File.

