# Supplementary material for: Mismatched and wobble base pairs govern primary microRNA processing by human Microprocessor
Source: Nat Commun. 2020 Apr 21;11:1926. doi: 10.1038/s41467-020-15674-2 (PMC7174388; doi:10.1038/s41467-020-15674-2)
Supplement: Supplementary file 1 — Supplementary Information [file 41467_2020_15674_MOESM1_ESM.pdf]

Mismatched and wobble base pairs govern primary microRNA processing by human Microprocessor

Li *et al.*

Supplementary Figure 1

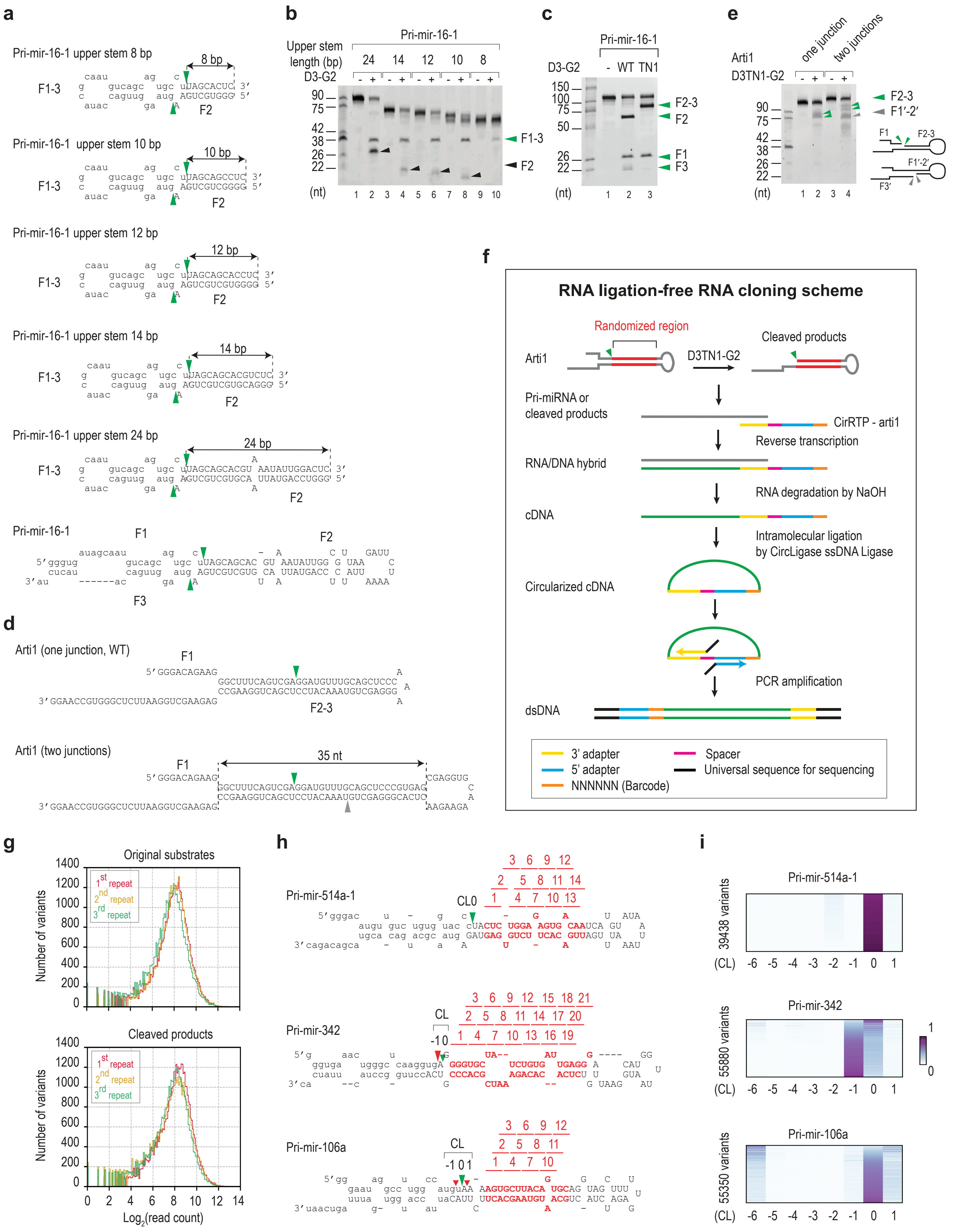

Supplementary Fig. 1 High-throughput pri-miRNA processing assays. **a** Diagram of pri-mir-16-1 and one-junction pri-miRNA variants, which were based on pri-mir-16-1. The green arrowheads show the canonical cleavage sites of DROSHA, and the capital letters indicate pre-mir-16-1. F1-3 indicates the fragments resulting from the connection between the F1 and F3 fragments. **b** Processing of pri-mir-16-1 variants by D3-G2. Each RNA (6 pmol) was incubated with D3-G2 (5 pmol) for 60 min at 37°C in 10 μl standard reaction buffer. **c** Processing of pri-mir-16-1 by D3-G2 or D3TN1-G2. Each RNA (6 pmol) was incubated with D3-G2 or D3TN1-G2 (5 pmol) for 60 min at 37°C in 10 μl standard reaction buffer. **d** Diagram of arti1 with one junction and elongated arti1 with two junctions. **e** Processing of one-junction and two-junction arti1 substrates by D3TN1-G2. Each RNA (6 pmol) was incubated with D3TN1-G2 (10 pmol) for 120 min at 37°C in 10 μl standard reaction buffer. The green and grey arrowheads indicate the productive and unproductive cleavages of DROSHA, respectively. **f** RNA cloning scheme of the high-throughput arti1 processing assays. The substrates (arti1), uncleaved substrates, and cleaved products were reverse transcribed using the same primer, cirRTP-arti1. The sequence information of the primer is presented in Supplementary Table 2. A detailed description of the process is described in the Methods section. **g** Plots showing the distribution of read counts for the arti1 variants from the arti1 high-throughput pri-miRNA processing. **h** Diagram of the human pri-miRNAs, pri-mir-514a-1, pri-mir-342, and pri-mir-106a. The arrowheads indicate DROSHA cleavage site, and the red letters are in the randomized region, which is divided into the different groups, as shown in the figure. Each group contains 3 pairs of randomized nucleotides. **i** The relative positional cleavage frequency of DROSHA at different positions ranging from -6 to 1 from the canonical cleavage site of each pri-miRNA. The cleaved products that resulted from each variant substrate were collected, and their relative percentage was estimated between 0 and 1, colored according to the color bar (right). The source data are provided in the Source Data file.

Supplementary Figure 2

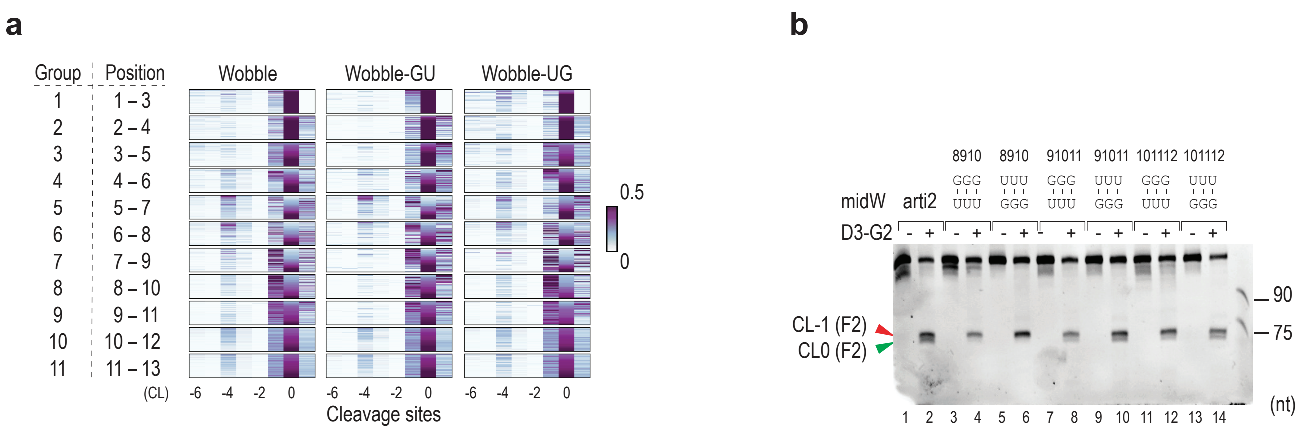

c

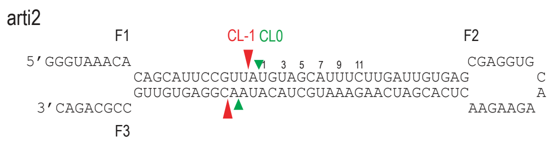

midM	nt	midM	nt	midM	nt	midM	nt
7	A<br>A	8	U<br>C	78	AU<br>AC	789	AUU<br>ACC
		9	U<br>C	89	UU<br>CC	8910	UUU<br>CCC

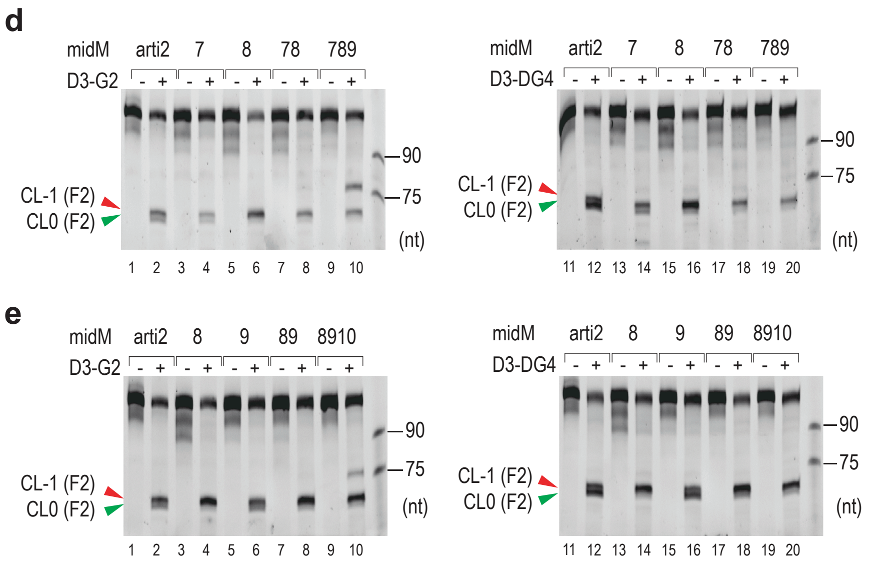

Supplementary Fig. 2 MidMW induces the alternative cleavages of DROSHA. **a** The relative positional cleavage frequency of DROSHA at different positions ranging from 7 to 14 nt from the basal junction of each variant from an arti1 individual group. The positional cleavage frequency was estimated for wobble, G-U, and U-G variants, and is colored according to the color bar (right). **b** Processing of arti2 pri-miRNA by D3-G2. The arti2 variants contain G-U or U-G at different positions. Each RNA (6 pmol) was incubated with D3-G2 (10 pmol) for 120 min at 37°C in 10 µl standard reaction buffer. **c** Diagram of arti2. The red and green arrowheads indicate the CL-1 and CL0 cleavage sites of DROSHA, respectively. The table shows the various arti2 variants with mismatches at different positions. **d**, **e** Processing of arti2 pri-miRNA by D3-G2 or D3-DG4. The arti2 variants contain mismatches at different positions. Each RNA (6 pmol) was incubated with D3-G2 (10 pmol) or D3-DG4 (6 pmol) for 120 min at 37°C in 10 µl standard reaction buffer. The source data are provided in the Source Data file.

Supplementary Figure 3

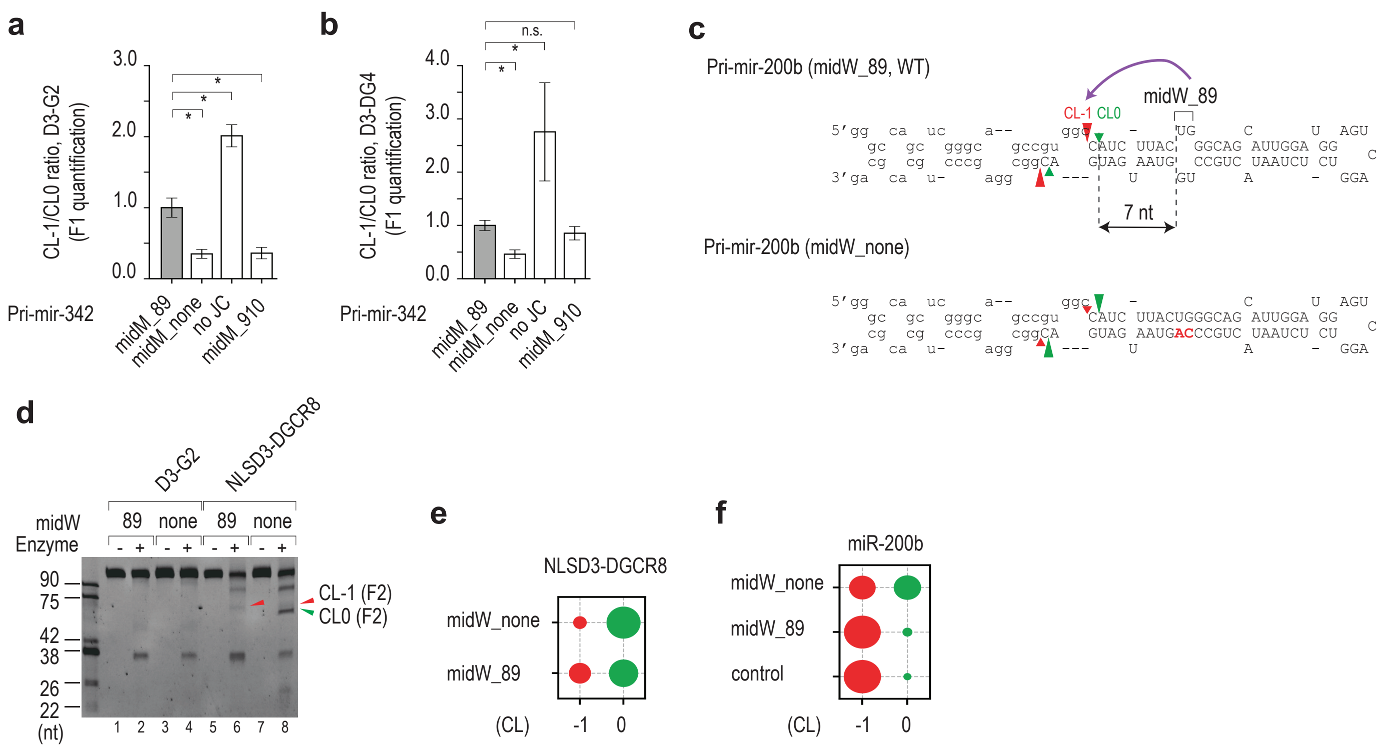

Supplementary Fig. 3 MidMW induces the alternative cleavage of DROSHA on human pri-miRNAs. **a**, **b** Bar graphs to show the relative alternative cleavage activity of D3-G2 (Fig. 3b) or D3-DG4 (Fig. 3e), which was estimated from experiments conducted in triplicate as a ratio of CL-1 to CL0 cleavage. The band densities of the F1 fragments resulting from the CL-1, and CL0 cleavages were measured using Image Lab v.6.0.1. Data are presented as mean values +/- SEM. The asterisks (*) and n.s. indicate statistical significant differences and no statistical significant differences, respectively, from the two-sided t-test (**a** midM_none vs. midM_89: $p = 0.012$, no_JC vs. midM_89: $p = 0.008$, midM_910 vs. midM_89: $p = 0.015$; **b** midM_none vs. midM_89: $p = 0.013$, no_JC vs. midM_89: $p = 0.003$, midM_910 vs. midM_89: $p = 0.408$). **c** Diagrams of pri-mir-200b and its variant. The red and green arrowheads indicate the CL-1 and CL0 cleavages of DROSHA, respectively. The letters in red represent mutated nucleotides. The capital letters are pre-mir-200b. **d** Processing of pri-mir-200b by D3-G2 or NLSD3-DGCR8. Each RNA (6 pmol) was incubated with D3-G2 (10 pmol) or NLSD3-DGCR8 (4 pmol) for 120 min at 37°C in 10 µl standard reaction buffer. **e** The F2 fragments resulting from the CL-1 and CL0 cleavages were cloned and sequenced by NGS. The size of the circle indicates the relative amount of each F2 fragment from the NGS data. **f** The expression of miR-200b in human cells, which were transfected with plasmids expressing pri-mir-200b (midW_89, WT) or pri-mir-200b (midW_none), was estimated by miRNA sequencing. The size of each circle indicates the relative amount of each miRNA isomer from the miRNA sequencing data. The source data are provided in the Source Data file.

Supplementary Figure 4

a

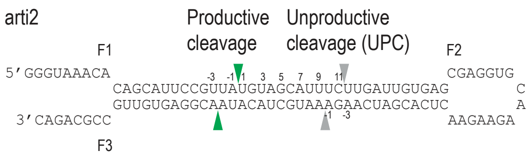

Variants	nt	Variants	nt	Variants	nt
-3-2-1	UUA<br>CCC	123	UGU<br>UAU	-1	A<br>C
-2-1 1	UAU<br>CCC	-3	U<br>C	-3-2	UU<br>CC
-1 1 2	AUG<br>CCA	-2	U<br>C	-2-1	UA<br>CC

midM	nt	midM	nt
10	U<br>C	1011	UC<br>CC
11	C<br>C	101112	UCU<br>CCC

midW	nt	midW	nt
10	G<br>U	1011	GG<br>UU
11	G<br>U	101112	GGG<br>UUU

b

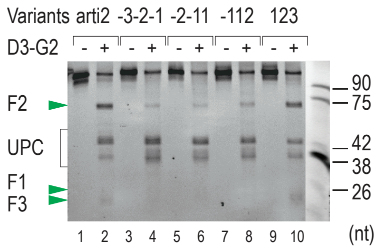

c

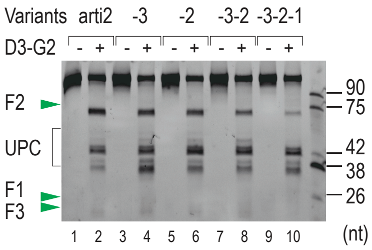

d

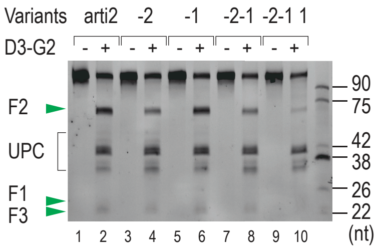

e

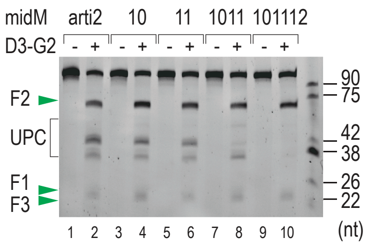

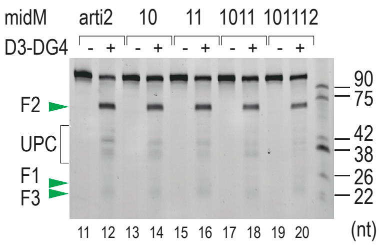

f

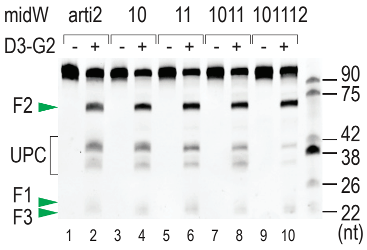

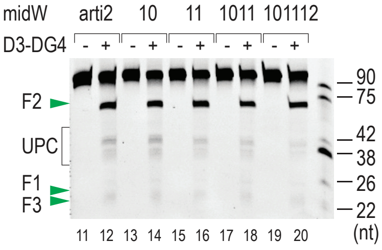

Supplementary Fig. 4 MidMW reduces the unproductive cleavage of DROSHA. **a** Diagram of arti2. The green and grey arrowheads indicate the productive and unproductive cleavage sites of DROSHA, respectively. The table shows the various arti2 variants with mismatches and wobble base pairs at different positions. **b**–**f** Processing of arti2 variants by D3-G2 or D3-DG4. The positions of mismatches or wobble base pairs are shown. Each RNA (6 pmol) was incubated with D3-G2 (10 pmol) or D3-DG4 (6 pmol) for 120 min at 37°C in 10 µl standard reaction buffer. UPC: unproductive cleavage. The source data are provided in the Source Data file.

Supplementary Figure 5

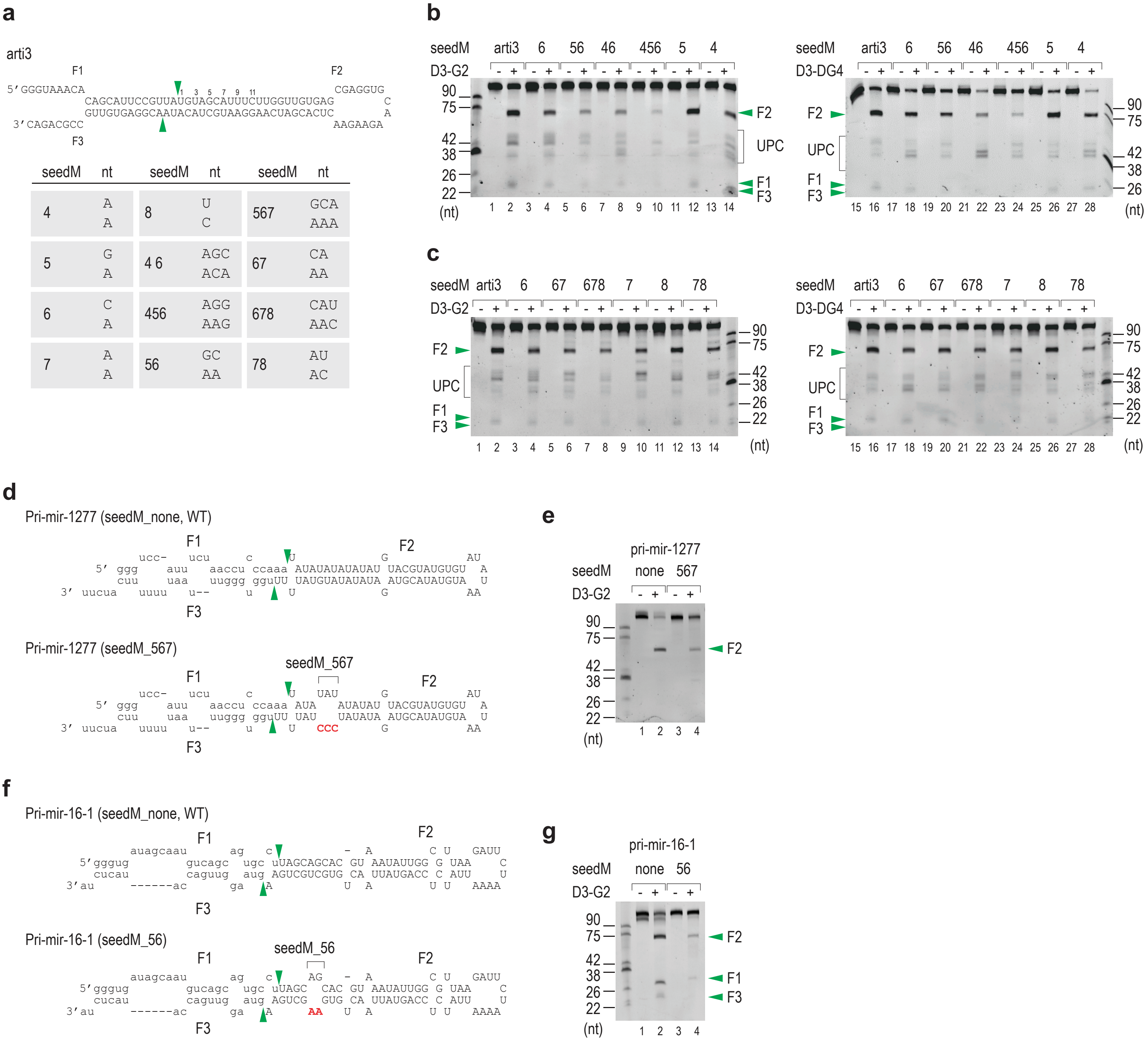

Supplementary Fig. 5 SeedM inhibits the productive cleavage of DROSHA. **a** Diagram of arti3. The green arrowheads indicate the productive cleavage sites of DROSHA. The table shows the various arti3 variants with mismatches at different positions. **b**, **c** Processing of arti3 pri-miRNA by D3-G2 and D3-DG4. The positions of mismatches are shown. Each RNA (6 pmol) was incubated with D3-G2 (10 pmol) or D3-DG4 (6 pmol) for 120 min at 37°C in 10 µl standard reaction buffer. **d**, **f** Diagram of pri-mir-1277 (d) and pri-mir-16-1 (f) and their variants. The green arrowheads indicate the canonical cleavage sites of DROSHA. **e**, **g** Processing of pri-mir-1277 (e) or pri-mir-16-1 (g) by D3-G2. Each RNA (6 pmol) was incubated with D3-G2 (5 pmol) for 60 min at 37°C in 10 µl standard reaction buffer. UPC: unproductive cleavage. The source data are provided in the Source Data file.

Supplementary Figure 6

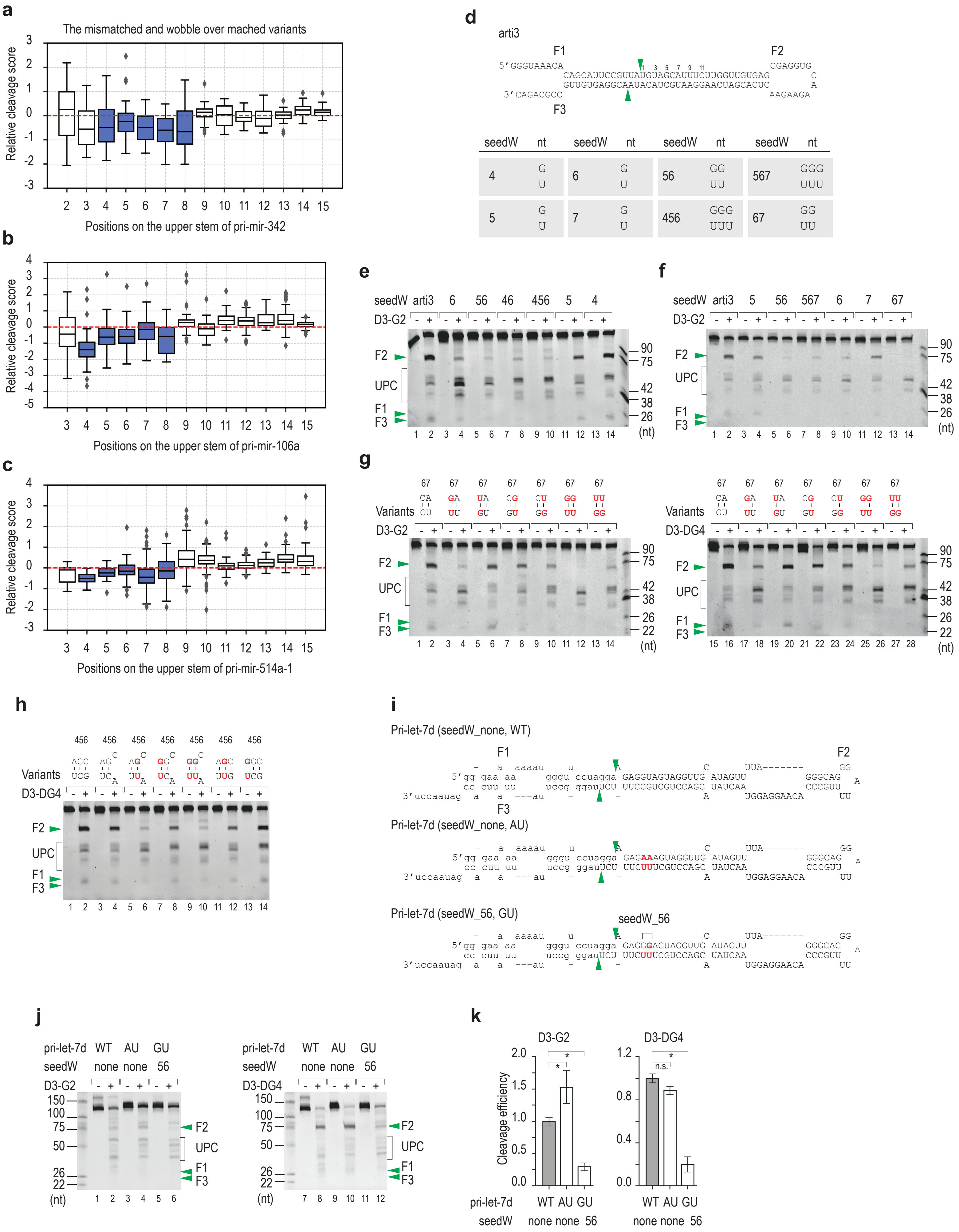

Supplementary Fig. 6 SeedW inhibits the productive cleavage of DROSHA. **a**–**c** The efficiency of the productive cleavage of DROSHA estimated from the pri-mir-342 (a), pri-mir-106a (b), and pri-mir-514a-1 (c) high-throughput processing assays. The cleavage score of canonical cleavage was estimated for each variant. The relative cleavage score of variants containing a mismatch or wobble base pair at a position was normalized against that of the variants containing a Watson-Crick base pair at the same position. **d** Diagram of arti3. The green arrowheads indicate the productive cleavage sites of DROSHA. The table shows the various arti3 variants with wobble base pairs at different positions. **e**–**h** Processing of arti3 pri-miRNA by D3-G2 or D3-DG4. The positions of wobble base pairs are shown. Each RNA (6 pmol) was incubated with D3-G2 (10 pmol) or D3-DG4 (6 pmol) for 120 min at 37°C in 10 µl standard reaction buffer. **i** Diagram of pri-let-7d and its variants. The green arrowheads indicate the canonical cleavage sites of DROSHA. The red letters are mutated nucleotides. The capital letters represent pre-let-7d. **j** Processing of pri-let-7d and its variants by D3-G2 or D3-DG4. Each RNA (6 pmol) was incubated with D3-G2 (5 pmol) or D3-DG4 (3 pmol) for 120 min at 37°C in 10 µl standard reaction buffer. **k** Bar graphs show the cleavage efficiency of D3-G2 or D3-DG4 on pri-let-7d and its variants. The cleavage efficiency was estimated from the repeated experiments as the ratio of the F2 band density to that of pri-miRNA. The band densities were measured using Image Lab v.6.0.1. Data are presented as mean values +/- SEM, n = 3. The asterisks (*) and n.s. indicate statistical significant differences and no statistical significant differences, respectively, from the two-sided t-test (left graph seedW_none_WT vs seedW_none_AU: p = 0.001, seedW_none_WT vs. seedW_56: p = 3.7e-5; right graph seedW_none_WT vs. seedW_none_AU: p = 0.400, seedW_none_WT vs. seedW_56: p = 0.032). UPC: unproductive cleavage. The source data are provided in the Source Data file.

Supplimentary figure 7

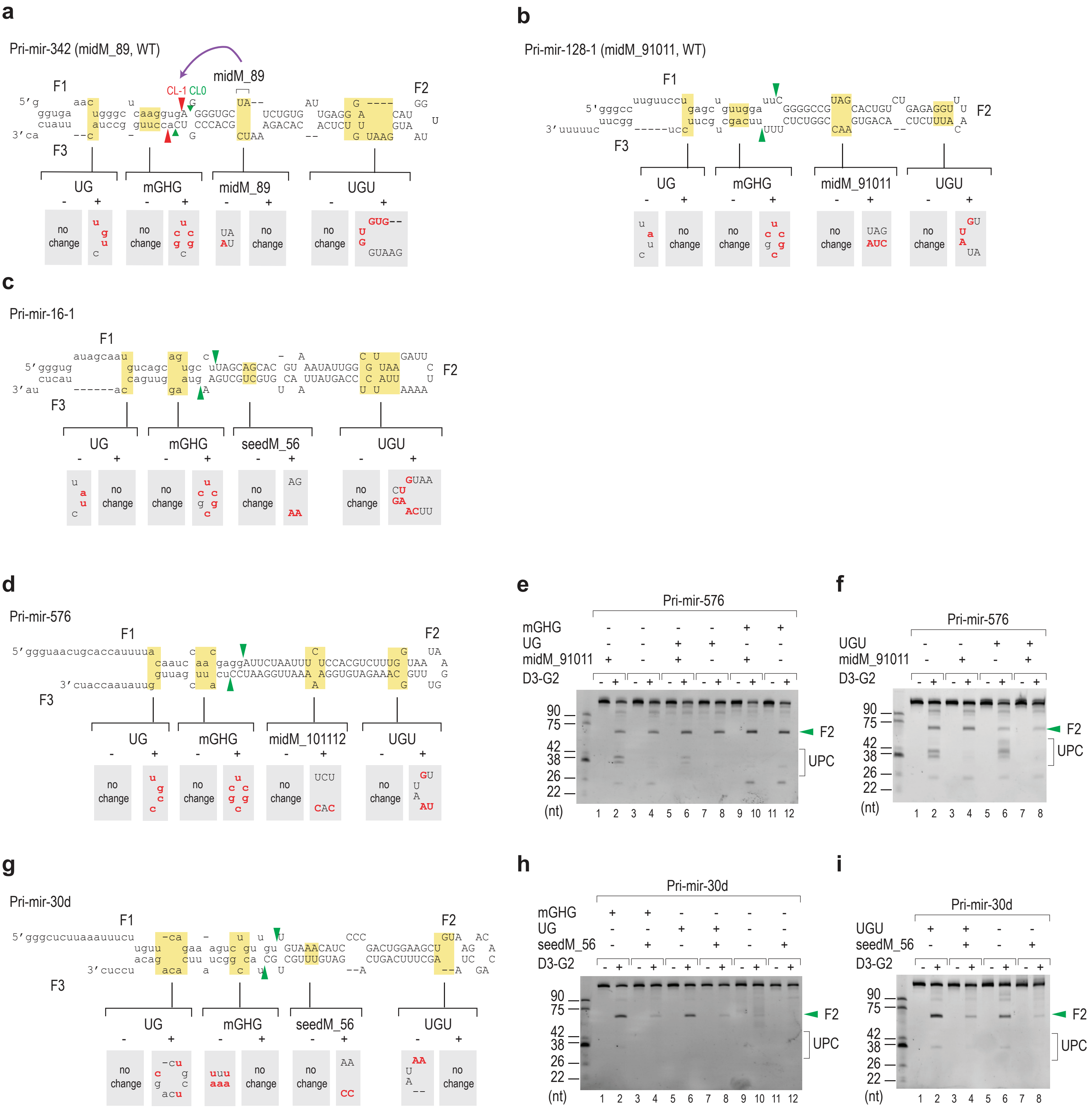

Supplementary Fig. 7 Coordination of midMW and seedMW with UG, UGU, and mGHG in determining the accuracy and efficiency of Microprocessor cleavages. **a**–**d**, **g** Diagram of the various pri-miRNAs and their variants. The green and red arrowheads indicate the productive and alternative cleavage sites of DROSHA, respectively. The mutated nucleotides are in red. **e**, **f**, **h**, **i** Processing of pri-miRNAs and their variants by D3-G2. Each RNA (6 pmol) was incubated with D3-G2 (5 pmol) for 60 min at 37°C in 10 μl standard reaction buffer. UPC: unproductive cleavage. The source data are provided in the Source Data file.

Supplementary Table 1. The protein expression plasmids

Plasmid name	Plasmid backbone, fusion tags	Protein name, cloned region, mutation site
pXab-D3	pXab, C-terminal protein G-10×His	DROSHA, amino acids 390–1365
pXab-D3TN1	pXab, C-terminal protein G-10×His	DROSHA, amino acids 390–1365, E1045Q
pXab-NLSD3	pXab, C-terminal protein G-10×His	NLS (PKKKRKV); DROSHA, amino acids 390–1365
pXG-G2	pXG, C-terminal GFP-10×His	DGCR8, amino acids 701–773
pXG-DG4	pXG, C-terminal GFP-10×His	DGCR8, amino acids 285–773
pXG-DGCR8	pXG, C-terminal GFP-10×His	DGCR8, full length

Supplementary Table 2. The PCR primers and templates for arti1 RNA preparation

The underlined sequence is T7 promoter.

Primer name	Primer sequence (5'-3')
F-T7-arti1	TAATACGACTCACTATAGGGACAGAAGGGCTTTCAGTC
RTP-RA3-arti1	CCTTGGCACCCGAGAATTCCAGCTTCTCGGCTTCCAG
cirRTP	/5Phos/NNNNNNGATCGTCGGACTGTAGAACTCTGAAC/iSp18/CCTTGGCACCCGAGAATTCCA
F-arti1_two junctions	GGGACAGAAGGGCTTTCAGTCGAGGATGTTTGCAGCTCCCGTGAGCGAGGTGCAAGAAGAACTC
R-arti1_two junctions	GCTTCTCGGCTTCCAGTCGAGGATGTTTACAGCTCCCGTGAGTTCTTCTTGCACCTCGCTC
Group number	ssDNA PCR template sequences (5'-3')
arti1(one junction, WT)	GGGACAGAAGGGCTTTCAGTCGAGGATGTTTGCAGCTCCCAAAGGGAGCTGTAAACATCCTCGACTGGAAGCCGAGAAGC
1	GGGACAGAAGGGCTTTCAGTCGANNNTGTTTGCAGCTCCCAAAGGGAGCTGTAAACANNNTCGACTGGAAGCCGAGAAGC
2	GGGACAGAAGGGCTTTCAGTCGAGNNNGTTTGCAGCTCCCAAAGGGAGCTGTAAACNNNCTCGACTGGAAGCCGAGAAGC
3	GGGACAGAAGGGCTTTCAGTCGAGGNNNTTTGCAGCTCCCAAAGGGAGCTGTAAANNNCCTCGACTGGAAGCCGAGAAGC
4	GGGACAGAAGGGCTTTCAGTCGAGGANNNTTGCAGCTCCCAAAGGGAGCTGTAANNNTCCTCGACTGGAAGCCGAGAAGC
5	GGGACAGAAGGGCTTTCAGTCGAGGATNNNTGCAGCTCCCAAAGGGAGCTGTANNNATCCTCGACTGGAAGCCGAGAAGC
6	GGGACAGAAGGGCTTTCAGTCGAGGATGNNNGCAGCTCCCAAAGGGAGCTGTNNNCATCCTCGACTGGAAGCCGAGAAGC
7	GGGACAGAAGGGCTTTCAGTCGAGGATGTNNNCAGCTCCCAAAGGGAGCTGNNNACATCCTCGACTGGAAGCCGAGAAGC
8	GGGACAGAAGGGCTTTCAGTCGAGGATGTTNNNAGCTCCCAAAGGGAGCTNNNAACATCCTCGACTGGAAGCCGAGAAGC
9	GGGACAGAAGGGCTTTCAGTCGAGGATGTTTNNNGCTCCCAAAGGGAGCNNNAAACATCCTCGACTGGAAGCCGAGAAGC
10	GGGACAGAAGGGCTTTCAGTCGAGGATGTTTGNNNCTCCCAAAGGGAGNNNTAAACATCCTCGACTGGAAGCCGAGAAGC
11	GGGACAGAAGGGCTTTCAGTCGAGGATGTTTGCNNNTCCCAAAGGGANNNGTAAACATCCTCGACTGGAAGCCGAGAAGC

Supplementary Table 3. The PCR primers and templates for pri-mir-106a, pri-mir-514a-1 and pri-mir-342 RNA preparation and library cloning

The underlined sequence is T7 promoter.

Primer name	Primer sequence (5'-3')
F-106a-G1	GGAATAGGCCTTGGCCATGTAAANNNGCTTACAGTGCAGGTAGCTTTTTGAGATCTACTG
F-106a-G2	GGAATAGGCCTTGGCCATGTAAAANNNCTTACAGTGCAGGTAGCTTTTTGAGATCTACT
F-106a-G3	GGAATAGGCCTTGGCCATGTAAAAGNNNTTACAGTGCAGGTAGCTTTTTGAGATCTACT
F-106a-G4	GGAATAGGCCTTGGCCATGTAAAAGTNNNTACAGTGCAGGTAGCTTTTTGAGATCTACT
F-106a-G5	GGAATAGGCCTTGGCCATGTAAAAGTGNNNACAGTGCAGGTAGCTTTTTGAGATCTACTG
F-106a-G6	GGAATAGGCCTTGGCCATGTAAAAGTGCNNNCAGTGCAGGTAGCTTTTTGAGATCTACT
F-106a-G7	GGAATAGGCCTTGGCCATGTAAAAGTGCTNNNAGTGCAGGTAGCTTTTTGAGATCTACTG
F-106a-G8	GGAATAGGCCTTGGCCATGTAAAAGTGCTTNNNGTGCAGGTAGCTTTTTGAGATCTACT
F-106a-G9	GGAATAGGCCTTGGCCATGTAAAAGTGCTTANNNTGCAGGTAGCTTTTTGAGATCTACT
F-106a-G10	GGAATAGGCCTTGGCCATGTAAAAGTGCTTACNNNGCAGGTAGCTTTTTGAGATCTACT
F-106a-G11	GGAATAGGCCTTGGCCATGTAAAAGTGCTTACANNNCAGGTAGCTTTTTGAGATCTACT

F-106a-G12	GGAATAGGCCTTGGCCATGTAAAAGTGCTTACAGNNNAGGTAGCTTTTTGAGATCTACT
R-106a-G1	CTAAATCACCATGGTAATGTAAGANNNGCTTACATTGCAGTAGATCTCAAAAAGCTA
R-106a-G2	CTAAATCACCATGGTAATGTAAGAANNNCTTACATTGCAGTAGATCTCAAAAAGCTAC
R-106a-G3	CTAAATCACCATGGTAATGTAAGAAGNNNTTACATTGCAGTAGATCTCAAAAAGCTAC
R-106a-G4	CTAAATCACCATGGTAATGTAAGAAGTNNNTACATTGCAGTAGATCTCAAAAAGCTAC
R-106a-G5	CTAAATCACCATGGTAATGTAAGAAGTGNNNACATTGCAGTAGATCTCAAAAAGCTA
R-106a-G6	CTAAATCACCATGGTAATGTAAGAAGTGCNNNCATTGCAGTAGATCTCAAAAAGCTAC
R-106a-G7	CTAAATCACCATGGTAATGTAAGAAGTGCTNNNATTGCAGTAGATCTCAAAAAGCTA
R-106a-G8	CTAAATCACCATGGTAATGTAAGAAGTGCTTNNNTTGCAGTAGATCTCAAAAAGCTAC
R-106a-G9	CTAAATCACCATGGTAATGTAAGAAGTGCTTANNNTGCAGTAGATCTCAAAAAGCTAC
R-106a-G10	CTAAATCACCATGGTAATGTAAGAAGTGCTTACNNNGCAGTAGATCTCAAAAAGCTAC
R-106a-G11	CTAAATCACCATGGTAATGTAAGAAGTGCTTACANNNCAGTAGATCTCAAAAAGCTAC
R-106a-G12	CTAAATCACCATGGTAATGTAAGAAGTGCTTACATNNNAGTAGATCTCAAAAAGCTAC
R-106aMut-G7	CTAAATCACCATGGTAATGTAAGAAGTGCTNNNAGTGCAGTAGATCTCAAAAAGCTAC
R-106aMut-G8	CTAAATCACCATGGTAATGTAAGAAGTGCTTNNNGTGCAGTAGATCTCAAAAAGCTAC
R-106aMut-G12	CTAAATCACCATGGTAATGTAAGAAGTGCTTACAGNNNAGTAGATCTCAAAAAGCTAC
F-T7-106a	TAATACGACTCACTATAGGGAATAGGCCTTGGCCATGT
R-106a	ATTGACTAAATCACCATGGTAATGT
R-RA3-6N-106a	TTGGCACCCGAGAATTCCANNNNNNATTGACTAAATCACCATGGTAATGT
F-RA5-106a	GTTCAGAGTTCTACAGTCCGACGATCGGAATAGGCCTTGGCCATGT
R-RA3-6N-106a	TTGGCACCCGAGAATTCCANNNNNNATTGACTAAATCACCATGGTAATGT
F-514a-1_G01	ACATGTTGTCTGTGGTACCCTANNNTGGAGAGTGACAATCATGTATAATTAAATTTGA
R-514a-1_G01	TCGTACGTGTCATGCGTTACTCTANNNACAGAAGTGTCAATCAAATTTAATTATACATGA
F-514a-1_G02	ACATGTTGTCTGTGGTACCCTACNNTGGAGAGTGACAATCATGTATAATTAAATTTGA
R-514a-1_G02	TCGTACGTGTCATGCGTTACTCTACNNNCAGAAGTGTCAATCAAATTTAATTATACATGA
F-514a-1_G03	ACATGTTGTCTGTGGTACCCTACTNNGGAGAGTGACAATCATGTATAATTAAATTTGA
R-514a-1_G03	TCGTACGTGTCATGCGTTACTCTACTNNNAGAAGTGTCAATCAAATTTAATTATACATGA
F-514a-1_G04	ACATGTTGTCTGTGGTACCCTACTCNNNAGAGTGACAATCATGTATAATTAAATTTGA
R-514a-1_G04	TCGTACGTGTCATGCGTTACTCTACTCANNNAAGTGTCAATCAAATTTAATTATACATGA
F-514a-1_G05	ACATGTTGTCTGTGGTACCCTACTCTNNNGAGTGACAATCATGTATAATTAAATTTGA
R-514a-1_G05	TCGTACGTGTCATGCGTTACTCTACTCACNNNAGTGTCAATCAAATTTAATTATACATGA
F-514a-1_G06	ACATGTTGTCTGTGGTACCCTACTCTGNNNAGTGACAATCATGTATAATTAAATTTGA
R-514a-1_G06	TCGTACGTGTCATGCGTTACTCTACTCACANNAGTGTCAATCAAATTTAATTATACATGA
F-514a-1_G07	ACATGTTGTCTGTGGTACCCTACTCTGGNNNGTGACAATCATGTATAATTAAATTTGA
R-514a-1_G07	TCGTACGTGTCATGCGTTACTCTACTCACAGNNGTGTCAATCAAATTTAATTATACATGA
F-514a-1_G08	ACATGTTGTCTGTGGTACCCTACTCTGGANNNTGACAATCATGTATAATTAAATTTGA
R-514a-1_G08	TCGTACGTGTCATGCGTTACTCTACTCACAGANNTGTCAATCAAATTTAATTATACATGA
F-514a-1_G09	ACATGTTGTCTGTGGTACCCTACTCTGGAGNNNGACAATCATGTATAATTAAATTTGA
R-514a-1_G09	TCGTACGTGTCATGCGTTACTCTACTCACAGANNNGTCAATCAAATTTAATTATACATGA
F-514a-1_G10	ACATGTTGTCTGTGGTACCCTACTCTGGAGANNNACAATCATGTATAATTAAATTTGA
R-514a-1_G10	TCGTACGTGTCATGCGTTACTCTACTCACAGAANNNTCAATCAAATTTAATTATACATGA
F-514a-1_G11	ACATGTTGTCTGTGGTACCCTACTCTGGAGAGNNNCAATCATGTATAATTAAATTTGA
R-514a-1_G11	TCGTACGTGTCATGCGTTACTCTACTCACAGAAGNNNCAATCAAATTTAATTATACATGA
F-514a-1_G12	ACATGTTGTCTGTGGTACCCTACTCTGGAGAGTNNNAATCATGTATAATTAAATTTGATTNNNACTTCTGTGAGTAGAGTAACGCA
F-514a-1_G13	ACATGTTGTCTGTGGTACCCTACTCTGGAGAGTGNNNATCATGTATAATTAAATTTGATNNNCACTTCTGTGAGTAGAGTAACGCA
F-514a-1_G14	ACATGTTGTCTGTGGTACCCTACTCTGGAGAGTGANNNTCATGTATAATTAAATTTGANNNACACTTCTGTGAGTAGAGTAACGCA
R-514a-1_G12-14	TCGTACGTGTCATGCGTTACTCTACTCACAGA
F-T7-514a-1	TAATACGACTCACTATAGGGACATGTTGTCTGTGGTACCC
R-514a-1	GTCTGTCGTACGTGTCATGCG
F-342_G01	GTGAAACTGGGCTCAAGGTGAGNNNTGCTATCTGTGATTGAGGGACATGGTTAATGGAATTGT
R-342_G01	TGGTGATAAGTAGGCCAAGGTGACNNNTGCGATTTCTGTGTGAGACAATTCCATTAACCATGTCCC
F-342_G02	GTGAAACTGGGCTCAAGGTGAGGNNNGCTATCTGTGATTGAGGGACATGGTTAATGGAATTGT
R-342_G02	TGGTGATAAGTAGGCCAAGGTGACGNNNGCGATTTCTGTGTGAGACAATTCCATTAACCATGTCCC
F-342_G03	GTGAAACTGGGCTCAAGGTGAGGGNNNCTATCTGTGATTGAGGGACATGGTTAATGGAATTGT
R-342_G03	TGGTGATAAGTAGGCCAAGGTGACGGNNNCGATTTCTGTGTGAGACAATTCCATTAACCATGTCCC
F-342_G04	GTGAAACTGGGCTCAAGGTGAGGGGNNNTATCTGTGATTGAGGGACATGGTTAATGGAATTGT
R-342_G04	TGGTGATAAGTAGGCCAAGGTGACGGGNNNGATTTCTGTGTGAGACAATTCCATTAACCATGTCCC

F-342_G05	GTGAAACTGGGCTCAAGGTGAGGGGTNNNATCTGTGATTGAGGGACATGGTTAATGGAATTGT
R-342_G05	TGGTGATAAGTAGGCCAAGGTGACGGGTNNNATTTCTGTGTGAGACAATTCCATTAACCATGTCCC
F-342_G06	GTGAAACTGGGCTCAAGGTGAGGGGTGNNNTCTGTGATTGAGGGACATGGTTAATGGAATTGT
R-342_G06	TGGTGATAAGTAGGCCAAGGTGACGGGTGNNNTTTCTGTGTGAGACAATTCCATTAACCATGTCCC
F-342_G07	GTGAAACTGGGCTCAAGGTGAGGGGTGCNNTCTGTGATTGAGGGACATGGTTAATGGAATTGT
R-342_G07	TGGTGATAAGTAGGCCAAGGTGACGGGTGCNNNTTCTGTGTGAGACAATTCCATTAACCATGTCCC
F-342_G08	GTGAAACTGGGCTCAAGGTGAGGGGTGCNNTCTGTGATTGAGGGACATGGTTAATGGAATTGT
R-342_G08	TGGTGATAAGTAGGCCAAGGTGACGGGTGCGNNNTCTGTGTGAGACAATTCCATTAACCATGTCCC
F-342_G09	GTGAAACTGGGCTCAAGGTGAGGGGTGCTNNNTGTGATTGAGGGACATGGTTAATGGAATTGT
R-342_G09	TGGTGATAAGTAGGCCAAGGTGACGGGTGCGATNNNTGTGTGAGACAATTCCATTAACCATGTCCC
F-342_G10	GTGAAACTGGGCTCAAGGTGAGGGGTGCTANNNGTGATTGAGGGACATGGTTAATGGAATTGT
R-342_G10	TGGTGATAAGTAGGCCAAGGTGACGGGTGCGATTNNNGTGTGAGACAATTCCATTAACCATGTCCC
F-342_G11	GTGAAACTGGGCTCAAGGTGAGGGGTGCTATNNNTGATTGAGGGACATGGTTAATGGAATTGT
R-342_G11	TGGTGATAAGTAGGCCAAGGTGACGGGTGCGATTTNNNTGTGAGACAATTCCATTAACCATGTCCC
F-342_G12	GTGAAACTGGGCTCAAGGTGAGGGGTGCTATCNNNGATTGAGGGACATGGTTAATGGAATTGT
R-342_G12	TGGTGATAAGTAGGCCAAGGTGACGGGTGCGATTTCNNNGTGAGACAATTCCATTAACCATGTCCC
F-342_G13	GTGAAACTGGGCTCAAGGTGAGGGGTGCTATCTNNNATTGAGGGACATGGTTAATGGAATTGT
R-342_G13	TGGTGATAAGTAGGCCAAGGTGACGGGTGCGATTTCTNNNTGAGACAATTCCATTAACCATGTCCC
F-342_G14	GTGAAACTGGGCTCAAGGTGAGGGGTGCTATCTGNNNTTGAGGGACATGGTTAATGGAATTGT
R-342_G14	TGGTGATAAGTAGGCCAAGGTGACGGGTGCGATTTCTGNNNGAGACAATTCCATTAACCATGTCCC
F-342_G15	GTGAAACTGGGCTCAAGGTGAGGGGTGCTATCTGTNNNTGAGGGACATGGTTAATGGAATTGT
R-342_G15	TGGTGATAAGTAGGCCAAGGTGACGGGTGCGATTTCTGTNNGAGACAATTCCATTAACCATGTCCC
F-342_G16	GTGAAACTGGGCTCAAGGTGAGGGGTGCTATCTGTGNNNGAGGGACATGGTTAATGGAATTGTCTCACACAGAAATCGCACCCGTCACC
F-342_G17	GTGAAACTGGGCTCAAGGTGAGGGGTGCTATCTGTGANNNAGGGACATGGTTAATGGAATTGTCTCACACAGAAATCGCACCCGTCACC
F-342_G18	GTGAAACTGGGCTCAAGGTGAGGGGTGCTATCTGTGATNNNGGGACATGGTTAATGGAATTGTCTCACACAGAAATCGCACCCGTCACC
F-342_G19	GTGAAACTGGGCTCAAGGTGAGGGGTGCTATCTGTGATTNNNGGACATGGTTAATGGAATTGTCTCACACAGAAATCGCACCCGTCACC
F-342_G20	GTGAAACTGGGCTCAAGGTGAGGGGTGCTATCTGTGATTGNNNGACATGGTTAATGGAATTGTCNNACACAGAAATCGCACCCGTCACC
F-342_G21	GTGAAACTGGGCTCAAGGTGAGGGGTGCTATCTGTGATTGANNNACATGGTTAATGGAATTGTNNNACACAGAAATCGCACCCGTCACC
R-342_G16-21	TGGTGATAAGTAGGCCAAGGTGACGGGTGCGATTTCTG
F-T7-342	TAATACGACTCACTATAGGGGTGAAACTGGGCTCAAGGTG
R-342	TGGGGTGGTGATAAGTAGGCCAAGG

Supplementary Table 4. The PCR primers for arti2 and arti3 RNA preparation

The underlined sequence is T7 promoter.

Substrates name	PCR primers (5'-3')	DNA sequences encoding for RNA substrates (5'-3')
Arti2	**F-arti2**<br>TAATACGACTCACTATAGGGTAAACACAGCATTCCGTTATGTAGCATTTCT<br>TGATTGTGAGCGAGGTGCAAGAAGAACTCACGATCAAG<br>**R-arti2**<br>GTCTGCGGCAACACTCCGTTATGTAGCATTTCTTGATCGTGAGTTCTTCTT<br>GCACCTCG	GGGTAAACACAGCATTCCGTTATGTAGCATTTCTTGATTGTG<br>AGCGAGGTGCAAGAAGAACTCACGATCAAGAAATGCTACAT<br>AACGGAGTGTTGCCGCAGAC
Arti2_midW789	**F-arti2_midW789**<br>TAATACGACTCACTATAGGGTAAACACAGCATTCCGTTATGTAGCGGGTC<br>TTGATTGTGAGCGAGGTGCAAGAAGAACTCACGATCAAG<br>**R-arti2_ midW789**<br>GTCTGCGGCAACACTCCGTTATGTAGCAAATCTTGATCGTGAGTTCTTCTT<br>GCACCTCG	GGGTAAACACAGCATTCCGTTATGTAGCGGGTCTTGATTGT<br>GAGCGAGGTGCAAGAAGAACTCACGATCAAGATTTGCTACA<br>TAACGGAGTGTTGCCGCAGAC
Arti2_midW 8910	**F-arti2_ midW8910**<br>TAATACGACTCACTATAGGGTAAACACAGCATTCCGTTATGTAGCAGGGC<br>TTGATTGTGAGCGAGGTGCAAGAAGAACTCACGATCAAG<br>**R-arti2_ midW8910**<br>GTCTGCGGCAACACTCCGTTATGTAGCAAAACTTGATCGTGAGTTCTTCTT<br>GCACCTCG	GGGTAAACACAGCATTCCGTTATGTAGCAGGGCTTGATTGT<br>GAGCGAGGTGCAAGAAGAACTCACGATCAAGTTTTGCTACA<br>TAACGGAGTGTTGCCGCAGAC
Arti2_midW 91011	**F-arti2_midW91011**<br>TAATACGACTCACTATAGGGTAAACACAGCATTCCGTTATGTAGCATGGG<br>TTGATTGTGAGCGAGGTGCAAGAAGAACTCACGATCAAT<br>**R-arti2_ midW91011**<br>GTCTGCGGCAACACTCCGTTATGTAGCATAAATTGATCGTGAGTTCTTCTT<br>GCACCTCG	GGGTAAACACAGCATTCCGTTATGTAGCATGGGTTGATTGT<br>GAGCGAGGTGCAAGAAGAACTCACGATCAATTTATGCTACA<br>TAACGGAGTGTTGCCGCAGAC
Arti2_midW<br>101112	**F-arti2_midW 101112**<br>TAATACGACTCACTATAGGGTAAACACAGCATTCCGTTATGTAGCATTGG<br>GTGATTGTGAGCGAGGTGCAAGAAGAACTCACGATCATT<br>**R-arti2_midW 101112**<br>GTCTGCGGCAACACTCCGTTATGTAGCATTAAATGATCGTGAGTTCTTCTT<br>GCACCTCG	GGGTAAACACAGCATTCCGTTATGTAGCATTGGGTGATTGT<br>GAGCGAGGTGCAAGAAGAACTCACGATCATTTAATGCTACA<br>TAACGGAGTGTTGCCGCAGAC

Arti2_midW 111213	**F-arti2_midW 111213**<br>TAATACGACTCACTATAGGGTAAACACAGCATTCCGTTATGTAGCATTTGG<br>GGATTGTGAGCGAGGTGCAAGAAGAACTCACGATCTTT<br>**R-arti2_midW 111213**<br>GTCTGCGGCAACACTCCGTTATGTAGCATTTAAAGATCGTGAGTTCTTCTT<br>GCACCTCG	GGGTAAACACAGCATTCCGTTATGTAGCATTTGGGGATTGT<br>GAGCGAGGTGCAAGAAGAACTCACGATCTTTAAATGCTACA<br>TAACGGAGTGTTGCCGCAGAC
Arti2_midW 8910UG	**F-arti2**<br>TAATACGACTCACTATAGGGTAAACACAGCATTCCGTTATGTAGCATTTCT<br>TGATTGTGAGCGAGGTGCAAGAAGAACTCACGATCAAG<br>**R-arti2_midW8910UG**<br>GTCTGCGGCAACACTCCGTTATGTAGCACCCCTTGATCGTGAGTTCTTCT<br>TGCACCTCG	GGGTAAACACAGCATTCCGTTATGTAGCATTTTTTGATTGTG<br>AGCGAGGTGCAAGAAGAACTCACGATCAAGGGGTGCTACAT<br>AACGGAGTGTTGCCGCAGAC
Arti2_midW 91011UG	**F-arti2_midW 91011UG**<br>TAATACGACTCACTATAGGGTAAACACAGCATTCCGTTATGTAGCATTTTT<br>TGATTGTGAGCGAGGTGCAAGAAGAACTCACGATCAAG<br>**R-arti2_midW 91011UG**<br>GTCTGCGGCAACACTCCGTTATGTAGCATCCCTTGATCGTGAGTTCTTCT<br>TGCACCTCG	GGGTAAACACAGCATTCCGTTATGTAGCATTTTTTGATTGTG<br>AGCGAGGTGCAAGAAGAACTCACGATCAAGGGATGCTACAT<br>AACGGAGTGTTGCCGCAGAC
Arti2_midW 101112UG	**F-arti2_midW 91011UG**<br>TAATACGACTCACTATAGGGTAAACACAGCATTCCGTTATGTAGCATTTTT<br>TGATTGTGAGCGAGGTGCAAGAAGAACTCACGATCAAG<br>**R-arti2_midW101112UG**<br>GTCTGCGGCAACACTCCGTTATGTAGCATTCCCTGATCGTGAGTTCTTCT<br>TGCACCTCG	GGGTAAACACAGCATTCCGTTATGTAGCATTTTTTGATTGTG<br>AGCGAGGTGCAAGAAGAACTCACGATCAGGGAATGCTACAT<br>AACGGAGTGTTGCCGCAGAC
Arti2_midW10	**F-arti2_midW10**<br>TAATACGACTCACTATAGGGTAAACACAGCATTCCGTTATGTAGCATTGCT<br>TGATTGTGAGCGAGGTGCAAGAAGAACTCACGATCAAG<br>**R-arti2_midW10**<br>GTCTGCGGCAACACTCCGTTATGTAGCATTACTTGATCGTGAGTTCTTCTT<br>GCACCTC	GGGTAAACACAGCATTCCGTTATGTAGCATTGCTTGATTGTG<br>AGCGAGGTGCAAGAAGAACTCACGATCAAGTAATGCTACAT<br>AACGGAGTGTTGCCGCAGAC
Arti2_midW11	**F-arti2_midW11**<br>TAATACGACTCACTATAGGGTAAACACAGCATTCCGTTATGTAGCATTTGT<br>TGATTGTGAGCGAGGTGCAAGAAGAACTCACGATCAAT<br>**R-arti2_midW11**<br>GTCTGCGGCAACACTCCGTTATGTAGCATTTATTGATCGTGAGTTCTTCTT<br>GCACCTCG	GGGTAAACACAGCATTCCGTTATGTAGCATTTGTTGATTGTG<br>AGCGAGGTGCAAGAAGAACTCACGATCAATAAATGCTACAT<br>AACGGAGTGTTGCCGCAGAC
Arti2_midW1011	**F-arti2_midW1011**<br>TAATACGACTCACTATAGGGTAAACACAGCATTCCGTTATGTAGCATTGGT<br>TGATTGTGAGCGAGGTGCAAGAAGAACTCACGATCAAT<br>**R-arti2_midW1011**<br>GTCTGCGGCAACACTCCGTTATGTAGCATTAATTGATCGTGAGTTCTTCTT<br>GCACCTCG	GGGTAAACACAGCATTCCGTTATGTAGCATTGGTTGATTGTG<br>AGCGAGGTGCAAGAAGAACTCACGATCAATTAATGCTACAT<br>AACGGAGTGTTGCCGCAGAC
Arti2_midM789	**F-arti2**<br>TAATACGACTCACTATAGGGTAAACACAGCATTCCGTTATGTAGCATTTCT<br>TGATTGTGAGCGAGGTGCAAGAAGAACTCACGATCAAG<br>**R-arti2_midM789**<br>GTCTGCGGCAACACTCCGTTATGTAGCTGGTCTTGATCGTGAGTTCTTCT<br>TGCACCTCG	GGGTAAACACAGCATTCCGTTATGTAGCATTTCTTGATTGTG<br>AGCGAGGTGCAAGAAGAACTCACGATCAAG**A**CCAGCTACAT<br>AACGGAGTGTTGCCGCAGAC
Arti2_ midM8910	**F-arti2**<br>TAATACGACTCACTATAGGGTAAACACAGCATTCCGTTATGTAGCATTTCT<br>TGATTGTGAGCGAGGTGCAAGAAGAACTCACGATCAAG<br>**R-arti2_midM8910**<br>GTCTGCGGCAACACTCCGTTATGTAGCAGGGCTTGATCGTGAGTTCTTCT<br>TGCACCTCG	GGGTAAACACAGCATTCCGTTATGTAGCATTTCTTGATTGTG<br>AGCGAGGTGCAAGAAGAACTCACGATCAAGCCCTGCTACAT<br>AACGGAGTGTTGCCGCAGAC
Arti2_ midM91011	**F-arti2_11C**<br>TAATACGACTCACTATAGGGTAAACACAGCATTCCGTTATGTAGCATTTCT<br>TGATTGTGAGCGAGGTGCAAGAAGAACTCACGATCAAC<br>**R-arti2_midM91011**<br>GTCTGCGGCAACACTCCGTTATGTAGCATGGGTTGATCGTGAGTTCTTCT<br>TGCACCTCG	GGGTAAACACAGCATTCCGTTATGTAGCATTTCTTGATTGTG<br>AGCGAGGTGCAAGAAGAACTCACGATCAACCCATGCTACAT<br>AACGGAGTGTTGCCGCAGAC
Arti2_midM101112	**F-arti2_1112CC**<br>TAATACGACTCACTATAGGGTAAACACAGCATTCCGTTATGTAGCATTTCT<br>TGATTGTGAGCGAGGTGCAAGAAGAACTCACGATCACC<br>**R-arti2_midM101112**<br>GTCTGCGGCAACACTCCGTTATGTAGCATTGGGTGATCGTGAGTTCTTCT<br>TGCACCTCG	GGGTAAACACAGCATTCCGTTATGTAGCATTTCTTGATTGTG<br>AGCGAGGTGCAAGAAGAACTCACGATCACCCAATGCTACAT<br>AACGGAGTGTTGCCGCAGAC
Arti2_ midM 111213	**F-arti2_111213CCC**<br>TAATACGACTCACTATAGGGTAAACACAGCATTCCGTTATGTAGCATTTCT<br>TGATTGTGAGCGAGGTGCAAGAAGAACTCACGATCCCC<br>**R-arti2_ midM111213**<br>GTCTGCGGCAACACTCCGTTATGTAGCATTTGGGGATCGTGAGTTCTTCT<br>TGCACCTCG	GGGTAAACACAGCATTCCGTTATGTAGCATTTCTTGATTGTG<br>AGCGAGGTGCAAGAAGAACTCACGATCCCCAAATGCTACAT<br>AACGGAGTGTTGCCGCAGAC
Arti2_midM 10	**F-arti2**<br>TAATACGACTCACTATAGGGTAAACACAGCATTCCGTTATGTAGCATTTCT<br>TGATTGTGAGCGAGGTGCAAGAAGAACTCACGATCAAG<br>**R-arti2_midM10**<br>GTCTGCGGCAACACTCCGTTATGTAGCATTGCTTGATCGTGAGTTCTTCTT<br>GCACCTCG	GGGTAAACACAGCATTCCGTTATGTAGCATTTCTTGATTGTG<br>AGCGAGGTGCAAGAAGAACTCACGATCAAGCAATGCTACAT<br>AACGGAGTGTTGCCGCAGAC
Arti2_ midM11	**F-arti2_midM 11**<br>TAATACGACTCACTATAGGGTAAACACAGCATTCCGTTATGTAGCATTTCT<br>TGATTGTGAGCGAGGTGCAAGAAGAACTCACGATCAAC<br>**R-arti2_midM11**<br>GTCTGCGGCAACACTCCGTTATGTAGCATTTGTTGATCGTGAGTTCTTCTT<br>GCACCTCG	GGGTAAACACAGCATTCCGTTATGTAGCATTTCTTGATTGTG<br>AGCGAGGTGCAAGAAGAACTCACGATCAACAAATGCTACAT<br>AACGGAGTGTTGCCGCAGAC
Arti2_ midM1011	**F-arti2_midM 11**<br>TAATACGACTCACTATAGGGTAAACACAGCATTCCGTTATGTAGCATTTCT<br>TGATTGTGAGCGAGGTGCAAGAAGAACTCACGATCAAC<br>**R-arti2_midM1011**<br>GTCTGCGGCAACACTCCGTTATGTAGCATTGGTTGATCGTGAGTTCTTCT<br>TGCACCTCG	GGGTAAACACAGCATTCCGTTATGTAGCATTTCTTGATTGTG<br>AGCGAGGTGCAAGAAGAACTCACGATCAACCAATGCTACAT<br>AACGGAGTGTTGCCGCAGAC

Arti2_ midM7	**F-arti2**<br>TAATACGACTCACTATAGGGTAAACACAGCATTCCGTTATGTAGCATTTCT<br>TGATTGTGAGCGAGGTGCAAGAAGAACTCACGATCAAG<br>**R-arti2_midM7**<br>GTCTGCGGCAACACTCCGTTATGTAGCTTTTCTTGATCGTGAGTTCTTCTT<br>GCACCTCG	GGGTAAACACAGCATTCCGTTATGTAGCATTTCTTGATTGTG<br>AGCGAGGTGCAAGAAGAACTCACGATCAAGAAAAGCTACAT<br>AACGGAGTGTTGCCGCAGAC
Arti2_ midM8	**F-arti2**<br>TAATACGACTCACTATAGGGTAAACACAGCATTCCGTTATGTAGCATTTCT<br>TGATTGTGAGCGAGGTGCAAGAAGAACTCACGATCAAG<br>**R-arti2_midM8**<br>GTCTGCGGCAACACTCCGTTATGTAGCAGTTCTTGATCGTGAGTTCTTCTT<br>GCACCTCG	GGGTAAACACAGCATTCCGTTATGTAGCATTTCTTGATTGTG<br>AGCGAGGTGCAAGAAGAACTCACGATCAAGAACTGCTACAT<br>AACGGAGTGTTGCCGCAGAC
Arti2_ midM78	**F-arti2**<br>TAATACGACTCACTATAGGGTAAACACAGCATTCCGTTATGTAGCATTTCT<br>TGATTGTGAGCGAGGTGCAAGAAGAACTCACGATCAAG<br>**R-arti2_midM78**<br>GTCTGCGGCAACACTCCGTTATGTAGCTGTTCTTGATCGTGAGTTCTTCTT<br>GCACCTCG	GGGTAAACACAGCATTCCGTTATGTAGCATTTCTTGATTGTG<br>AGCGAGGTGCAAGAAGAACTCACGATCAAGAACAGCTACAT<br>AACGGAGTGTTGCCGCAGAC
Arti2_ midM9	**F-arti2**<br>TAATACGACTCACTATAGGGTAAACACAGCATTCCGTTATGTAGCATTTCT<br>TGATTGTGAGCGAGGTGCAAGAAGAACTCACGATCAAG<br>**R-arti2_midM9**<br>GTCTGCGGCAACACTCCGTTATGTAGCATGTCTTGATCGTGAGTTCTTCTT<br>GCACCTCG	GGGTAAACACAGCATTCCGTTATGTAGCATTTCTTGATTGTG<br>AGCGAGGTGCAAGAAGAACTCACGATCAAGACATGCTACAT<br>AACGGAGTGTTGCCGCAGAC
Arti2_ midM89	**F-arti2**<br>TAATACGACTCACTATAGGGTAAACACAGCATTCCGTTATGTAGCATTTCT<br>TGATTGTGAGCGAGGTGCAAGAAGAACTCACGATCAAG<br>**R-arti2_midM89**<br>GTCTGCGGCAACACTCCGTTATGTAGCAGGTCTTGATCGTGAGTTCTTCT<br>TGCACCTCG	GGGTAAACACAGCATTCCGTTATGTAGCATTTCTTGATTGTG<br>AGCGAGGTGCAAGAAGAACTCACGATCAAGACCTGCTACAT<br>AACGGAGTGTTGCCGCAGAC
Arti2_-3-2-1MMM	**F-arti2**<br>TAATACGACTCACTATAGGGTAAACACAGCATTCCGTTATGTAGCATTTCT<br>TGATTGTGAGCGAGGTGCAAGAAGAACTCACGATCAAG<br>**R-arti2_-3-2-1MMM**<br>GTCTGCGGCAACACTCCGGGGTGTAGCATTTCTTGATCGTGAGTTCTTCT<br>TGCACCTCG	GGGTAAACACAGCATTCCGTTATGTAGCATTTCTTGATTGTG<br>AGCGAGGTGCAAGAAGAACTCACGATCAAGAAATGCTACAC<br>CCCGGAGTGTTGCCGCAGAC
Arti2_-2-11MMM	**F-arti2**<br>TAATACGACTCACTATAGGGTAAACACAGCATTCCGTTATGTAGCATTTCT<br>TGATTGTGAGCGAGGTGCAAGAAGAACTCACGATCAAG<br>**R-arti2_-2-11MMM**<br>GTCTGCGGCAACACTCCGTGGGGTAGCATTTCTTGATCGTGAGTTCTTCT<br>TGCACCTCG	GGGTAAACACAGCATTCCGTTATGTAGCATTTCTTGATTGTG<br>AGCGAGGTGCAAGAAGAACTCACGATCAAGAAATGCTACCC<br>CACGGAGTGTTGCCGCAGAC
Arti2_-112MMM	**F-arti2**<br>TAATACGACTCACTATAGGGTAAACACAGCATTCCGTTATGTAGCATTTCT<br>TGATTGTGAGCGAGGTGCAAGAAGAACTCACGATCAAG<br>**R-arti2_-112MMM**<br>GTCTGCGGCAACACTCCGTTGGTTAGCATTTCTTGATCGTGAGTTCTTCTT<br>GCACCTCG	GGGTAAACACAGCATTCCGTTATGTAGCATTTCTTGATTGTG<br>AGCGAGGTGCAAGAAGAACTCACGATCAAGAAATGCTAACC<br>AACGGAGTGTTGCCGCAGAC
Arti2_123MMM	**F-arti2**<br>TAATACGACTCACTATAGGGTAAACACAGCATTCCGTTATGTAGCATTTCT<br>TGATTGTGAGCGAGGTGCAAGAAGAACTCACGATCAAG<br>**R-arti2_1MMM**<br>GTCTGCGGCAACACTCCGTTAATAAGCATTTCTTGATCGTGAGTTCTTCTT<br>GCACCTCG	GGGTAAACACAGCATTCCGTTATGTAGCATTTCTTGATTGTG<br>AGCGAGGTGCAAGAAGAACTCACGATCAAGAAATGCTTATT<br>AACGGAGTGTTGCCGCAGAC
Arti2_-1M	**F-arti2**<br>TAATACGACTCACTATAGGGTAAACACAGCATTCCGTTATGTAGCATTTCT<br>TGATTGTGAGCGAGGTGCAAGAAGAACTCACGATCAAG<br>**R-arti2_-1M**<br>GTCTGCGGCAACACTCCGTTGTGTAGCATTTCTTGATCGTGAGTTCTTCTT<br>GCACCTCG	GGGTAAACACAGCATTCCGTTATGTAGCATTTCTTGATTGTG<br>AGCGAGGTGCAAGAAGAACTCACGATCAAGAAATGCTACAC<br>AACGGAGTGTTGCCGCAGAC
Arti2_-2M	**F-arti2**<br>TAATACGACTCACTATAGGGTAAACACAGCATTCCGTTATGTAGCATTTCT<br>TGATTGTGAGCGAGGTGCAAGAAGAACTCACGATCAAG<br>**R-arti2_-2M**<br>GTCTGCGGCAACACTCCGTGATGTAGCATTTCTTGATCGTGAGTTCTTCTT<br>GCACCTCG	GGGTAAACACAGCATTCCGTTATGTAGCATTTCTTGATTGTG<br>AGCGAGGTGCAAGAAGAACTCACGATCAAGAAATGCTACAT<br>CACGGAGTGTTGCCGCAGAC
Arti2_-3M	**F-arti2**<br>TAATACGACTCACTATAGGGTAAACACAGCATTCCGTTATGTAGCATTTCT<br>TGATTGTGAGCGAGGTGCAAGAAGAACTCACGATCAAG<br>**R-arti2_-3M**<br>GTCTGCGGCAACACTCCGGTATGTAGCATTTCTTGATCGTGAGTTCTTCTT<br>GCACCTCG	GGGTAAACACAGCATTCCGTTATGTAGCATTTCTTGATTGTG<br>AGCGAGGTGCAAGAAGAACTCACGATCAAGAAATGCTACAT<br>ACCGGAGTGTTGCCGCAGAC
Arti2_-2-1MM	**F-arti2**<br>TAATACGACTCACTATAGGGTAAACACAGCATTCCGTTATGTAGCATTTCT<br>TGATTGTGAGCGAGGTGCAAGAAGAACTCACGATCAAG<br>**R-arti2_-2-1MM**<br>GTCTGCGGCAACACTCCGTGGTGTAGCATTTCTTGATCGTGAGTTCTTCT<br>TGCACCTCG	GGGTAAACACAGCATTCCGTTATGTAGCATTTCTTGATTGTG<br>AGCGAGGTGCAAGAAGAACTCACGATCAAGAAATGCTACAC<br>CACGGAGTGTTGCCGCAGAC
Arti2_-3-2MM	**F-arti2**<br>TAATACGACTCACTATAGGGTAAACACAGCATTCCGTTATGTAGCATTTCT<br>TGATTGTGAGCGAGGTGCAAGAAGAACTCACGATCAAG<br>**R-arti2_-3-2MM**<br>GTCTGCGGCAACACTCCGGGATGTAGCATTTCTTGATCGTGAGTTCTTCT<br>TGCACCTCG	GGGTAAACACAGCATTCCGTTATGTAGCATTTCTTGATTGTG<br>AGCGAGGTGCAAGAAGAACTCACGATCAAGAAATGCTACAT<br>CCCGGAGTGTTGCCGCAGAC
Arti3	**F-arti3**<br>TAATACGACTCACTATAGGGTAAACACAGCATTCCGTTATGTAGCATTTCT<br>TGGTTGTGAGCGAGGTGCAAGAAGAACTCACGATCAAG<br>**R-arti3**<br>GTCTGCGGCAACACTCCGTTATGTAGCATTCCTTGATCGTGAGTTCTTCTT<br>GCACCTCG	GGGTAAACACAGCATTCCGTTATGTAGCATTTCTTGGTTGTG<br>AGCGAGGTGCAAGAAGAACTCACGATCAAGGAATGCTACAT<br>AACGGAGTGTTGCCGCAGAC

Arti3_seedW4	**F-arti3_seedW4**<br>TAATACGACTCACTATAGGGTAAACACAGCATTCCGTTATGTGGCATTTCT<br>TGGTTGTGAGCGAGGTGCAAGAAGAACTCACGATCAAG<br>**R-arti3**<br>GTCTGCGGCAACACTCCGTTATGTAGCATTCCTTGATCGTGAGTTCTTCTT<br>GCACCTCG	GGGTAAACACAGCATTCCGTTATGTGGCATTTCTTGGTTGTG<br>AGCGAGGTGCAAGAAGAACTCACGATCAAGGAATGCTACAT<br>AACGGAGTGTTGCCGCAGAC
Arti3_seedW5	**F-arti3**<br>TAATACGACTCACTATAGGGTAAACACAGCATTCCGTTATGTAGCATTTCT<br>TGGTTGTGAGCGAGGTGCAAGAAGAACTCACGATCAAG<br>**R-arti3_seedW5**<br>GTCTGCGGCAACACTCCGTTATGTAACATTCCTTGATCGTGAGTTCTTCTT<br>GCACCTCG	GGGTAAACACAGCATTCCGTTATGTAGCATTTCTTGGTTGTG<br>AGCGAGGTGCAAGAAGAACTCACGATCAAGGAATGTTACAT<br>AACGGAGTGTTGCCGCAGAC
Arti3_seedW6	**F-arti3_seedW6**<br>TAATACGACTCACTATAGGGTAAACACAGCATTCCGTTATGTAGGATTTCT<br>TGGTTGTGAGCGAGGTGCAAGAAGAACTCACGATCAAG<br>**R-arti3_seedW6**<br>GTCTGCGGCAACACTCCGTTATGTAGAATTCCTTGATCGTGAGTTCTTCTT<br>GCACCTCG	GGGTAAACACAGCATTCCGTTATGTAGGATTTCTTGGTTGTG<br>AGCGAGGTGCAAGAAGAACTCACGATCAAGGAATTCTACAT<br>AACGGAGTGTTGCCGCAGAC
Arti3_seedW46	**F-arti3_seedW46**<br>TAATACGACTCACTATAGGGTAAACACAGCATTCCGTTATGTGGGATTTCT<br>TGGTTGTGAGCGAGGTGCAAGAAGAACTCACGATCAAG<br>**R-arti3_seedW6**<br>GTCTGCGGCAACACTCCGTTATGTAGAATTCCTTGATCGTGAGTTCTTCTT<br>GCACCTCG	GGGTAAACACAGCATTCCGTTATGTGGGATTTCTTGGTTGT<br>GAGCGAGGTGCAAGAAGAACTCACGATCAAGGAATTCTACA<br>TAACGGAGTGTTGCCGCAGAC
Arti3_seedW56	**F-arti3_seedW6**<br>TAATACGACTCACTATAGGGTAAACACAGCATTCCGTTATGTAGGATTTCT<br>TGGTTGTGAGCGAGGTGCAAGAAGAACTCACGATCAAG<br>**R-arti3_seedW56**<br>GTCTGCGGCAACACTCCGTTATGTAAAATTCCTTGATCGTGAGTTCTTCTT<br>GCACCTCG	GGGTAAACACAGCATTCCGTTATGTAGGATTTCTTGGTTGTG<br>AGCGAGGTGCAAGAAGAACTCACGATCAAGGAATTTTACAT<br>AACGGAGTGTTGCCGCAGAC
Arti3_46WM	**F-arti3_seedW4**<br>TAATACGACTCACTATAGGGTAAACACAGCATTCCGTTATGTGGCATTTCT<br>TGGTTGTGAGCGAGGTGCAAGAAGAACTCACGATCAAG<br>**R-arti3_seedM6**<br>GTCTGCGGCAACACTCCGTTATGTAGTATTCCTTGATCGTGAGTTCTTCTT<br>GCACCTCG	GGGTAAACACAGCATTCCGTTATGTGGCATTTCTTGGTTGTG<br>AGCGAGGTGCAAGAAGAACTCACGATCAAGGAATACTACAT<br>AACGGAGTGTTGCCGCAGAC
Arti3_456WWM	**F-arti3_seedW4**<br>TAATACGACTCACTATAGGGTAAACACAGCATTCCGTTATGTGGCATTTCT<br>TGGTTGTGAGCGAGGTGCAAGAAGAACTCACGATCAAG<br>**R-arti3_seedM56**<br>GTCTGCGGCAACACTCCGTTATGTAATATTCCTTGATCGTGAGTTCTTCTT<br>GCACCTCG	GGGTAAACACAGCATTCCGTTATGTGGCATTTCTTGGTTGTG<br>AGCGAGGTGCAAGAAGAACTCACGATCAAGGAATATTACAT<br>AACGGAGTGTTGCCGCAGAC
Arti3_56WM	**F-arti3**<br>TAATACGACTCACTATAGGGTAAACACAGCATTCCGTTATGTAGCATTTCT<br>TGGTTGTGAGCGAGGTGCAAGAAGAACTCACGATCAAG<br>**R-arti3_5W6M**<br>GTCTGCGGCAACACTCCGTTATGTAATATTCCTTGATCGTGAGTTCTTCTT<br>GCACCTCG	GGGTAAACACAGCATTCCGTTATGTAGCATTTCTTGGTTGTG<br>AGCGAGGTGCAAGAAGAACTCACGATCAAGGAATATTACAT<br>AACGGAGTGTTGCCGCAGAC
Arti3_seedM4	**F-arti3**<br>TAATACGACTCACTATAGGGTAAACACAGCATTCCGTTATGTAGCATTTCT<br>TGGTTGTGAGCGAGGTGCAAGAAGAACTCACGATCAAG<br>**R-arti3_seedM4**<br>GTCTGCGGCAACACTCCGTTATGTTGCATTCCTTGATCGTGAGTTCTTCTT<br>GCACCTCG	GGGTAAACACAGCATTCCGTTATGTAGCATTTCTTGGTTGTG<br>AGCGAGGTGCAAGAAGAACTCACGATCAAGGAATGCAACAT<br>AACGGAGTGTTGCCGCAGAC
Arti3_seedM5	**F-arti3**<br>TAATACGACTCACTATAGGGTAAACACAGCATTCCGTTATGTAGCATTTCT<br>TGGTTGTGAGCGAGGTGCAAGAAGAACTCACGATCAAG<br>**R-arti3_seedM5**<br>GTCTGCGGCAACACTCCGTTATGTATCATTCCTTGATCGTGAGTTCTTCTT<br>GCACCTCG	GGGTAAACACAGCATTCCGTTATGTAGCATTTCTTGGTTGTG<br>AGCGAGGTGCAAGAAGAACTCACGATCAAGGAATGATACAT<br>AACGGAGTGTTGCCGCAGAC
Arti3_seedM6	**F-arti3**<br>TAATACGACTCACTATAGGGTAAACACAGCATTCCGTTATGTAGCATTTCT<br>TGGTTGTGAGCGAGGTGCAAGAAGAACTCACGATCAAG<br>**R-arti3_seedM6**<br>GTCTGCGGCAACACTCCGTTATGTAGTATTCCTTGATCGTGAGTTCTTCTT<br>GCACCTCG	GGGTAAACACAGCATTCCGTTATGTAGCATTTCTTGGTTGTG<br>AGCGAGGTGCAAGAAGAACTCACGATCAAGGAATACTACAT<br>AACGGAGTGTTGCCGCAGAC
Arti3_seedM46	**F-arti3**<br>TAATACGACTCACTATAGGGTAAACACAGCATTCCGTTATGTAGCATTTCT<br>TGGTTGTGAGCGAGGTGCAAGAAGAACTCACGATCAAG<br>**R-arti3_seedM46**<br>GTCTGCGGCAACACTCCGTTATGTTGTATTCCTTGATCGTGAGTTCTTCTT<br>GCACCTCG	GGGTAAACACAGCATTCCGTTATGTAGCATTTCTTGGTTGTG<br>AGCGAGGTGCAAGAAGAACTCACGATCAAGGAATACAACAT<br>AACGGAGTGTTGCCGCAGAC
Arti3_seedM56	**F-arti3**<br>TAATACGACTCACTATAGGGTAAACACAGCATTCCGTTATGTAGCATTTCT<br>TGGTTGTGAGCGAGGTGCAAGAAGAACTCACGATCAAG<br>**R-arti3_seedM56**<br>GTCTGCGGCAACACTCCGTTATGTATTATTCCTTGATCGTGAGTTCTTCTT<br>GCACCTCG	GGGTAAACACAGCATTCCGTTATGTAGCATTTCTTGGTTGTG<br>AGCGAGGTGCAAGAAGAACTCACGATCAAGGAATAATACAT<br>AACGGAGTGTTGCCGCAGAC
Arti3_seedW7	**F-arti3_seedW7**<br>TAATACGACTCACTATAGGGTAAACACAGCATTCCGTTATGTAGCGTTTCT<br>TGGTTGTGAGCGAGGTGCAAGAAGAACTCACGA<br>**R-arti3**<br>GTCTGCGGCAACACTCCGTTATGTAGCATTCCTTGATCGTGAGTTCTTCTT<br>GCACCTCG	GGGTAAACACAGCATTCCGTTATGTAGCGTTTCTTGGTTGTG<br>AGCGAGGTGCAAGAAGAACTCACGATCAAGGAATGCTACAT<br>AACGGAGTGTTGCCGCAGAC
Arti3_seedW8	**F-arti3_seedW8**<br>TAATACGACTCACTATAGGGTAAACACAGCATTCCGTTATGTAGCAGTTCT<br>TGGTTGTGAGCGAGGTGCAAGAAGAACTCACGA<br>**R-arti3_seedW8**<br>GTCTGCGGCAACACTCCGTTATGTAGCAATCCTTGATCGTGAGTTCTTCTT<br>GCACCTCG	GGGTAAACACAGCATTCCGTTATGTAGCAGTTCTTGGTTGT<br>GAGCGAGGTGCAAGAAGAACTCACGATCAAGGATTGCTACA<br>TAACGGAGTGTTGCCGCAGAC

Arti3_seedW67	**F-arti3_seedW67**<br>TAATACGACTCACTATAGGGTAAACACAGCATTCCGTTATGTAGGGTTTCT TGGTTGTGAGCGAGGTGCAAGAAGAACTCACGATCAAG<br>**R-arti3_seedW67**<br>GTCTGCGGCAACACTCCGTTATGTAGAATTCCTTGATCGTGAGTTCTTCTT GCACCTCG	GGGTAAACACAGCATTCCGTTATGTAGGGTTTCTTGGTTGT GAGCGAGGTGCAAGAAGAACTCACGATCAAGGAATTCTACA TAACGGAGTGTTGCCGCAGAC
Arti3_seedW678	**F-arti3_seedW678**<br>TAATACGACTCACTATAGGGTAAACACAGCATTCCGTTATGTAGGGGTTCT TGGTTGTGAGCGAGGTGCAAGAAGAACTCACGATCAAG<br>**R-arti3_seedW678**<br>GTCTGCGGCAACACTCCGTTATGTAGAAATCCTTGATCGTGAGTTCTTCTT GCACCTCG	GGGTAAACACAGCATTCCGTTATGTAGGGGTTCTTGGTTGT GAGCGAGGTGCAAGAAGAACTCACGATCAAGGATTTCTACA TAACGGAGTGTTGCCGCAGAC
Arti3_seedW78	**F-arti3_seedW78**<br>TAATACGACTCACTATAGGGTAAACACAGCATTCCGTTATGTAGCGGTTCT TGGTTGTGAGCGAGGTGCAAGAAGAACTCACGATCAAG<br>**R-arti3_seedW78**<br>GTCTGCGGCAACACTCCGTTATGTAGCAATCCTTGATCGTGAGTTCTTCTT GCACCTCG	GGGTAAACACAGCATTCCGTTATGTAGCGGTTCTTGGTTGT GAGCGAGGTGCAAGAAGAACTCACGATCAAGGATTGCTACA TAACGGAGTGTTGCCGCAGAC
Arti3_seedM567	**F-arti3**<br>TAATACGACTCACTATAGGGTAAACACAGCATTCCGTTATGTAGCATTTCT TGGTTGTGAGCGAGGTGCAAGAAGAACTCACGATCAAG<br>**R-arti3_seedM567**<br>GTCTGCGGCAACACTCCGTTATGTATTTTTCCTTGATCGTGAGTTCTTCTT GCACCTCG	GGGTAAACACAGCATTCCGTTATGTAGCATTTCTTGGTTGTG AGCGAGGTGCAAGAAGAACTCACGATCAAGGAAAAATACAT AACGGAGTGTTGCCGCAGAC
Arti3_seedM67	**F-arti3**<br>TAATACGACTCACTATAGGGTAAACACAGCATTCCGTTATGTAGCATTTCT TGGTTGTGAGCGAGGTGCAAGAAGAACTCACGATCAAG<br>**R-arti3_seedM67**<br>GTCTGCGGCAACACTCCGTTATGTAGTTTTCCTTGATCGTGAGTTCTTCTT GCACCTCG	GGGTAAACACAGCATTCCGTTATGTAGCATTTCTTGGTTGTG AGCGAGGTGCAAGAAGAACTCACGATCAAGGAAAACTACAT AACGGAGTGTTGCCGCAGAC
Arti3_seedM678	**F-arti3**<br>TAATACGACTCACTATAGGGTAAACACAGCATTCCGTTATGTAGCATTTCT TGGTTGTGAGCGAGGTGCAAGAAGAACTCACGATCAAG<br>**R-arti3_seedM678**<br>GTCTGCGGCAACACTCCGTTATGTAGTTGTCCTTGATCGTGAGTTCTTCTT GCACCTCG	GGGTAAACACAGCATTCCGTTATGTAGCATTTCTTGGTTGTG AGCGAGGTGCAAGAAGAACTCACGATCAAGGACAACTACAT AACGGAGTGTTGCCGCAGAC
Arti3_seedM78	**F-arti3**<br>TAATACGACTCACTATAGGGTAAACACAGCATTCCGTTATGTAGCATTTCT TGGTTGTGAGCGAGGTGCAAGAAGAACTCACGATCAAG<br>**R-aarti2_seedM78**<br>GTCTGCGGCAACACTCCGTTATGTAGCTGTCCTTGATCGTGAGTTCTTCT TGCACCTCG	GGGTAAACACAGCATTCCGTTATGTAGCATTTCTTGGTTGTG AGCGAGGTGCAAGAAGAACTCACGATCAAGGACAGCTACAT AACGGAGTGTTGCCGCAGAC
Arti3_seedM7	**F-arti3**<br>TAATACGACTCACTATAGGGTAAACACAGCATTCCGTTATGTAGCATTTCT TGGTTGTGAGCGAGGTGCAAGAAGAACTCACGATCAAG<br>**R-arti3_seedM7**<br>GTCTGCGGCAACACTCCGTTATGTAGCTTTCCTTGATCGTGAGTTCTTCTT GCACCTCG	GGGTAAACACAGCATTCCGTTATGTAGCATTTCTTGGTTGTG AGCGAGGTGCAAGAAGAACTCACGATCAAGGAAAGCTACAT AACGGAGTGTTGCCGCAGAC
Arti3_seedM8	**F-arti3**<br>TAATACGACTCACTATAGGGTAAACACAGCATTCCGTTATGTAGCATTTCT TGGTTGTGAGCGAGGTGCAAGAAGAACTCACGATCAAG<br>**R-arti3_seedM8**<br>GTCTGCGGCAACACTCCGTTATGTAGCAGTCCTTGATCGTGAGTTCTTCT TGCACCTCG	GGGTAAACACAGCATTCCGTTATGTAGCATTTCTTGGTTGTG AGCGAGGTGCAAGAAGAACTCACGATCAAGGACTGCTACAT AACGGAGTGTTGCCGCAGAC
Arti3_seedM456	**F-arti3_seedW6**<br>TAATACGACTCACTATAGGGTAAACACAGCATTCCGTTATGTAGGATTTCT TGGTTGTGAGCGAGGTGCAAGAAGAACTCACGATCAAG<br>**R-arti3_seedM45**<br>GTCTGCGGCAACACTCCGTTATGTTTCATTCCTTGATCGTGAGTTCTTCTT GCACCTCG	GGGTAAACACAGCATTCCGTTATGTAGGATTTCTTGGTTGTG AGCGAGGTGCAAGAAGAACTCACGATCAAG GAATGAAACATAACGGAGTGTTGCCGCAGAC
Arti3_seedW456	**F-arti3_seedW46**<br>TAATACGACTCACTATAGGGTAAACACAGCATTCCGTTATGTGGGATTTCT TGGTTGTGAGCGAGGTGCAAGAAGAACTCACGATCAAG<br>**R-arti3_seedW56**<br>GTCTGCGGCAACACTCCGTTATGTAAAATTCCTTGATCGTGAGTTCTTCTT GCACCTCG	GGGTAAACACAGCATTCCGTTATGTGGGATTTCTTGGTTGT GAGCGAGGTGCAAGAAGAACTCACGATCAAGGAATTTTACA TAACGGAGTGTTGCCGCAGAC
Arti3_seedW6UG	**F-arti3_seedW6UG**<br>TAATACGACTCACTATAGGGTAAACACAGCATTCCGTTATGTAGTATTTCT TGGTTGTGAGCGAGGTGCAAGAAGAACTCACGATCAAG<br>**R-arti3**<br>GTCTGCGGCAACACTCCGTTATGTAGCATTCCTTGATCGTGAGTTCTTCTT GCACCTCG	GGGTAAACACAGCATTCCGTTATGTAGTATTTCTTGGTTGTG AGCGAGGTGCAAGAAGAACTCACGATCAAGGAATGCTACAT AACGGAGTGTTGCCGCAGAC
Arti3_seedW7UG	**F-arti3_seedW7UG**<br>TAATACGACTCACTATAGGGTAAACACAGCATTCCGTTATGTAGCTTTTCT TGGTTGTGAGCGAGGTGCAAGAAGAACTCACGATCAAG<br>**R-arti3_seedW7UG**<br>GTCTGCGGCAACACTCCGTTATGTAGCCTTCCTTGATCGTGAGTTCTTCTT GCACCTCG	GGGTAAACACAGCATTCCGTTATGTAGCTTTTCTTGGTTGTG AGCGAGGTGCAAGAAGAACTCACGATCAAGGAAGGCTACAT AACGGAGTGTTGCCGCAGAC
Arti3_seedW67UG	**F-arti3_seedW67UG**<br>TAATACGACTCACTATAGGGTAAACACAGCATTCCGTTATGTAGTTTTTCT TGGTTGTGAGCGAGGTGCAAGAAGAACTCACGATCAAG<br>**R-arti3_seedW67UG**<br>GTCTGCGGCAACACTCCGTTATGTAGCCTTCCTTGATCGTGAGTTCTTCTT GCACCTCG	GGGTAAACACAGCATTCCGTTATGTAGTTTTTCTTGGTTGTG AGCGAGGTGCAAGAAGAACTCACGATCAAGGAAGGCTACAT AACGGAGTGTTGCCGCAGAC
Pri-mir-16-1 upper stem 8 bp	**F-mir16-up8**<br>TAATACGACTCACTATAGGGTGCTGAAGTAAGGTTGACCATACGCAAT<br>**R-mir16-up8**<br>GAGTGCTAAGGCACTGCTGACATTGCGTATGGTCAACCTTACTTC	GGGGTGCTGCTGAAGTAAGGTTGACCATACGCAATGTCAGC AGTGCCTTAGCAGCACCTC
Pri-mir-16-1 upper stem 10 bp	**F-mir16-up10**<br>TAATACGACTCACTATAGGGGCTGCTGAAGTAAGGTTGACCATACGCAAT<br>**R-mir16-up10**<br>GAGGCTGCTAAGGCACTGCTGACATTGCGTATGGTCAACCTTACTTC	GGGGCTGCTGAAGTAAGGTTGACCATACGCAATGTCAGCAG TGCCTTAGCAGCCTC

Pri-mir-16-1 upper stem 12 bp	**F-mir16-up12**<br>TAATACGACTCACTATAGGGGTGCTGCTGAAGTAAGGTTGACCATACGCAAT<br>**R-mir16-up12**<br>GAGGTGCTGCTAAGGCACTGCTGACATTGCGTATGGTCAACCTTACTTC	GGGTGCTGAAGTAAGGTTGACCATACGCAATGTCAGCAGTGCCTTAGCACTC
Pri-mir-16-1 upper stem 14 bp	**F-mir16-up14**<br>TAATACGACTCACTATAGGGACGTGCTGCTGAAGTAAGGTTGACCATACGCAAT<br>**R-mir16-up14**<br>GAGACGTGCTGCTAAGGCACTGCTGACATTGCGTATGGTCAACCTTACTTC	GGGACGTGCTGCTGAAGTAAGGTTGACCATACGCAATGTCAGCAGTGCCTTAGCAGCACGTCTC
Pri-mir-16-1 upper stem 24 bp	**F-mir16-up24**<br>TAATACGACTCACTATAGGGTCCAGTATTAACGTGCTGCTGAAGTAAGGTTGACCATACGCAAT<br>**R-mir16-up24**<br>GAGTCCAATATTTACGTGCTGCTAAGGCACTGCTGACATTGCGTATGGTCAACCTTACTTC	GGGTCCAGTATTAACGTGCTGCTGAAGTAAGGTTGACCATACGCAATGTCAGCAGTGCCTTAGCAGCACGTAAATATTGGACTC

Supplementary Table 5. The PCR primers and templates for human pri-miRNA and their variants preparation

The underlined sequence is T7 promoter.

Substrates name	PCR templates	PCR primers (5'-3')	DNA sequences encoding for pri-miRNAs (5'-3')
Pri-mir-16-1	pcDNA3-pri-mir-16-1	**F-T7-16-1 (25-pre)**<br>TAATACGACTCACTATAGGGTGATAGCAATGTCAGCAGTGC<br>**R-16-1 (pre-20)**<br>TAGAGTATGGTCAACCTTAC	GGGTGATAGCAATGTCAGCAGTGCCTTAGCAGCACGTAAATATTGGCGTTAAGATTCTAAAATTATCTCCAGTATTAACTGTGCTGCTGAAGTAAGGTTGACCATACTCTA
Pri-mir-16-1	pcDNA3-pri-mir-16-1	**F-T7-16-1 (25-pre)**<br>TAATACGACTCACTATAGGGTGATAGCAATGTCAGCAGTGC<br>**R-16-1_seedM_56**<br>TAGAGTATGGTCAACCTTACTTCAGCTTCACAGTTAATACTGGAGATAA	GGGTGATAGCAATGTCAGCAGTGCCTTAGCAGCACGTAAATATTGGCGTTAAGATTCTAAAATTATCTCCAGTATTAACTGTGAAGCTGAAGTAAGGTTGACCATACTCTA
Pri-mir-16-1_noUG	T7-pri-mir-16-1 dsDNA	**F-T7-16-1_noUG**<br>TAATACGACTCACTATAGGGTGCTAGCAATATCAGCAGTGCCTTAGCAGCA<br>**R-16-1_noUG**<br>TAGAGTATGATCAACCTTACTTCAGCAGCAC	GGGTGCTAGCAATATCAGCAGTGCCTTAGCAGCACGTAAATATTGGCGTTAAGATTCTAAAATTATCTCCAGTATTAACTGTGCTGCTGAAGTAAGGTTGATCATACTCTA
Pri-mir-16-1_noUG (seedM_56)	T7-pri-mir-16-1 (seedM_56) dsDNA	**F-T7-16-1_noUG**<br>TAATACGACTCACTATAGGGTGCTAGCAATATCAGCAGTGCCTTAGCAGCA<br>**R-16-1_noUG (seedM_56)**<br>TAGAGTATGATCAACCTTACTTCAGCTTCAC	GGGTGCTAGCAATATCAGCAGTGCCTTAGCAGCACGTAAATATTGGCGTTAAGATTCTAAAATTATCTCCAGTATTAACTGTGAAGCTGAAGTAAGGTTGATCATACTCTA
Pri-mir-16-1_GHG	T7-pri-mir-16-1 dsDNA	**F-T7-16-1_GHG**<br>TAATACGACTCACTATAGGGTGATAGCAATGTCAGCCTCGCCTTAGCAGCACGTAAATA<br>**R-16-1_GHG**<br>TAGAGTATGGTCAACCGCACTTCAGCAGCACAGTTAAT	GGGTGATAGCAATGTCAGCCTCGCCTTAGCAGCACGTAAATATTGGCGTTAAGATTCTAAAATTATCTCCAGTATTAACTGTGCTGCTGAAGTGCGGTTGACCATACTCTA
Pri-mir-16-1_GHG (seedM_56)	T7-pri-mir-16-1 (seedM_56) dsDNA	**F-T7-16-1_GHG**<br>TAATACGACTCACTATAGGGTGATAGCAATGTCAGCCTCGCCTTAGCAGCACGTAAATA<br>**R-16-1_GHG (seedM_56)**<br>TAGAGTATGGTCAACCGCACTTCAGCTTCACAGTTAATACTGGAGATA	GGGTGATAGCAATGTCAGCCTCGCCTTAGCAGCACGTAAATATTGGCGTTAAGATTCTAAAATTATCTCCAGTATTAACTGTGAAGCTGAAGTGCGGTTGACCATACTCTA
Pri-mir-16-1 _UGU	N/A	**F-T7-16-1_UGU**<br>TAATACGACTCACTATAGGGTGATAGCAATGTCAGCAGTGCCTTAGCAGCACGTAAATATTGGCTGTAAGATT<br>**R-16-1_UGU**<br>TAGAGTATGGTCAACCTTACTTCAGCAGCACAGTTAATACTGGCTTGAATTTTAGAATCTTACAGCCAATATTTA	GGGTGATAGCAATGTCAGCAGTGCCTTAGCAGCACGTAAATATTGGCTGTAAGATTCTAAAATTCAAGCCAGTATTAACTGTGCTGCTGAAGTAAGGTTGACCATACTCTA
Pri-mir-16-1_UGU (seedM_56)	N/A	**F-T7-16-1_UGU**<br>TAATACGACTCACTATAGGGTGATAGCAATGTCAGCAGTGCCTTAGCAGCACGTAAATATTGGCTGTAAGATT<br>**R-16-1_UGU (seedM_56)**<br>TAGAGTATGGTCAACCTTACTTCAGCTTCACAGTTAATACTGGCTTGAATTTTAGAATCTTACAGCCAATATTTA	GGGTGATAGCAATGTCAGCAGTGCCTTAGCAGCACGTAAATATTGGCTGTAAGATTCTAAAATTCAAGCCAGTATTAACTGTGAAGCTGAAGTAAGGTTGACCATACTCTA
Pri-mir-128-1 (midM_91011, WT)	TUT4 KO Hela cell genomic DNA	**F-T7-128-1**<br>TAATACGACTCACTATAGGGCCTTGTTCCTGAGCTGTTGGATT<br>**R-128-1** AAAAAGAAGCCAGGAAGCAGCTGAA	GGGCCTTGTTCCTGAGCTGTTGGATTCGGGGCCGTAGCACTGTCTGAGAGGTTTACATTTCTCACAGTGAACCGGTCTCTTTTTCAGCTGCTTCCTGGCTTCTTTTT
Pri-mir-128-1 (midM_none)	TUT4 KO Hela cell genomic DNA	**F-T7-128-1**<br>TAATACGACTCACTATAGGGCCTTGTTCCTGAGCTGTTGGATT<br>**R-128-1 (midM_none)**<br>AAAAAGAAGCCAGGAAGCAGCTGAAAAAGAGACCGTAGCACTGTGAGAAATGTA	GGGCCTTGTTCCTGAGCTGTTGGATTCGGGGCCGTAGCACTGTCTGAGAGGTTTACATTTCTCACAGTGCTACGGTCTCTTTTTCAGCTGCTTCCTGGCTTCTTTTT
Pri-mir-128-1_noUG	T7-pri-mir-128-1 dsDNA	**F-T7-128-1_noUG**<br>TAATACGACTCACTATAGGGCCTTGTTCCTAAGCTGTTGGATTCGGGGCC<br>**R-128-1** AAAAAGAAGCCAGGAAGCAGCTGAA	GGGCCTTGTTCCTAAGCTGTTGGATTCGGGGCCGTAGCACTGTCTGAGAGGTTTACATTTCTCACAGTGAACCGGTCTCTTTTTCAGCTGCTTCCTGGCTTCTTTTT

Pri-mir-128-1_noUG (midM_none)	T7-pri-mir-128-1_ midM_none dsDNA	**F-T7-128-1_noUG**<br>TAATACGACTCACTATAGGGCCTTGTTCCTAAGCTGTTGGATTCGGGGCC<br>**R-128-1 (midM_none)**<br>AAAAAGAAGCCAGGAAGCAGCTGAAAAAGAGACCGTAGCACTGTGAGAAATGTA	GGGCCTTGTTCCTAAGCTGTTGGATTCGGGGCCGTAGCACTGTCTGAGAGGTTTACATTTCTCACAGTGCTACGGTCTCTTTTTCAGCTGCTTCCTGGCTTCTTTTT
Pri-mir-128-1_GHG	T7-pri-mir-128-1 dsDNA	**F-T7-128-1_GHG**<br>TAATACGACTCACTATAGGGCCTTGTTCCTGAGCTGCTCGATTCGGGGCCGTAGCACTG<br>**R-128-1_GHG**<br>AAAAAGAAGCCAGGAAGCAGCGCAAAAAGAGACCGGTTCACTG	GGGCCTTGTTCCTGAGCTGCTCGATTCGGGGCCGTAGCACTGTCTGAGAGGTTTACATTTCTCACAGTGAACCGGTCTCTTTTTGCGCTGCTTCCTGGCTTCTTTTT
Pri-mir-128-1_GHG (midM_none)	T7-pri-mir-128-1_ midM_none dsDNA	**F-T7-128-1_GHG**<br>TAATACGACTCACTATAGGGCCTTGTTCCTGAGCTGCTCGATTCGGGGCCGTAGCACTG<br>**R-128-1_GHG (midM_none)**<br>AAAAAGAAGCCAGGAAGCAGCGCAAAAAGAGACCGTAGCACTGTGAGAAATGTAAACC	GGGCCTTGTTCCTGAGCTGCTCGATTCGGGGCCGTAGCACTGTCTGAGAGGTTTACATTTCTCACAGTGCTACGGTCTCTTTTTGCGCTGCTTCCTGGCTTCTTTTT
Pri-mir-128-1_UGU	N/A	**F-T7-128-1_UGU**<br>TAATACGACTCACTATAGGGCCTTGTTCCTGAGCTGTTGGATTCGGGGCCGTAGCACTGTCTGAGATGTTT<br>**R-128-1_UGU**<br>AAAAAGAAGCCAGGAAGCAGCTGAAAAAGAGACCGGTTCACTGTGAGATTGGTAAACATCTCAGACAGTGCTAC	GGGCCTTGTTCCTGAGCTGTTGGATTCGGGGCCGTAGCACTGTCTGAGATGTTTACCAATCTCACAGTGAACCGGTCTCTTTTTCAGCTGCTTCCTGGCTTCTTTTT
Pri-mir-128-1_UGU (midM_none)	N/A	**F-T7-128-1_UGU**<br>TAATACGACTCACTATAGGGCCTTGTTCCTGAGCTGTTGGATTCGGGGCCGTAGCACTGTCTGAGATGTTT<br>**R-128-1_UGU (midM_none)**<br>AAAAAGAAGCCAGGAAGCAGCTGAAAAAGAGACCGTAGCACTGTGAGATTGGTAAACATCTCAGACAGTGCTAC	GGGCCTTGTTCCTGAGCTGTTGGATTCGGGGCCGTAGCACTGTCTGAGATGTTTACCAATCTCACAGTGCTACGGTCTCTTTTTCAGCTGCTTCCTGGCTTCTTTTT
Pri-mir-30a (seedW_none, WT)	pcDNA3-pri-mir-30a	**F-T7-30a**<br>TAATACGACTCACTATAGGGTATTGCTGTTGACAGTGAGCGACTG<br>**R-30a** TGAAGTCCGAGGCAGTAGGCAGCTGC	GGGTATTGCTGTTGACAGTGAGCGACTGTAAACATCCTCGACTGGAAGCTGTGAAGCCACAGATGGGCTTTCAGTCGGATGTTTGCAGCTGCCTACTGCCTCGGACTTCA
Pri-mir-30a (seedW56)	pcDNA3-pri-mir-30a	**F-T7-30a (seedW56)**<br>TAATACGACTCACTATAGGGTATTGCTGTTGACAGTGAGCGACTGTAGGCATCC<br>**R-mir30a** TGAAGTCCGAGGCAGTAGGCAGCTGC	GGGTATTGCTGTTGACAGTGAGCGACTGTAGGCATCCTCGACTGGAAGCTGTGAAGCCACAGATGGGCTTTCAGTCGGATGTTTGCAGCTGCCTACTGCCTCGGACTTCA
Pri-mir-142 (seedW_none, WT)	pcDNA3-pri-mir-142	**F-T7-142**<br>TAATACGACTCACTATAGGGCGGACAGACAGACAGTGCAGTC<br>**R-142** CCGAAGCCCACAGTACACTCATCC	GGGCGGACAGACAGACAGTGCAGTCACCCATAAAGTAGAAAGCACTACTAACAGCACTGGAGGGTGTAGTGTTTCCTACTTTATGGATGAGTGTACTGTGGGCTTCGG
Pri-mir-142 (seedW67)	pcDNA3-pri-mir-142	**T7-F-mir142_GGUU**<br>TAATACGACTCACTATAGGCGGACAGACAGACAGTGCAGTCACCCATGGAGTAG<br>**R-T7-mir142** CCGAAGCCCACAGTACACTCATCC	GGGCGGACAGACAGACAGTGCAGTCACCCATGGAGTAGAAAGCACTACTAACAGCACTGGAGGGTGTAGTGTTTCCTACTTTATGGATGAGTGTACTGTGGGCTTCGG
Pri-let-7d (seedW_none, WT)	pcDNA3-pri-let-7d	**F-T7-let-7d**<br>TAATACGACTCACTATAGGGAAAAAAAAAATGGGTTCCTAGGAAG<br>**R-let-7d** AGGTTATCGGTGAATAATAAGGCCC	GGGAAAAAAAAAATGGGTTCCTAGGAAGAGGTAGTAGGTTGCATAGTTTTAGGGCAGGGATTTTGCCCACAAGGAGGTAACTATACGACCTGCTGCCTTTCTTAGGGCCTTATTATTCACCGATAACCT
Pri-let-7d (seedW56, AU)	pcDNA3-pri-let-7d	**F-T7-let7d (seedW_none, AU)**<br>AU)TAATACGACTCACTATAGGGAAAAAAAAAATGGGTTCCTAGGAAGAGAAAGTAG<br>**R-let-7d (seedW_none, AU)**<br>AGGTTATCGGTGAATAATAAGGCCCTAAGAAAGAAAGCAG	GGGAAAAAAAAAATGGGTTCCTAGGAAGAGAAAGTAGGTTGCATAGTTTTAGGGCAGGGATTTTGCCCACAAGGAGGTAACTATACGACCTGCTTTCTTTCTTAGGGCCTTATTATTCACCGATAACCT
Pri-let-7d (seedW56, GU)	pcDNA3-pri-let-7d	**F-T7-let-7d (seedW_56, GU)**<br>TAATACGACTCACTATAGGGAAAAAAAAAATGGGTTCCTAGGAAGAGGGAGTAG<br>**R-let-7d (seedW_56, GU)**<br>AGGTTATCGGTGAATAATAAGGCCCTAAGAAAGAAAGCAG	GGGAAAAAAAAAATGGGTTCCTAGGAAGAGGGAGTAGGTTGCATAGTTTTAGGGCAGGGATTTTGCCCACAAGGAGGTAACTATACGACCTGCTTTCTTTCTTAGGGCCTTATTATTCACCGATAACCT
Pri-mir-885 (midM_none)	TUT4 KO Hela cell genomic DNA	**F-T7-885**<br>TAATACGACTCACTATAGGGCTACTCGGCCCGCACTCTCTCCATTAC<br>**R-885** CGGGACTTGAACCCGTGCTCTATCC	GGGCTACTCGGCCCGCACTCTCTCCATTACACTACCCTGCCTCTTCTCCATGAGAGGCAGCGGGGTGTAGTGGATAGAGCACGGGTTCAAGTCCCG
Pri-mir-885 (midM_1011, SNP)	TUT4 KO Hela cell genomic DNA	**F-T7-885**<br>TAATACGACTCACTATAGGGCTACTCGGCCCGCACTCTCTCCATTAC<br>**R-885 (midM_1011, SNP)**<br>CGGGACTTGAACCCGTGCTCTATCCACTACAGCCCGCTGCCTCTCATGGAG	GGGCTACTCGGCCCGCACTCTCTCCATTACACTACCCTGCCTCTTCTCCATGAGAGGCAGCGGGCTGTAGTGGATAGAGCACGGGTTCAAGTCCCG
Pri-mir-342 (midM_89, WT)	TUT4 KO Hela cell genomic DNA	**F-T7-342**<br>TAATACGACTCACTATAGGGTGAAACTGGGCTCAAGGTGAGG<br>**R-342** GTGATAAGTAGGCCAAGGTGACGG	GGGTGAAACTGGGCTCAAGGTGAGGGGTGCTATCTGTGATTGAGGGACATGGTTAATGGAATTGTCTCACACAGAAATCGCACCCGTCACCTTGGCCTACTTATCAC
Pri-mir-342 (midM_none)	TUT4 KO Hela cell genomic DNA	**F-T7-342**<br>TAATACGACTCACTATAGGGTGAAACTGGGCTCAAGGTGAGG<br>**R-342 (midM_none)**<br>GTGATAAGTAGGCCAAGGTGACGGGTGCTATTTCTGTGTGAGACAATTCC	GGGTGAAACTGGGCTCAAGGTGAGGGGTGCTATCTGTGATTGAGGGACATGGTTAATGGAATTGTCTCACACAGAAATAGCACCCGTCACCTTGGCCTACTTATCAC
Pri-mir-342 (noJC)	TUT4 KO Hela cell genomic DNA	**F-T7-342**<br>TAATACGACTCACTATAGGGTGAAACTGGGCTCAAGGTGAGG<br>**R-342 (noJC)**<br>GTGATAAATAGGCCAAGGTGACGGGTGCGATTTC	GGGTGAAACTGGGCTCAAGGTGAGGGGTGCTATCTGTGATTGAGGGACATGGTTAATGGAATTGTCTCACACAGAAATCGCACCCGTCACCTTGGCCTATTTATCAC
Pri-mir-342 (midM_910)	pcDNA3-pri-mir-342 (midM_910)	**F-T7-342**<br>TAATACGACTCACTATAGGGTGAAACTGGGCTCAAGGTGAGG<br>**R-342 (midM_910)** GTGATAAGTAGGCCAAGGTGACGG	GGGTGAAACTGGGCTCAAGGTGAGGGGTGCTTACTGTGATTGAGGGACATGGTTAATGGAATTGTCTCACACAGAATCAGCACCCGTCACCTTGGCCTACTTATCAC

Pri-mir-342_UG (midM_89)	T7-pri-mir-342 dsDNA	**F-T7-342_UG** TAATACGACTCACTATAGGGTGAAATGGGGCTCAAGGTGAGGGGTGC **R-342_UG** GTGATAAGAAGGCCAAGGTGACGGGTGCG	GGGTGAAATGGGGCTCAAGGTGAGGGGTGCTATCTGTGATTGAGGGACATGGTTAATGGAATTGTCTCACACAGAAATCGCACCCGTCACCTTGGCCTTCTTATCAC
Pri-mir-342_UG (midM_none)	T7-pri-mir-342 (midM_none) dsDNA	**F-T7-342_UG** TAATACGACTCACTATAGGGTGAAATGGGGCTCAAGGTGAGGGGTGC **R-342_UG (midM_none)** GTGATAAGAAGGCCAAGGTGACGGGTGCTATTTCTGTGTGAGACAATTC	GGGTGAAATGGGGCTCAAGGTGAGGGGTGCTATCTGTGATTGAGGGACATGGTTAATGGAATTGTCTCACACAGAAATAGCACCCGTCACCTTGGCCTTCTTATCAC
Pri-mir-342_GHG	T7-pri-mir-342 dsDNA	**F-T7-342_GHG** TAATACGACTCACTATAGGGTGAAACTGGGCTCCTCGTGAGGGGTGCTATCTGTGA **R-342_GHG** GTGATAAGTAGGCCCGCGTGACGGGTGCGATTTCTGT	GGGTGAAACTGGGCTCCTCGTGAGGGGTGCTATCTGTGATTGAGGGACATGGTTAATGGAATTGTCTCACACAGAAATCGCACCCGTCACGCGGGCCTACTTATCAC
Pri-mir-342_GHG (midM_none)	T7-pri-mir-342 (midM_none) dsDNA	**F-T7-342_GHG** TAATACGACTCACTATAGGGTGAAACTGGGCTCCTCGTGAGGGGTGCTATCTGTGA **R-342_GHG (midM_none)** GTGATAAGTAGGCCCGCGTGACGGGTGCTATTTCTGTGTGAGACAATTC	GGGTGAAACTGGGCTCCTCGTGAGGGGTGCTATCTGTGATTGAGGGACATGGTTAATGGAATTGTCTCACACAGAAATAGCACCCGTCACGCGGGCCTACTTATCAC
Pri-mir-342_UGUG	N/A	**F-T7-342_UGU** TAATACGACTCACTATAGGGTGAAACTGGGCTCAAGGTGAGGGGTGCTATCTGTGATTGAGTGTGCATGGT **R-342_UGU** GTGATAAGTAGGCCAAGGTGACGGGTGCGATTTCTGTGTGAGCCAATTCCATTAACCATGCACACTCAATCACA	GGGTGAAACTGGGCTCAAGGTGAGGGGTGCTATCTGTGATTGAGTGTGCATGGTTAATGGAATTGGCTCACACAGAAATCGCACCCGTCACCTTGGCCTACTTATCAC
Pri-mir-342_UGUG (midM_none)	N/A	**F-T7-342_UGU** TAATACGACTCACTATAGGGTGAAACTGGGCTCAAGGTGAGGGGTGCTATCTGTGATTGAGTGTGCATGGT **R-342_UGUG (midM_none)** GTGATAAGTAGGCCAAGGTGACGGGTGCTATTTCTGTGTGAGCCAATTCCATTAACCATGCACACTCAATCACA	GGGTGAAACTGGGCTCAAGGTGAGGGGTGCTATCTGTGATTGAGTGTGCATGGTTAATGGAATTGGCTCACACAGAAATAGCACCCGTCACCTTGGCCTACTTATCAC
Pri-mir-30d (seedM_none, WT)	TUT4 KO Hela cell genomic DNA	**F-T7-30d** TAATACGACTCACTATAGGGTCTTAAATTTCTTGTTCAGAAAG **R-30d** GAGGATGTCTGTGAATAGCCGGTAG	GGGCTCTTAAATTTCTTGTTCAGAAAGTCTGTTGTTGTAAACATCCCCGACTGGAAGCTGTAAGACACAGCTAAGCTTTCAGTCAGATGTTTGCTGCTACCGGCTATTCACAGACATCCTC
Pri-mir-30d (seedM_6, SNP)	TUT4 KO Hela cell genomic DNA	**F-T7-30d** TAATACGACTCACTATAGGGTCTTAAATTTCTTGTTCAGAAAG **R-30d (seedM6, SNP)** GAGGATGTCTGTGAATAGCCGGTAGCAGCAAGCATCTGACTGAAAGCTTAG	GGGCTCTTAAATTTCTTGTTCAGAAAGTCTGTTGTTGTAAACATCCCCGACTGGAAGCTGTAAGACACAGCTAAGCTTTCAGTCAGATGCTTGCTGCTACCGGCTATTCACAGACATCCTC
Pri-mir-30d (seedM_56)	T7-pri-mir-30d dsDNA	**F-30d-T7** TAATACGACTCACTATAGGGTCTTAAATTTCTTGTTCAGAAAG **R-30d (seedM_56)** GAGGATGTCTGTGAATAGCCGGTAGCAGCAGGCATCTGACTGAAAGCTTAGC	GGGTCTTAAATTTCTTGTTCAGAAAGTCTGTTGTTGTAAACATCCCCGACTGGAAGCTGTAAGACACAGCTAAGCTTTCAGTCAGATGCCTGCTGCTACCGGCTATTCACAGACATCCTC
Pri-mir-30d_UG	T7-pri-mir-30d dsDNA	**F-T7-30d_UG** TAATACGACTCACTATAGGGTCTTAAATTTCTTGTCCTGAAAGTCTGTTGTTGTAAACA **R-30d_UG** GAGGATGTCTGAGAATAGCCGGTAGCAGCAAAC **R-30d_56M_UG** GAGGATGTCTGAGAATAGCCGGTAGCAGCAGGC	GGGTCTTAAATTTCTTGTCCTGAAAGTCTGTTGTTGTAAACATCCCCGACTGGAAGCTGTAAGACACAGCTAAGCTTTCAGTCAGATGTTTGCTGCTACCGGCTATTCTCAGACATCCTC
Pri-mir-30d_UG (seedM_56)	T7-pri-mir-30d (seedM_56) dsDNA	**F-T7-30d_UG** TAATACGACTCACTATAGGGTCTTAAATTTCTTGTCCTGAAAGTCTGTTGTTGTAAACA **R-30d_UG (seedM_56)** GAGGATGTCTGAGAATAGCCGGTAGCAGCAGGC	GGGTCTTAAATTTCTTGTCCTGAAAGTCTGTTGTTGTAAACATCCCCGACTGGAAGCTGTAAGACACAGCTAAGCTTTCAGTCAGATGCCTGCTGCTACCGGCTATTCTCAGACATCCTC
Pri-mir-30d_noGHG	T7-pri-mir-30d dsDNA	**F-T7-30d_noGHG** TAATACGACTCACTATAGGGTCTTAAATTTCTTGTTCAGAAAGTTTTTTGTTGTAAACATCCCCGAC **R-30d_noGHG** GAGGATGTCTGTGAATAGCTTTTAGCAGCAAACATCTGACTG	GGGTCTTAAATTTCTTGTTCAGAAAGTTTTTTGTTGTAAACATCCCCGACTGGAAGCTGTAAGACACAGCTAAGCTTTCAGTCAGATGTTTGCTGCTAAAAGCTATTCACAGACATCCTC
Pri-mir-30d_noGHG (seedM_56)	T7-pri-mir-30d (seedM_56) dsDNA	**F-T7-30d_noGHG** TAATACGACTCACTATAGGGTCTTAAATTTCTTGTTCAGAAAGTTTTTTGTTGTAAACATCCCCGAC **R-30d_noGHG (seedM_56)** GAGGATGTCTGTGAATAGCTTTTAGCAGCAGGCATCTGACTGAAAGCTTAGC	GGGTCTTAAATTTCTTGTTCAGAAAGTTTTTTGTTGTAAACATCCCCGACTGGAAGCTGTAAGACACAGCTAAGCTTTCAGTCAGATGCCTGCTGCTAAAAGCTATTCACAGACATCCTC
Pri-mir-30d_noUGU	N/A	**F-T7-30d_noUGU** TAATACGACTCACTATAGGGTCTTAAATTTCTTGTTCAGAAAGTCTGTTGTTGTAAACATCCCCGACTGGAAGCTAAAA **R-30d_noUGU** GAGGATGTCTGTGAATAGCCGGTAGCAGCAAACATCTGACTGAAAGCTTAGCTGTGTCTTTTAGCTTCCAGTCGGGGA	GGGTCTTAAATTTCTTGTTCAGAAAGTCTGTTGTTGTAAACATCCCCGACTGGAAGCTAAAAGACACAGCTAAGCTTTCAGTCAGATGTTTGCTGCTACCGGCTATTCACAGACATCCTC
Pri-mir-30d_noUGU (seedM_56)	N/A	**F-T7-30d_noUGU** TAATACGACTCACTATAGGGTCTTAAATTTCTTGTTCAGAAAGTCTGTTGTTGTAAACATCCCCGACTGGAAGCTAAAA **R-30d_noUGU (seedM_56)** GAGGATGTCTGTGAATAGCCGGTAGCAGCAGGCATCTGACTGAAAGCTTAGCTGTGTCTTTTAGCTTCCAGTCGGGGA	GGGTCTTAAATTTCTTGTTCAGAAAGTCTGTTGTTGTAAACATCCCCGACTGGAAGCTAAAAGACACAGCTAAGCTTTCAGTCAGATGCCTGCTGCTACCGGCTATTCACAGACATCCTC

Pri-mir-576 (seedM_none, WT)	TUT4 KO Hela cell genomic DNA	**F-T7-576**<br>TAATACGACTCACTATAGGGTAACTGCACCATTTTACAA<br>TCCAAC<br>**R-576** GATGGTTATAACCAATCGAATGAGGATTCC	GGGTAACTGCACCATTTTACAATCCAACGAGGATTCTA<br>ATTTCTCCACGTCTTTGGTAATAAGGTTTGGCAAAGATG<br>TGGAAAAATTGGAATCCTCATTCGATTGGTTATAACCAT<br>C
Pri-mir-576 (seedM6, SNP)	TUT4 KO Hela cell genomic DNA	**F-T7-576**<br>TAATACGACTCACTATAGGGTAACTGCACCATTTTACAA<br>TCCAAC<br>**R-576 (seedM6, SNP)**<br>GATGGTTATAACCAATCGAATGAGGATTCCCATTTTTCC<br>ACATCTTTGCCAAACC	GGGTAACTGCACCATTTTACAATCCAACGAGGATTCTA<br>ATTTCTCCACGTCTTTGGTAATAAGGTTTGGCAAAGATG<br>TGGAAAAATGGGAATCCTCATTCGATTGGTTATAACCAT<br>C
Pri-mir-576 (midM_91011)	T7-pri-mir-576 dsDNA	**F-T7-576**<br>TAATACGACTCACTATAGGGTAACTGCACCATTTTACAA<br>TCCAAC<br>**R-576 (midM_91011)**<br>GATGGTTATAACCAATCGAATGAGGATTCCAATTGTGC<br>CACATCTTTGCCAAACCTT	GGGTAACTGCACCATTTTACAATCCAACGAGGATTCTA<br>ATTTCTCCACGTCTTTGGTAATAAGGTTTGGCAAAGATG<br>TGGCACAATTGGAATCCTCATTCGATTGGTTATAACCAT<br>C
Pri-mir-576_UG	T7-pri-mir-576 dsDNA	**F-T7-576_UG**<br>TAATACGACTCACTATAGGGTAACTGCACCATTTTTGAA<br>TCCAACGAGGATTCTAAT<br>**R-576_UG**<br>GATGGTTATAAGGAATCGAATGAGGATTCCAAT	GGGTAACTGCACCATTTTTGAATCCAACGAGGATTCTA<br>ATTTCTCCACGTCTTTGGTAATAAGGTTTGGCAAAGATG<br>TGGAAAAATTGGAATCCTCATTCGATTCCTTATAACCAT<br>C
Pri-mir-576_UG (midM_91011)	Pri-mir-576 (midM_91011) dsDNA	**F-T7-576_UG**<br>TAATACGACTCACTATAGGGTAACTGCACCATTTTTGAA<br>TCCAACGAGGATTCTAAT<br>**R-576_UG (midM_91011)**<br>GATGGTTATAAGGAATCGAATGAGGATTCCAATTGTGC<br>CACATCTTTGCCAAACCTT	GGGTAACTGCACCATTTTTGAATCCAACGAGGATTCTA<br>ATTTCTCCACGTCTTTGGTAATAAGGTTTGGCAAAGATG<br>TGGCACAATTGGAATCCTCATTCGATTCCTTATAACCAT<br>C
Pri-mir-576_GHG	T7-pri-mir-576 dsDNA	**F-T7-576_GHG**<br>TAATACGACTCACTATAGGGTAACTGCACCATTTTACAA<br>TCCCTCGAGGATTCTAATTTCTCCAC<br>**R-576_GHG**<br>GATGGTTATAACCAATCGCGCGAGGATTCCAATTTTTC<br>CAC	GGGTAACTGCACCATTTTACAATCCCTCGAGGATTCTA<br>ATTTCTCCACGTCTTTGGTAATAAGGTTTGGCAAAGATG<br>TGGAAAAATTGGAATCCTCGCGCGATTGGTTATAACCA<br>TC
Pri-mir-576_GHG (midM_91011)	T7-pri-mir-576 (midM_91011) dsDNA	**F-T7-576_GHG**<br>TAATACGACTCACTATAGGGTAACTGCACCATTTTACAA<br>TCCCTCGAGGATTCTAATTTCTCCAC<br>**R-576_GHG (midM_91011)**<br>GATGGTTATAACCAATCGCGCGAGGATTCCAATTGTGC<br>CACATCTTTGCCAAACCTT	GGGTAACTGCACCATTTTACAATCCAACGAGGATTCTA<br>ATTTCTCCACGTCTTTGTTAATAAGGTTTGTAAAAGATG<br>TGGAAAAATTGGAATCCTCATTCGATTGGTTATAACCAT<br>C
Pri-mir-576_UGU	T7-pri-mir-576 dsDNA	**F-T7-576_UGU**<br>TAATACGACTCACTATAGGGTAACTGCACCATTTTACAA<br>TCCAACGAGGATTCTAATTTCTCCACGTCTTTGT<br>**R-576_UGU**<br>GATGGTTATAACCAATCGAATGAGGATTCCAATTTTTCC<br>ACATCTTTTACAAACCTTATTAACAAAGA	GGGTAACTGCACCATTTTACAATCCAACGAGGATTCTA<br>ATTTCTCCACGTCTTTGTTAATAAGGTTTGTAAAAGATG<br>TGGAAAAATTGGAATCCTCATTCGATTGGTTATAACCAT<br>C
Pri-mir-576_UGU (midM_91011)	T7-pri-mir-576 (midM_91011) dsDNA	**F-T7-576_UGU**<br>TAATACGACTCACTATAGGGTAACTGCACCATTTTACAA<br>TCCAACGAGGATTCTAATTTCTCCACGTCTTTGT<br>**R-576_UGU (midM_91011)**<br>GATGGTTATAACCAATCGAATGAGGATTCCAATTGTGC<br>CACATCTTTTACAAACCTTATTAACAAAGA	GGGTAACTGCACCATTTTACAATCCAACGAGGATTCTA<br>ATTTCTCCACGTCTTTGTTAATAAGGTTTGTAAAAGATG<br>TGGCACAATTGGAATCCTCATTCGATTGGTTATAACCAT<br>C
Pri-mir-200b (midW_89, WT)	TUT4 KO Hela cell genomic DNA	**F-T7-200b**<br>TAATACGACTCACTATAGGGCCAGCTCGGGCAGCCGT<br>GGC CATC<br>**R-200b**<br>CTGCGTGCAGGGCTCCGCCGTCATC	GGGCCAGCTCGGGCAGCCGTGGCCATCTTACTGGGCA<br>GCATTGGATGGAGTCAGGTCTCTAATACTGCCTGGTAA<br>TGATGACGGCGGAGCCCTGCACGCAG
Pri-mir-200b (midW_none)	T7-pri-mir-200b dsDNA	**F-T7-200b**<br>TAATACGACTCACTATAGGGCCAGCTCGGGCAGCCGT<br>GGC CATC<br>**R-200b (midW_none)**<br>CTGCGTGCAGGGCTCCGCCGTCATCATTACTGGGCAG<br>TATTAGAGACCTGAC	GGGCCAGCTCGGGCAGCCGTGGCCATCTTACTGGGCA<br>GCATTGGATGGAGTCAGGTCTCTAATACTGCCCAGTAA<br>TGATGACGGCGGAGCCCTGCACGCAG
Pri-mir-1277 (SeedM_none, WT)	TUT4 KO Hela cell genomic DNA	**F-T7-1277**<br>TAATACGACTCACTATAGGGTCCATTTCTAACCTCCCAA<br>**R-1277**<br>AAGATGAAAAAAATTAAACCCACCA	GGGTCCATTTCTAACCTCCCAAATATATATATATATGTA<br>CGTATGTGTATATAAATGTATACGTAGATATATATGTATT<br>TTTGGTGGGTTTAATTTTTTTCATCTT
Pri-mir-1277 (SeedM_567)	T7-pri-mir-1277 dsDNA	**F-T7-1277**<br>TAATACGACTCACTATAGGGTCCATTTCTAACCTCCCAA<br>**R-1277 (SeedM_567)**<br>AAGATGAAAAAAATTAAACCCACCAAAAATAGGGATATA<br>TCTACGTATACATTT	GGGTCCATTTCTAACCTCCCAAATATATATATATATGTA<br>CGTATGTGTATATAAATGTATACGTAGATATATCCCTAT<br>TTTTGGTGGGTTTAATTTTTTTCATCTT

Supplementary Table 6. The pri-miRNA expression plasmids

Backbone vector	Inserted sequence name	PCR primers (5'-3')	PCR templates
pcDNA3	pri-mir-342 (midM_89, WT)	**F-BamHI-342**<br>CGCGGATCCGGGTGAAACTGGGCTCAAGGTG<br>**R-EcoRI-342**<br>TCTGCAGAATTCGTGATAAGTAGGCCAAGGTGACG	T7-pri-mir-342 (midM_89, WT)
pcDNA3	pri-mir-342 (midM_none)	**F-BamHI-342**<br>CGCGGATCCGGGTGAAACTGGGCTCAAGGTG<br>**R-EcoRI-342**<br>TCTGCAGAATTCGTGATAAGTAGGCCAAGGTGACG	T7-pri-mir-342 (midM_none)
pcDNA3	pri-mir-342 (midM_910)	**F-BamHI-342**<br>CGCGGATCCGGGTGAAACTGGGCTCAAGGTG<br>**R-EcoRI-342**	T7-pri-mir-342 (midM_910)

		TCTGCAGAATTCGTGATAAGTAGGCCAAGGTGACG	
pcDNA3	pri-mir-30d (seedM_none, WT)	**F-BamHI-30d**<br>CGCGGATCCGGGTCTTAAATTTCTTGTTCAGAAAG<br>**R-EcoRI-30d**<br>TCTGCAGAATTCGAGGATGTCTGTGAATAGCCGGTAG	T7-pri-mir-30d (seedM_none, WT) dsDNA
pcDNA3	pri-mir-30d (seedM6, SNP)	**F-BamHI-30d**<br>CGCGGATCCGGGTCTTAAATTTCTTGTTCAGAAAG<br>**R-EcoRI-30d**<br>TCTGCAGAATTCGAGGATGTCTGTGAATAGCCGGTAG	T7-pri-mir-30d (seedM6, SNP) dsDNA
pcDNA3	pri-mir-576 (seedM_none, WT)	**F-BamHI-576**<br>CGGGATCCGGG GTAACTGCACCATTTTACAATCC<br>**R-EcoRI-576**<br>TCTGCAGAATTCGATGGTTATAACCAATCGAATG	T7-pri-mir-576 (seedM_none, WT) dsDNA
pcDNA3	pri-mir-576 (seedM6, SNP)	**F-BamHI-576**<br>CGGGATCCGGG GTAACTGCACCATTTTACAATCC<br>**R-EcoRI-576**<br>TCTGCAGAATTCGATGGTTATAACCAATCGAATG	T7-pri-mir-576 (seedM6, SNP) dsDNA
pcDNA3	pri-mir-128-1 (midM_91011, WT)	**F-BamHI-128-1**<br>CGCGGATCCGGGCCTTGTTCCTGAGCTGTTGG<br>**R-EcoRI-128-1**<br>TCTGCAGAATT AAAAAGAAGCCAGGAAGCAGCTG	T7-pri-mir-128-1 (midM_91011, WT) dsDNA
pcDNA3	pri-mir-128-1 (midM_none)	**F-BamHI-128-1**<br>CGCGGATCCGGGCCTTGTTCCTGAGCTGTTGG<br>**R-EcoRI-128-1**<br>TCTGCAGAATT AAAAAGAAGCCAGGAAGCAGCTG	T7-pri-mir-128-1 (midM_none) dsDNA
pcDNA3	pri-mir-885 (midM_none)	**F-BamHI-885**<br>CGCGGATCCGGGCTACTCGGCCCGCACTCTC<br>**R-EcoRI-885**<br>TCTGCAGAATTCCGGGACTTGAACCCGTGCTCTATCC	T7-pri-mir-885 (midM_none) dsDNA
pcDNA3	pri-mir-885 (midM_1011, SNP)	**F-BamHI-885**<br>CGCGGATCCGGGCTACTCGGCCCGCACTCTC<br>**R-EcoRI-885**<br>TCTGCAGAATTCCGGGACTTGAACCCGTGCTCTATCC	T7-pri-mir-885 (midM_1011, SNP) dsDNA
pcDNA3	pri-mir-30a (seedW56)	**IF-30a (seedW56)**<br>AAGCTGTGAAGCCACAGATGGGCTTTCAGTCGGATGTTTGCAGCTG CCTACTGCCTCGG<br>**IR-30a (seedW56)**<br>GTGGCTTCACAGCTTCCAGTCGAGGATGCCTACAGTCGCTCACTGT CAAC	pcDNA3-pri-mir-30a
pcDNA3	pri-mir-142 (seedW67)	**IF-142 (seedW67)**<br>AACAGCACTGGAGGGTGTAGTGTTTCCTACTTTATGGA TGAGTGTACTG<br>**IR-142 (seedW67)** CCCTCCAGTGCTGTTAGTAGTGCTTTCTACT CC ATGGGTGACTGCACTG	pcDNA3-pri-mir-142
pcDNA3-pri-mir-30a [1]	pri-mir-30a	N/A	N/A
pcDNA3-pri-mir-16-1 [1]	Pri-mir-16-1	N/A	N/A
pcDNA3-pri-mir-142 [2]	pri-mir-142	N/A	N/A
pcDNA3	pri-mir-200b	**F-BamHI-200b**<br>CGCGGATCCCGGACCCAGCTCGGGCAGCCGTGGC<br>**R-EcoRI-200b**<br>TCTGCAGAATTCGGTCGCTGCGTGCAGGGCTCCGCCG	T7-pri-mir-200b dsDNA
pcDNA3	pri-mir-200b (midW_none)	**F-BamHI-200b**<br>CGCGGATCCCGGACCCAGCTCGGGCAGCCGTGGC<br>**R-EcoRI-200b**<br>TCTGCAGAATTCGGTCGCTGCGTGCAGGGCTCCGCCG	T7-pri-mir-200b (midM_none) dsDNA

Supplementary Table 7. The primers for F2 cloning

Primer name	Primer sequence (5'-3')
R-RA3	TTGGCACCCGAGAATTCCA
R-RA3-342-F2	TTGGCACCCGAGAATTCCATTCTGTGTGAGACAATTCCATTAAC
R-RA3-200b-F2	TTGGCACCCGAGAATTCCACCAGGCAGTATTAGAGACCTGACTC

Supplementary Table 8. The primers for qPCR of human pri-miRNAs and miRNAs

Targets	Reverse transcription primers (5'-3')	qPCR primers (5'-3')
Pri-mir-30d (seedM_none, WT)	**R-EcoRI-30d**<br>TCTGCAGAATTCGAGGATGTCTGTGAATAGCCGGTAG	**F-BamHI-30d**<br>CGCGGATCCGGGTCTTAAATTTCTTGTTCAGAAAG<br>**R-EcoRI-30d**<br>TCTGCAGAATTCGAGGATGTCTGTGAATAGCCGGTAG
Pri-mir-30d (seedM6, SNP)	**R-EcoRI-30d**<br>TCTGCAGAATTCGAGGATGTCTGTGAATAGCCGGTAG	**F-BamHI-30d**<br>CGCGGATCCGGGTCTTAAATTTCTTGTTCAGAAAG<br>**R-EcoRI-30d**<br>TCTGCAGAATTCGAGGATGTCTGTGAATAGCCGGTAG
Pri-mir-576 (seedM_none, WT)	**R-EcoRI-576**<br>TCTGCAGAATTCGATGGTTATAACCAATCGAATG	**F-BamHI-576**<br>CGGGATCCGGG GTAACTGCACCATTTTACAATCC<br>**R-EcoRI-576** TCTGCAGAATTCGATGGTTATAACCAATCGAATG

Pri-mir-576 (seedM6, SNP)	**R-EcoRI-576**<br>TCTGCAGAATTCGATGGTTATAACCAATCGAATG	**F-BamHI-576**<br>CGGGATCCGGG GTAACTGCACCATTTTACAATCC<br>**R-EcoRI-576**<br>TCTGCAGAATTCGATGGTTATAACCAATCGAATG
Pri-mir-128-1 (midM_91011, WT)	**R-EcoRI-128-1**<br>TCTGCAGAATTCAAAAAGAAGCCAGGAAGCAGCTG	**F-BamHI-128-1**<br>CGCGGATCCGGGCCTTGTTCCTGAGCTGTTGG<br>**R-EcoRI-128-1**<br>TCTGCAGAATT AAAAAGAAGCCAGGAAGCAGCTG
Pri-mir-128-1 (midM_none)	**R-EcoRI-128-1**<br>TCTGCAGAATTCAAAAAGAAGCCAGGAAGCAGCTG	**F-BamHI-128-1**<br>CGCGGATCCGGGCCTTGTTCCTGAGCTGTTGG<br>**R-EcoRI-128-1**<br>TCTGCAGAATT AAAAAGAAGCCAGGAAGCAGCTG
Pri-mir-885 (midM_none)	**R-EcoRI-885**<br>TCTGCAGAATTCCGGGACTTGAACCCGTGCTCTATCC	**F-BamHI-885**<br>CGCGGATCC GGGCTACTCGGCCCGCACTCTC<br>**R-EcoRI-885**<br>TCTGCAGAATTCCGGGACTTGAACCCGTGCTCTATCC
Pri-mir-885 (midM_1011, SNP)	**R-EcoRI-885**<br>TCTGCAGAATTCCGGGACTTGAACCCGTGCTCTATCC	**F-BamHI-885**<br>CGCGGATCCGGGCTACTCGGCCCGCACTCTC<br>**R-EcoRI-885** TCTGCAGAATTCCGGGACTTGAACCCGTGCTCTATCC
Pri-mir-30a (seedW_none, WT)	**R-30a**<br>TGAAGTCCGAGGCAGTAGGCAGCTGC	**F-T7-30a**<br>TAATACGACTCACTATAGGGTATTGCTGTTGACAGTGAGCGACTG<br>**R-30a**<br>TGAAGTCCGAGGCAGTAGGCAGCTGC
Pri-mir-30a (seedW56)	**R-30a**<br>TGAAGTCCGAGGCAGTAGGCAGCTGC	**F-T7-30a**<br>TAATACGACTCACTATAGGGTATTGCTGTTGACAGTGAGCGACTG<br>**R-30a**<br>TGAAGTCCGAGGCAGTAGGCAGCTGC
Pri-mir-142 (seedW_none, WT)	**R-142**<br>CCGAAGCCCACAGTACACTCATCC	**F-T7-142**<br>TAATACGACTCACTATAGGGCGGACAGACAGACAGTGCAGTC<br>**R-142**<br>CCGAAGCCCACAGTACACTCATCC
Pri-mir-142 (seedW67)	**R-142**<br>CCGAAGCCCACAGTACACTCATCC	**F-T7-142**<br>TAATACGACTCACTATAGGGCGGACAGACAGACAGTGCAGTC<br>**R-142**<br>CCGAAGCCCACAGTACACTCATCC
Pri-mir-16-1	**R-16-1**<br>TAGAGTATGGTCAACCTTACTTCAGC	**F-T7-16-1**<br>TAATACGACTCACTATAGGGTGATAGCAATGTCAGCAGTTTAG<br>**R-16-1**<br>TAGAGTATGGTCAACCTTACTTCAGC
GAPDH	**R-GAPDH**<br>CCAGGGGTCTTACTCCTTGGAG	**F-GAPDH**<br>ACCCACTCCTCCACCTTTGAC<br>**R-GAPDH**<br>CCAGGGGTCTTACTCCTTGGAG
miR-30d (seedM_none, WT)	**miR-30d-5p-RT**<br>GTCGTATCCAGTGCAGGGTCCGAGGTATTCGCACTGGAT ACGACCTTCCT	**miR-30d-5p Fw**<br>GCTGTAAACATCCCCGAC<br>**AllmiR-RW**<br>GTGCAGGGTCCGAGGT
miR-30d (seedM6, SNP)	**miR-30d-5p-RT**<br>GTCGTATCCAGTGCAGGGTCCGAGGTATTCGCACTGGAT ACGACCTTCCT	**miR-30d-5p Fw**<br>GCTGTAAACATCCCCGAC<br>**AllmiR-RW**<br>GTGCAGGGTCCGAGGT
miR-576 (seedM_none, WT)	**miR-576-3p-RT**<br>GTCGTATCCAGTGCAGGGTCCGAGGTATTCGCACTGGAT ACGACGATTCC	**miR-576-3p Fw**<br>GCAAGATGTGGAAAAAT<br>**AllmiR-RW**<br>GTGCAGGGTCCGAGGT
miR-576 (seedM6, SNP)	**miR-576-3p-RT**<br>GTCGTATCCAGTGCAGGGTCCGAGGTATTCGCACTGGAT ACGACGATTCC	**miR-576-3p Fw**<br>GCAAGATGTGGAAAAAT<br>**AllmiR-RW**<br>GTGCAGGGTCCGAGGT
miR-128-1 (midM_91011, WT)	**miR-128-1-3p-RT**<br>GTCGTATCCAGTGCAGGGTCCGAGGTATTCGCACTGGAT ACGACAAAGAG	**miR-128-1-3p Fw**<br>GCTCACAGTGAACCGGT<br>**AllmiR-RW**<br>GTGCAGGGTCCGAGGT
miR-128-1 (midM_none)	**miR-128-1-3p-RT**<br>GTCGTATCCAGTGCAGGGTCCGAGGTATTCGCACTGGAT ACGACAAAGAG	**miR-128-1-0MM-3p Fw**<br>GCTCACAGTGCTACGGT<br>**AllmiR-RW**<br>GTGCAGGGTCCGAGGT
miR-885 (midM_none)	**miR-885-5p-RT**<br>GTCGTATCCAGTGCAGGGTCCGAGGTATTCGCACTGGAT ACGACAGAGGC	**miR-885-5p Fw**<br>GCTCCATTACACTACCCT<br>**AllmiR-RW**<br>GTGCAGGGTCCGAGGT
miR-885 (midM_1011, SNP)	**miR-885-5p-RT**<br>GTCGTATCCAGTGCAGGGTCCGAGGTATTCGCACTGGAT ACGACAGAGGC	**miR-885-5p Fw**<br>GCTCCATTACACTACCCT<br>**AllmiR-RW**<br>GTGCAGGGTCCGAGGT
miR-30a (seedW_none, WT)	**miR-30a-5p-RT**<br>GTCGTATCCAGTGCAGGGTCCGAGGTATTCGCACTGGAT ACGACCTTCCA	**miR-30a Fw**<br>GCTGTAAACATCCTCGAC<br>**AllmiR-RW**<br>GTGCAGGGTCCGAGGT
miR-30a (seedW56)	**miR-30a-5p-RT**<br>GTCGTATCCAGTGCAGGGTCCGAGGTATTCGCACTGGAT ACGACCTTCCA	**miR-30a-56GG Fw**<br>GCTGTAGGCATCCTCGAC<br>**AllmiR-RW**<br>GTGCAGGGTCCGAGGT
miR-142 (seedW_none, WT)	**miR-142-3p-1-RT**<br>GTCGTATCCAGTGCAGGGTCCGAGGTATTCGCACTGGAT ACGACTCCATA	**miR-142-3p Fw**<br>GCGTAGTGTTTCCTACTTTAT<br>**AllmiR-RW**<br>GTGCAGGGTCCGAGGT
miR-142 (seedW67)	**miR-142-3p-1-RT**<br>GTCGTATCCAGTGCAGGGTCCGAGGTATTCGCACTGGAT ACGACTCCATA	**miR-142-3p Fw**<br>GCGTAGTGTTTCCTACTTTAT<br>**AllmiR-RW**<br>GTGCAGGGTCCGAGGT

miR-16-1	**miR-16-1-RT**<br>GTCGTATCCAGTGCAGGGTCCGAGGTATTCGCACTGGATACGACCGCCAA	**miR-16-1 Fw**<br>TAGCAGCACGTAAATA<br>**AllmiR-RW**<br>GTGCAGGGTCCGAGGT
U6	**U6-RT**<br>AAAATATGGAACGCTTCACGAATTTG	**U6 Fw**<br>CTCGCTTCGGCAGCACATATAC<br>**U6 RW**<br>ACGCTTCACGAATTTGCGTGTC

Supplementary Table 9. Plasmids mixture information for miRNA sequencing

Plasmid mixture 1		Plasmid mixture 2		Plasmid mixture 3		Plasmid mixture 14	
**Plasmid name**	**Amount (ug)**	**Plasmid name**	**Amount (ug)**	**Plasmid name**	**Amount (ug)**	**Plasmid name**	**Amount (ug)**
pcDNA empty vector	9	pcDNA_pri-mir-30a	1	pcDNA_pri-mir-30a (seedW_56)	1	pcDNA_pri-mir-342 (midM_910)	1
		pcDNA_pri-mir-142	1	pcDNA_pri-mir-142 (seedW_67)	1	pcDNA_pri-mir-16-1	1
		pcDNA_pri-mir-30d	1	pcDNA_pri-mir-30d (seedM_6)	1	pcDNA empty vector	7
		pcDNA_pri-mir-576	1	pcDNA_pri-mir-576 (seedM_6)	1		
		pcDNA_pri-mir-885	1	pcDNA_pri-mir-885 (midM_1011)	1		
		pcDNA_pri-mir-128-1	1	pcDNA_pri-mir-128-1 (midM_none)	1		
		pcDNA_pri-mir-342	1	pcDNA_pri-mir-342 (midM_none)	1		
		pcDNA_pri-mir-200b	1	pcDNA_pri-mir-200b (midW_none)	1		
		pcDNA_pri-mir-16-1	1	pcDNA_pri-mir-16-1	1		

Supplementary References

1. Nguyen, T. A., Park, J., Dang, T. L., Choi, Y.-G. & Kim, V. N. Microprocessor depends on hemin to recognize the apical loop of primary microRNA. *Nucleic Acids Res.* **46**, 5726–5736 (2018).
2. Kim, K., Duc Nguyen, T., Li, S. & Anh Nguyen, T. SRSF3 recruits DROSHA to the basal junction of primary microRNAs. *RNA* **24**, 892–898 (2018).
