# Supplementary material for: Mismatched and wobble base pairs govern primary microRNA processing by human Microprocessor
Source: Nat Commun. 2020 Apr 21;11:1926. doi: 10.1038/s41467-020-15674-2 (PMC7174388; doi:10.1038/s41467-020-15674-2)
Supplement: Supplementary file 2 — Reporting Summary [file 41467_2020_15674_MOESM2_ESM.pdf]

nature research

Corresponding author(s): Tuan Anh NGUYEN

Last updated by author(s): 03/03/2020

Reporting Summary

Nature Research wishes to improve the reproducibility of the work that we publish. This form provides structure for consistency and transparency in reporting. For further information on Nature Research policies, see Authors & Referees and the Editorial Policy Checklist.

Statistics

For all statistical analyses, confirm that the following items are present in the figure legend, table legend, main text, or Methods section.

n/a	Confirmed	
☐	☒	The exact sample size (n) for each experimental group/condition, given as a discrete number and unit of measurement
☒	☐	A statement on whether measurements were taken from distinct samples or whether the same sample was measured repeatedly
☐	☒	The statistical test(s) used AND whether they are one- or two-sided<br>*Only common tests should be described solely by name; describe more complex techniques in the Methods section.*
☒	☐	A description of all covariates tested
☒	☐	A description of any assumptions or corrections, such as tests of normality and adjustment for multiple comparisons
☒	☐	A full description of the statistical parameters including central tendency (e.g. means) or other basic estimates (e.g. regression coefficient) AND variation (e.g. standard deviation) or associated estimates of uncertainty (e.g. confidence intervals)
☐	☒	For null hypothesis testing, the test statistic (e.g. F, t, r) with confidence intervals, effect sizes, degrees of freedom and P value noted<br>*Give P values as exact values whenever suitable.*
☒	☐	For Bayesian analysis, information on the choice of priors and Markov chain Monte Carlo settings
☒	☐	For hierarchical and complex designs, identification of the appropriate level for tests and full reporting of outcomes
☐	☒	Estimates of effect sizes (e.g. Cohen's d, Pearson's r), indicating how they were calculated

Our web collection on statistics for biologists contains articles on many of the points above.

Software and code

Policy information about availability of computer code

Data collection	Quantification data for gel image collected using Image Lab v6.0.1, pri-miRNA sequences collected from MirGeneDB v2.0., RNA sequencing data collected using Illumina CASAVA
Data analysis	Cutadapt v1.15, fastq-join v1.3.1, FASTX-Toolkit v0.0.13, BWA v0.7.17, Bowtie2 v2.2.9, RNAfold from ViennaRNA Package v2.4.9, Python v3.7.1

For manuscripts utilizing custom algorithms or software that are central to the research but not yet described in published literature, software must be made available to editors/reviewers. We strongly encourage code deposition in a community repository (e.g. GitHub). See the Nature Research guidelines for submitting code & software for further information.

Data

Policy information about availability of data

All manuscripts must include a data availability statement. This statement should provide the following information, where applicable:

- Accession codes, unique identifiers, or web links for publicly available datasets
- A list of figures that have associated raw data
- A description of any restrictions on data availability

The RNA sequencing data has been deposited in the GEO (www.ncbi.nlm.nih.gov/geo/; accession code GSE131636, GSE142140, GSE142136). The source data underlying Figs 1d-e, 2d-i, 3d-i, 4b-c, f-i, k-m, 5d-e,h-o, 6e-f,i-p, 7a-l and Supplementary Figs 1b-c, e, 2b, d-e, 3a-b, d-f, 4b-f, 5b-c, 6e-j, k, 7e-f, h-i are provided as a Source Data file

Field-specific reporting

Please select the one below that is the best fit for your research. If you are not sure, read the appropriate sections before making your selection.

☒ Life sciences ☐ Behavioural & social sciences ☐ Ecological, evolutionary & environmental sciences

For a reference copy of the document with all sections, see nature.com/documents/nr-reporting-summary-flat.pdf

Life sciences study design

All studies must disclose on these points even when the disclosure is negative.

Sample size	3 replicates for RNA sequencing of arti1 library, 3 replicates for each cleavage assay. See method section and figure legends.
Data exclusions	No data was excluded.
Replication	3 replicates for RNA sequencing of arti1 library
Randomization	Not applicable, sample randomization is not relevant to this study.
Blinding	Not applicable, blinding is also not relevant to this study.

Reporting for specific materials, systems and methods

We require information from authors about some types of materials, experimental systems and methods used in many studies. Here, indicate whether each material, system or method listed is relevant to your study. If you are not sure if a list item applies to your research, read the appropriate section before selecting a response.

Materials & experimental systems

n/a	Involved in the study	
☒	☐	Antibodies
☐	☒	Eukaryotic cell lines
☒	☐	Palaeontology
☒	☐	Animals and other organisms
☒	☐	Human research participants
☒	☐	Clinical data

Methods

n/a	Involved in the study	
☒	☐	ChIP-seq
☒	☐	Flow cytometry
☒	☐	MRI-based neuroimaging

Eukaryotic cell lines

Policy information about cell lines

Cell line source(s)	The cell lines (HCT116, HEK293T, HEK293E, TUT4 KO Hela) were the gifts from Dr.Narry KIM's lab, Seoul National University.
Authentication	None of the cell lines were authenticated by our facility.
Mycoplasma contamination	All the cell lines were tested negative for mycoplasma contamination.
Commonly misidentified lines (See ICLAC register)	none used
